# The effectiveness of smoking cessation, physical activity/diet and alcohol reduction interventions delivered by mobile phones for the prevention of non-communicable diseases: A systematic review of randomised controlled trials

**DOI:** 10.1371/journal.pone.0189801

**Published:** 2018-01-05

**Authors:** Melissa Palmer, Jennifer Sutherland, Sharmani Barnard, Aileen Wynne, Emma Rezel, Andrew Doel, Lily Grigsby-Duffy, Suzanne Edwards, Sophie Russell, Ellie Hotopf, Pablo Perel, Caroline Free

**Affiliations:** 1 Department of Population Health, London School of Hygiene and Tropical Medicine, London, United Kingdom; 2 King's Centre for Global Health and Health Partnerships, King’s College London, London, United Kingdom; 3 Division of Women's Health, King’s College London, London, United Kingdom; 4 City and Islington College, London, United Kingdom; 5 Notre Dame Catholic Sixth Form College, Leeds, United Kingdom; 6 The Charter School, London, United Kingdom; 7 Department of Non-communicable Disease Epidemiology, London School of Hygiene and Tropical Medicine, London, United Kingdom; Legacy, Schroeder Institute for Tobacco Research and Policy Studies, UNITED STATES

## Abstract

**Background:**

We conducted a systematic review to assess the effectiveness of smoking cessation, physical activity (PA), diet, and alcohol reduction interventions delivered by mobile technology to prevent non-communicable diseases (NCDs).

**Methods:**

We searched for randomised controlled trials (RCTs) of mobile-based NCD prevention interventions using MEDLINE, EMBASE, Global Health, CINAHL (Jan 1990–Jan 2016). Two authors extracted data.

**Findings:**

71 trials were included: smoking cessation (n = 18); PA (n = 15), diet (n = 3), PA and diet (n = 25); PA, diet, and smoking cessation (n = 2); and harmful alcohol consumption (n = 8). 4 trials had low risk of bias. The effect of SMS-based smoking cessation support on biochemically verified continuous abstinence was pooled relative risk [RR] 2.19 [95% CI 1.80–2.68], I^2^ = 0%) and on verified 7 day point prevalence of smoking cessation was pooled RR 1.51 [95% CI 1.06–2.15], I^2^ = 0%, with no reported adverse events. There was no difference in peak oxygen intake at 3 months in a trial of an SMS-based PA intervention. The effect of SMS-based diet and PA interventions on: incidence of diabetes was pooled RR 0.67 [95% CI 0.49, 0.90], I^2^ = 0.0%; end-point weight was pooled MD -0.99Kg [95% CI -3.63, 1.64] I^2^ = 29.4%; % change in weight was pooled MD -3.1 [95%CI -4.86- -1.3] I^2^ 0.3%; and on triglyceride levels was pooled MD -0.19 mmol/L [95% CI -0.29, -0.08], I^2^ = 0.0%. The results of other pooled analyses of the effect of SMS-based diet and PA interventions were heterogenous (I^2^ 59–90%). The effects of alcohol reduction interventions were inconclusive.

**Conclusions:**

Smoking cessation support delivered by SMS increases quitting rates. Trials of PA interventions reporting outcomes ≥3 months showed no benefits. There were at best modest benefits of diet and PA interventions. The effects of the most promising SMS-based smoking, diet and PA interventions on morbidity and mortality in high-risk groups should be established in adequately powered RCTs.

## Introduction

The World Health Organization (WHO) estimates that 38 million deaths occur each year due to non-communicable diseases (NCDs)—principally cardiovascular diseases, cancer and chronic respiratory diseases. Approximately 42% of NCD deaths are premature (i.e. occur before the age of 70 years) [1]. Although the number of NCD deaths has increased in every world region since 2000, the burden is greatest among people of low socio-economic status. Nearly three-quarters of NCD deaths occur in low and middle income countries [1]. These inequalities also exist within countries, with higher NCD mortality among people with lower education, income, or social class [2].

Physical inactivity, unhealthy diet, tobacco use and the harmful use of alcohol all increase the risk of developing and dying from NCDs. The Global Burden of Disease Study estimated that in 2010, 12.5 million deaths were attributable to dietary risk factors and physical inactivity, over 6 million deaths were attributable tobacco smoking (including second hand smoke), and over 2.5 million deaths were attributable to alcohol use [3]. Encouraging health care consumers to adopt healthy behaviours can prevent the onset or progression of NCDs and reduce mortality [4, 5].

Recent systematic reviews have concluded that there are benefits of interventions delivered by mobile phone targeting smoking cessation, physical activity and diet [6–13]. However, the meta-analyses reported in existing reviews include self-reported outcomes [6–10, 13]. Self reported outcomes in trials of behaviour change interventions where participants are not blind to allocation can be prone to bias and overstated benefits [14]. Some reviews of diet and physical activity interventions included non randomised studies, which are prone to bias [9–12]. In some reviews the effects of interventions delivered partly by mobile phone have been pooled with those delivered wholly by mobile phone, making it impossible to judge the effects of the mobile phone based components [10–12]. Our previous systematic reviews of interventions delivered by mobile phone relied on objective measures of outcomes reported in randomised trials, but the searches for this review were completed in September 2010 [15, 16].

We aimed to provide an updated review of the evidence base for interventions delivered by mobile phone for the prevention of non-communicable disease.

## Methods

This review includes eligible trials identified in the previous comprehensive systematic review that included studies published between 1990 and September 2010 and in further searches conducted to identify studies meeting the inclusion criteria that were published between September 2010 and Jan 2016 in MEDLINE, EMBASE, Global Health and CINAHL [15, 16]. The search strategy for MEDLINE is provided in the Supporting Information (S1 Text). The search terms were adapted for use with other bibliographic databases in combination with database-specific filters for randomised controlled trials, where these were available. Two reviewers independently scanned the electronic records to identify potentially eligible trials. We followed the protocol provided in S2 Text, however, we did not include trials targeting disease management due to resource limitations.

Participants were men and women of any age. We included all controlled trials employing any mobile phone interventions (mobile phones; PDA phones [e.g., BlackBerry, Palm Pilot]; Smartphone [e.g., iphone]) targeting behavioural risk factors for non-communicable diseases, i.e. tobacco use, harmful alcohol use, physical inactivity, and unhealthy diets.

We included studies in which the intervention delivered by mobile phone was the primary intervention component under evaluation. We excluded studies evaluating either mixed mobile phone technology and non-mobile phone technology-based interventions in which the treatment and control group both received the mobile phone technology-based component, or interventions in which treatments between the treatment and control groups differed in additional ways besides the components delivered by mobile phone, such as interventions involving face-to-face counselling with a text message intervention compared to a control group receiving information only. We excluded interventions which can be but do not need to be delivered by mobile phone such as websites, social media and email, except where these were provided in addition to an intervention delivered primarily through mobile phone technology. Interventions employing devices that linked to the mobile phones (e.g. phone-linked activity trackers) were included as these were considered an extension to the mobile phone technology.

For the purpose of this review, primary outcomes were defined as any objective measure of outcomes related to the specified NCD behavioural risk factors, including objective measures of the behaviour and the distal biometric or health effects of the behaviour. For example, objective measures of the behaviour would include salivary cotinine levels for smoking cessation, and step counts for physical activity; biometric measures of effect would include blood pressure, weight, and VO2 max (e.g. for fitness); and health effects would include incidence of diabetes or cardiovascular disease. Secondary outcomes were defined as self-reported measures relating to NCD-related health behaviours, health status, and cognitive outcomes. Studies reporting outcomes for any length of follow-up were included.

Two reviewers carried out the data extraction–this involved each reviewer extracting data independently from half of the studies, and then checking each other’s data extraction against the original papers. The following data was extracted from eligible studies: number of randomised participants, intervention, intervention components, user involvement in intervention development, mobile devices employed, mobile technology functions used, sequence generation, allocation concealment, blinding of outcome assessors, completeness of follow-up, evidence of selective outcome reporting, any other potential sources of bias, and measures of effect using a standardised data extraction form. Where outcomes were measured at multiple time points, we extracted data for the final point of measurement. The authors were not blinded to authorship, journal of publication, or the trial results. All discrepancies were agreed through discussion, and involved a third reviewer when necessary. All analyses were conducted in STATA v 14. We calculated risk ratios and mean differences. We used random effects meta-analysis to give pooled estimates where there were two or more trials using the same mobile technology media (e.g. SMS messages) and targeting the same behaviour (e.g. physical activity) and reporting the same primary outcome. We examined heterogeneity visually by examining the forest plots and statistically using both the χ2 test and the *I*2 statistic.

The behaviour change techniques used in behaviour change interventions were classified according to Abraham and Michie’s taxonomy of behaviour change techniques [17]. Risk of bias of each included study was assessed independently by two study authors according to the criteria outlined by the International Cochrane Collaboration [18]. Disagreements were resolved through discussion, and with input from a third author where necessary. We used a cut off of 90% complete follow-up for low risk of bias for completeness of follow-up. We applied the GRADE criteria [19] to assess the quality for evidence for all outcomes pooled in our meta-analyses.

## Results

**Fig 1:**
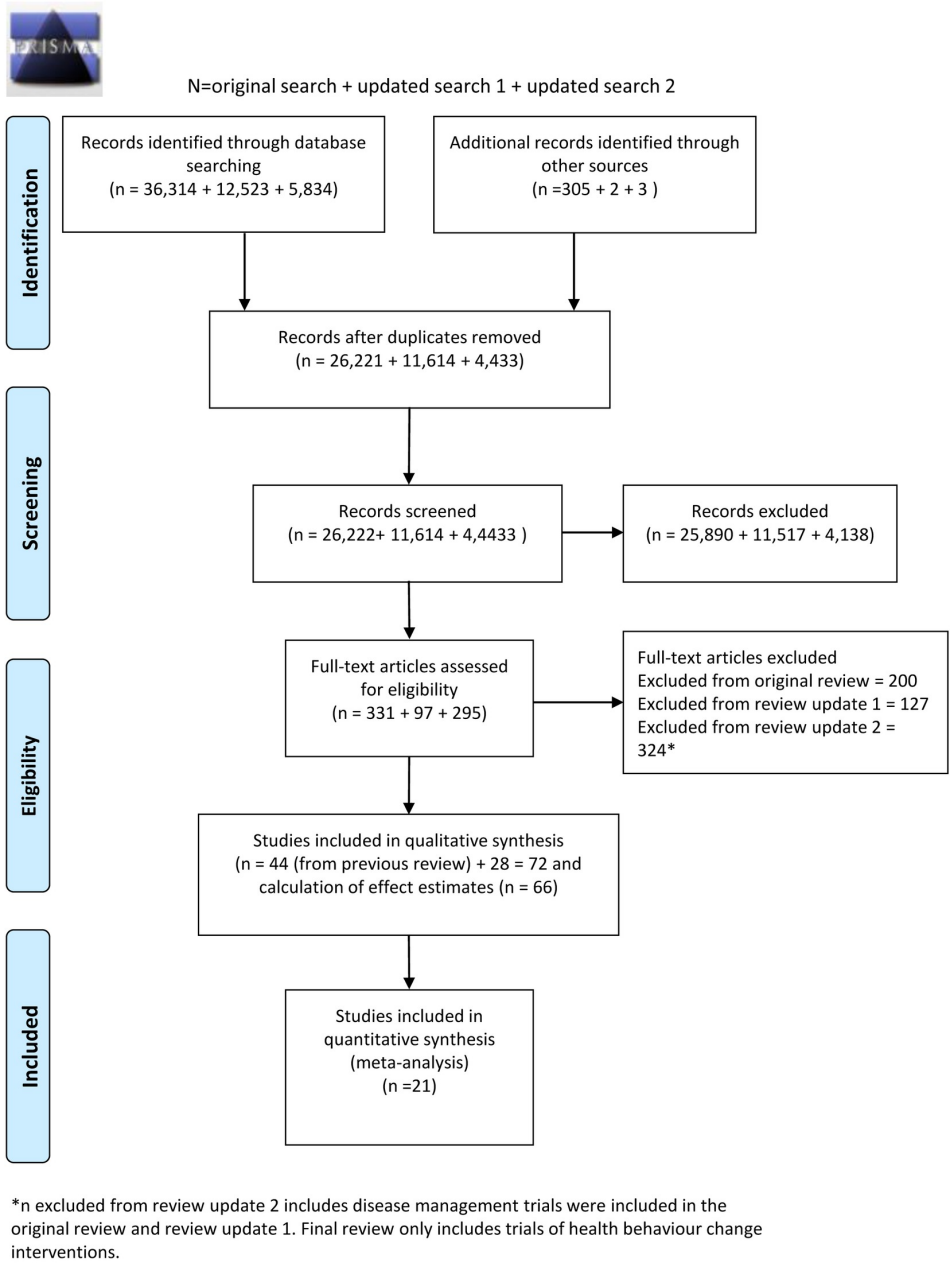
PRISMA flow diagram.

The combined search strategies identified 42,268 electronic records which were screened for eligibility (Fig 1). The full texts of 723 potentially eligible reports were obtained for further assessment. Out of the 723 potentially eligible reports, 72 met the study inclusion criteria and were trials delivered to health care consumers to improve health behaviours. Two papers report on the same trial involving an intervention targeting smoking cessation and an attention-matched control receiving messages promoting improved diet and physical activity. Ybarra (2013) reports on the smoking outcomes and Filion (2015) reports on the physical activity/diet outcomes. Therefore, in total, there were 71 unique trials. 18 interventions aimed to increase smoking cessation; 44 aimed to increase physical activity, improve diet, or a combination of both; 2 aimed to increase physical activity, improve diet, and increase smoking cessation; and 8 aimed to reduce harmful alcohol consumption.

### Characteristics of studies

#### Smoking cessation

There were 18 randomised controlled trials with parallel groups which aimed to increase smoking cessation (Table 1). The smoking cessation trials included a total of 17857 participants, with sample sizes ranging from 31 to 5800. Twelve of the smoking cessation trials were delivered by SMS, three were delivered by voice calls, one by interactive voice response, one by a combination of SMS and video messages, and one by a mobile application combined with voice calls.

**Table 1:** Description of trials of health behaviour change interventions: Smoking cessation.

Study	Study Design, Country, Device, and Media	Participants	Aims	Intervention	Comparator
**Abroms 2014 [21]**	Parallel group RCT; Country: USA; Device: Mobile telephone; Media: SMS	503 adult smokers aged ≥18 y. Mean age: Control 35.5 (SD 10.6); Intervention: 35.9 (10.7). Females: Control 62.8%; Intervention 68.7%. Study advertisements appeared to individual who google searched keywords relating to quitting smoking. Eligibility criteria included having an interest in quitting smoking in the next month.	To evaluate the effect of Text2Quit intervention on biochemically confirmed repeated point prevalence smoking abstinence.	Participants received a facilitated text messaging program designed smoking consisting of automated bidirectional text messages. Text messages were timed around the user’s quit date and provided advice on quitting smoking and were based on social cognitive theory. Messages were interactive and prompted user to track smoking, report on cravings and provide smoking status. Messages were tailored around several factors including first name, quit date, top three reasons for quitting, money saved by quitting, and use of quit-smoking medications. The first three months of intervention offered both outgoing messages and on-demand help through the use of key words. After the outgoing messages stopped, participants could still text for help using keywords. Outgoing messages peaked just prior and following the quit date. SMS messages were supplemented by a personalized web portal (text2quit.com) and emails. Duration: 6 months User involvement in development: not stated.	Participants randomized to the control group initially received a web link to Smokefree.gov, a leading website with quitting smoking information run by the NCI. During the study, Smokefree.gov launched its own texting program, SmokefreeTXT. The control group material was changed to avoid contamination of the control group with a similar texting program. At this point, 135 (26.8%) participants had been recruited into the study, 66 of whom were in the control group. Future control group participants were offered a guidebook on quitting smoking developed by the NCI containing similar advice and information as Smokefree.gov. The control group also received study-related reminder texts via SMS, particularly in the 2 weeks prior to each follow-up survey.
**Borland 2013 [22]**	Parallel group RCT Country: Australia; Device: Mobile telephone; Media: SMS	3530 adult smokers and recent quitters (quit within past 2 weeks) aged ≥ 18 y. Mean age 42.1 y. Females 60% Participants recruited from callers to smoking quitlines (n = 1335), and from an internet panel survey (n = 2195) composed of a mixture of known smokers and those of unknown smoking status. Participants recruited from quitlines were more highly motivated to quit.	To test the population impact of offering automated smoking cessation interventions via the internet and/or by mobile telephone.	Four intervention groups: (1) received a personalised, automated tailored cessation program based on cognitive-behavioural principles that generates 2–4 page letters of advice with suggestions about strategy; (2) received a stream of SMS that mix snippets of advice on strategy and motivation; (3) participants could use either or both parts of interventions 1 and 2 which were offered as a package; (4) participants explicitly offered either or both interventions 1 and 2, with the person encouraged to make an upfront choice although they could subsequently change their minds, and take up whatever aspects they wanted. Duration: 7 months User involvement in development: not stated.	Participants received brief information on web- and telephone-based assistance available in Australia.
**Buller 2014 [23]**	Parallel group RCT Country: USA; Device: Mobile telephone; Media: Application software, SMS	102 adult smokers aged ≥ 18 y. Mean age: Control 24.3 y; Intervention 25.5y. Females: Control 57%; Intervention 45%. Participants recruited through online advertising systems–eligibility criteria included interest in quitting.	To explore the usability, effectiveness and comparability to a SMS programme of a mobile application to support smoking cessation.	Participants were given a smartphone with unlimited phone, SMS and data service on which to use the mobile application. Participants received short messages in an email-like inbox and audio phase-transition messages were delivered to enhance comprehension. Participants could create lists; listen to audio testimonials and read support documents. The short messages directed participants to quitting tools and encouraged them to use the study Website to view additional resources. Duration: 12 wk. User involvement in development: “Two rounds of formative testing of REQ-Mobile with smokers 18–30 years old (59% female) confirmed usability. In Round 1 (n = 17), 76–100% of smokers completed 11 tasks. After REQ-Mobile was revised, 90% of smokers in Round 2 (n = 10) completed seven of nine tasks, and completion times decreased. In both rounds, smokers rated REQ-Mobile favourably.”	Participants were given a smartphone with unlimited phone, text and data service on which they received SMS grounded in social cognitive theory and a modified version of the Transtheoretical model.
**Chan 2015 [24]**	Parallel group RCT; Country: Hong Kong; Device: Mobile phone; Media: SMS	1003 adult daily smokers aged ≥18 y. Age: % 18–39, Control 35.8%, SMS intervention 39.1%. Females: control 16.1%, intervention 19.4%. Participants recruited from ‘Quit to Win Contest’ which aimed to attract and encourage smokers to quit by rewarding them with financial incentives if they had achieved smoking abstinence at a predefined follow-up. >85% of participants reported intention to quit within the next 30 days at baseline.	To examine the effectiveness of brief interventions (3 arms—one delivered by SMS, one delivered by nurse-led telephone counselling, and control) for smokers who joined the Hong Kong Quit to Win Contest to quit smoking.	The SMS group received eight mobile telephone text messages which were constructed with reference to an eight-page smoking cessation booklet received. The content of the messages included (i) warning about the health hazards of smoking, (ii) benefits of quitting, (iii) contact information of publicly available smoking cessation services, (iv) strategies of quitting and (v) encouragement and reminder of follow-up. Duration: 12 months User involvement in development: not stated	The CONTROL group did not receive any intervention above other than the self-help booklet and the contact information of the smoking cessation services at the enrolment.
**Free 2011 [25]**	Parallel group RCT; Country: UK; Device: Mobile telephone; Media: SMS	5800 adult smokers aged ≥ 16 y. Mean age: 36.8 5 y (SD 11.05). Female 45% Recruited through advertising. Eligibility criteria included willingness to make an attempt to quit smoking in the next month.	To evaluate the effect of mobile phone-based SMS support on the point prevalence of smoking at 6 months	Participants received daily SMS before the quit date, then 5 SMS per day for 4 wk after the quit date. Between 4 and 26 wk participants received 3 SMS per week. Message content was tailored to participant interests and concerns about quitting smoking. Participants were offered a quit buddy contactable by mobile phone and an SMS craving helpline with an instant SMS response. The SMS system was fully automated. Duration: 26 wk. User involvement in development: modifications made to intervention based on feedback from 62 participants.	Participants received fortnightly simple, short, generic SMS.
**Free 2009 [26]**	Parallel group RCT; Country: UK; Device: Mobile telephone; Media: SMS	200 adult smokers aged ≥ 16 y. Mean age: 36 y (SD 9.0). Female 38% Recruited through advertising. Eligibility criteria included having interest in quitting.	To evaluate the effect of mobile phone-based SMS support on the point prevalence of smoking at 4 wk.	Participants received daily SMS before the quit date, then 5 SMS per day for 4 wk after the quit date. Between 4 and 26 wk participants received 3 SMS per week. Message content was tailored to participant interests and concerns about quitting smoking. Participants were offered a quit buddy contactable by mobile phone and an SMS craving helpline with an instant SMS response. The SMS system was fully automated. Duration: 26 wk. User involvement in development: as above (Free 2011) modifications made to intervention based on feedback from 62 participants.	Participants received fortnightly simple, short, generic SMS.
**Gritz 2013 [27]**	Parallel group RCT Country: USA; Device: Mobile telephone; Media: Voice calls	474 adult smokers ≥ 18 y. Mean age: Control 45.7 y (SD 7.8); Intervention 43.9 y (SD 8.3). Females 30% Recruited from HIV clinic. Eligibility criteria included willingness to set a quit date within 7 days.	Assess the efficacy of a mobile telephone smoking cessation counselling intervention aimed at a multi-ethnic, economically disadvantaged HIV-positive population.	Participants received usual care (written smoking cessation materials and instructions on how to obtain nicotine patches from the clinic) plus a pre-paid mobile phone on which they received counselling and could access a supportive hotline. Duration: 12 months User involvement in development: not stated	Participants received usual care: written smoking cessation materials and instructions on how to obtain nicotine patches from the clinic.
**Haug 2009 [28]**	Parallel group RCT Country: Germany; Device: Mobile telephone; Media: SMS	174 adult smokers. Mean age: Control 25.4 y (SD 4.9); Intervention 1 (1 SMS) 25.2 y (SD 4.8); Intervention 2 (3 SMS) 24.3 y (SD 3.8). Females: Control 63%; Intervention 1 56%; Intervention 2 52%. Recruited university students who reported daily smoking.	Investigate the feasibility and acceptability of interactive mobile phone text messaging to support smoking cessation and the impact of different SMS frequency (intensity).	Participants received a weekly SMS with a question to assess their stage of change (transtheoretical model). Two intervention groups then received either 1 (1 SMS group) or 3 (3 SMS group) tailored feedback SMS per week. Participants attempting to quit had access to an SMS craving helpline which provided up to 60 tailored SMS responses. The SMS-COACH programme was fully automated. Duration: 14 wk. User involvement in development: not stated.	Participants received only the weekly SMS question to assess their stage of change (transtheoretical model).
**McDaniel 2015 [29]**	Parallel group RCT; Country: USA; Device: mobile phone; Media: IVR	1785 smokers aged ≥18 y recruited from employer and health plan quitline programmes. Mean age: Control 43.3 (SD 12.2); Low intensity IVR 44.0 (SD 11.5); High intensity IVR 43.0 (SD 12.0). Females: control 54.2%, low intensity IVR 54.1%, high intensity IVR 54.2%. Participants were recruited from the Quit For Life programme and must had achieved abstinence for at least 24 hours after their quit date, prior to randomisation.	To test adding an interactive voice response (IVR)-supported protocol to standard quitline treatment to prevent relapse among recently quit smokers.	In the two Technology Enhanced Quitline (TEQ) groups, participants were contacted for relapse risk assessments through automated IVR calls over their first 8 weeks post-quit. Two intensities of IVR monitoring were examined. TEQ-10 participants were contacted twice weekly for the first 2 weeks, then weekly for 6 weeks. TEQ-20 participants were contacted daily for the first 2 weeks, then weekly for 6 weeks. An IVR service contractor programmed and delivered the risk assessments (approximately 5 min), which included questions to identify relapse risk on five factors: lapses, cravings, negative affect, self-efficacy and motivation to remain quit. An algorithm was used to flag participants as ‘at risk’ if they answered any of the screening questions over an established threshold. Participants who exceeded the threshold were then transferred directly to a Quit Coach for a brief intervention (approximately 15 min) specifically addressing the risk factor(s) that triggered their transfer. Duration: 12 months User involvement in development: not stated.	The control group received the standard quitline without IVR monitoring.
**Peng 2013 [30]**	Parallel group RCT Country: Taiwan; Device: Mobile telephone; Media: Voice calls	116 student smokers in Taiwan universities aged ≥ 16 y. Mean age 19.64 (SD 1.337). Females 7.8% Recruited student smokers.	To evaluate the effectiveness of a web phone intervention that delivered phone calls and motivational and educational recorded messages to change smoking behaviour in university student smokers.	Two intervention groups: (1) participants received a phone call at 1 and 9 measuring stage of change, total number of non-smoking days if applicable, self-efficacy and decision balance and also 2 assessment calls per week for 3 wks measuring only stage of change and total number of non-smoking days if applicable; (2) participants received a phone call at 1 and 9 measuring stage of change, self-efficacy and decision balance and also 2 calls per week for 3 wks measuring stage of change and the total number of non-smoking days if applicable. Participants then received 5 to 8 reminder voice messages tailed to address the participant’s stage of change. Duration: 9 wk. User involvement in development: not stated.	Participants received a phone call at 1 and 9 wks for the purpose of measuring stage of change, self-efficacy and decision balance (no intervention given).
**Pollak 2013 [31]**	Parallel group RCT Country: USA; Device: Mobile telephone; Media: Voice calls	31 adult, pregnant smokers in their second trimester aged ≥ 18 y. Mean age: Control 27 y (SD 6); Intervention 29 y (SD 6). Eligibility criteria included willingness to try and quit smoking.	To assess feasibility of a SMS-based smoking cessation intervention for pregnant smokers, to determine the acceptability of a SMS-based SGR plus support messages intervention, and to obtain preliminary efficacy data for SMS-based SGR to promote cessation during pregnancy.	Participants received up to 5 SMS per day for 5 wks. Participants received “alert messages” at scheduled times that instructed participants to smoke and were designed to help them gradually cut down to 0 cigarettes by the end of the fourth week. If participants smoked at an unscheduled time it was requested that they message back “s” so the next alert would correspond with the scheduled interval. If participants messaged 3 times “off schedule,” the alert messages were stopped and resumed the next day. If this pattern continued for more than a day, the participant was called and asked if the schedule needed adjustment. Participants were asked to sign a contract stating they will not smoke more cigarettes during the study than they did before they entered the study. Participants were informed that if they responded to 80% of the alert texts, they would be entered into a $25 gift card raffle. Duration: 6 wk. User involvement in development: not stated prior to this. However, this was a pilot study which was also collecting feedback on intervention from users.	Participants were asked to choose a quit date within 2–3 wks post randomisation and then sent an SMS stating that quit date. Participants received up to 5 SMS per day for 5 wks. Each week, there was a new “theme” for a subset of the messages, such as reasons for quitting, getting ready for the quit date, partner smoking, and handling slips.
**Rodgers 2005 [32]**	Parallel group RCT Country: New Zealand; Device: Mobile telephone; Media: SMS	1705 adult smokers aged ≥ 16 y. Mean age 25 y. Female 58%. Eligibility criteria included interest in quitting smoking within the next month.	Assess the efficacy of SMS for supporting smoking cessation.	Participants set a quit date and received 5 SMS per day for 1 wk before and 4 wk after the quit date. Between 4 wk after the quit date and the end of the study (26 wk) participants received 3 messages per week. SMS contained information or advice on quitting smoking and some distractions (e.g. sports news, quizzes. and polls/surveys). Participants received 1 months of free outgoing text messages after their quit date. Participants were offered a quit buddy (another study participant) contactable by mobile telephone. Participants had access to an SMS craving helpline to receive an instant reply with tips on cravings. The SMS system was fully automated and a computer algorithm was used to match the SMS sent to the participant characteristics. Duration: 26 wk. User involvement in development: “The messages were developed by a multidisciplinary team including young adults, Maori health researchers, and experts in adolescent health, nutrition, cognitive behavioural therapy, and smoking cessation.”	Participants only received one text message every 2 wk, thanking them for being in the study, providing study centre contact details, informing them that those who completed follow-up would be rewarded with a free month of text messaging (whether they quit or not), and reminding them of the time until their free month at the end of follow-up.
**Shi 2013 [33]**	Cluster RCT Country: China; Device: Mobile telephone; Media: SMS, web links via SMS	179 adolescent smokers aged ≥ 16 y. Mean age: Control 16.9 y (SD 0.7); Intervention 17.6y (SD 1.1). Females: Control 15%; Intervention 14%. Recruited adolescents who reported smoking at least 1 cigarette per week for at least 12 weeks.	To test whether a mobile telephone SMS messaging based smoking cessation intervention package could increase the self-reported smoking abstinence and reduce daily cigarette consumption in adolescent smokers from 6 vocational schools in metropolitan Shanghai.	Participants from three intervention schools received interactive smoking cessation intervention delivered via mobile telephone SMS messaging. SMS messages were sent at a time that was common to smoking in the baseline survey every day. A stage-matched protocol was used to provide tailored assistance messages that matched participants’ stage of readiness to quit. Participants were encouraged to send SMS messages to investigators to obtain counselling and clear up confusion. Participants were also given online support by sending web links to their mobile telephones and encouraged to use online chatting relating to their cessation experience. Participants were asked about their smoking status every week and provided feedback depending on whether they were improving or relapsing via SMS. Duration: 12 wk. User involvement in development: “We conducted focus group discussions with adolescent smokers to identify their needs and attitudes towards the intervention.”	Participants received a printed self-help pamphlet based on focus group discussions with adolescent smokers and organised according to the Transtheoretical Model and stages of change.
**Skov-Ettrup 2014 [34]**	Parallel group RCT Country: Denmark; Device: Mobile telephone; Media: SMS	1619 adolescent and young adult smokers aged ≥ 15 y. Mean age: Control 19.5 (SD 3.2); Intervention 19.4 (SD 3.1). Females: Control 58.3%; Intervention 60.3%. Recruited participants from smokers who had signed up to Xhale.dk (an internet- and text message-based smoking cessation service. Eligibility criteria included having set a quit date between 14^th^ February 2007 and 1^st^ August 2009.	To test whether tailored SMS messages delivered at a higher frequency result in higher abstinence among users of an internet-based smoking cessation programme. Also to test whether baseline self-efficacy and beliefs about smoking were predictors of smoking cessation in adolescents and young adults.	Participants received a weekly SMS message up to 4 wks before their quit date, and a daily message 1–3 days before the quit date. Then they received 2 tailored SMS messages per day during a period of 4 wks. For the following 4 wks, the frequency of SMS messages declined to 4–5 SMS messages per wk. The system generated 3 types of tailored messages based on three different tailoring parameters: self-efficacy, beliefs about smoking and themes chosen by the user. Information about these parameters was obtained from the baseline questionnaire. The tailoring was a combination of content matching, descriptive and evaluative feedback. Duration: 12 months User involvement in development: not stated.	Participants received SMS messages to their mobile telephones once daily for 5 wks beginning 5 days before the chosen quit date. Weekly messages were sent for the following 3 wks. All participants received the same message on each day during their attempt counted from their preferred quit date. Messages were personalized by including the participant’s username. Otherwise they were untailored.
**Vidrine 2006 [35]**	Parallel group RCT Country: USA; Device: Mobile telephone; Media: Voice calls	95 HIV positive adult smokers aged ≥ 18 y. Mean age: Control 43.1 y (SD 8.1); Intervention 42.6 y (SD 8.2). Females: Control 17%; Intervention 27.1%. Recruited from HIV care centre. Eligibility criteria included willingness to set a quit date in the next 7 days.	Assess the efficacy of a mobile telephone smoking cessation counselling intervention aimed at a multiethnic, economically disadvantaged HIV-positive population.	Participants set a quit date with their physician and received a personalized smoking cessation plan and a general self-help materials. Participants were given a prepaid mobile telephone and received 8 phone-counselling sessions during 2 months. The counselling sessions were more often close to the quit date. Participants could also call the hotline when they needed additional smoking cessation support. The phone counselling and support was provided by a study researcher. Duration: 3 months User involvement in development: not stated	Participants received usual care: they set a quit date with their physician who offered a 10-wk supply of nicotine replacement therapy and received the personalized smoking cessation plan and a general self-help materials.
**Whittaker 2011 [36]**	Parallel group RCT Country: New Zealand; Device: Mobile telephone; Media: SMS, Video message	226 young adult smokers aged ≥ 16 y. Mean age 27 y (SD 8.7). Females 47% Recruited through advertising. Eligibility criteria included ‘wanting to quit’.	To determine whether a video-based smoking cessation intervention delivered via mobile telephone was effective at increasing smoking cessation rates.	Participants nominated a quit day between 1 and 3 wks from randomisation and 2 time periods during which they wished to receive SMS. Participants were directed to an online brief description and photograph of the 6 role models and asked to select a role model from whom they would receive messages. The video messages were filmed as video diaries during a quit attempt, with the role models discussing issues they had found difficult and the techniques and coping strategies they used to remain smoke-free. The intervention was arranged into a chronological schedule of mobile phone messages that included the role model videos, SMS, and other video messages (animations about reasons to stop smoking; and “truth” campaign mass media advertisements supplied by the American Centres for Disease Control and Prevention). Participants could review video messages they had been sent (and rate them if desired), change their selected time periods, and change selected role model using a website. Participants could also ask for extra support messages on demand by texting keywords to the study shortcode. Duration: 6 months User involvement in development: 180 young people participated in the consultation stage involving focus groups, an online survey, content pre-testing, selection of role models, and a pilot study.	Participants nominated a quit day between 1 and 3 wks from randomisation and 2 time periods during which they wished to receive SMS messages. Participants received one video message every 2 weeks with general health messages and reminders about the study.
**Ybarra 2012 [20]**	Parallel group RCT Country: Turkey; Device: Mobile telephone; Media: SMS	151 adult smokers aged ≥ 18 y. Mean age: Control 35.6 (SD 10.3); Intervention 36.1 (SD 9.5). Females: Control 32%; Intervention 46.1%. Recruited though in-person outreach and advertisements. Eligibility criteria included ‘seriously thinking about quitting in the next 15 days’.	To provide estimates of effect size to better inform a power analysis for a larger trial with a small-scale trial looking at the efficacy of a SMS-based smoking cessation programme in a Middle Eastern setting.	Participants received daily SMS for 6 wks. Participants generally received 5 SMS per day in the pre-quit phase and then received more SMS as the quit day approached. The highest number of messages was sent on the quit day and the day after; and then the number of messages began to taper down until the last week where participants were sent 1 SMS per day. Duration: 3 months User involvement in development: not stated. However this was a pilot trial and included data collection on intervention acceptability and recommendations for improvements.	Participants were given general quitting information in a 7 page brochure.
**Ybarra 2013 [37]**	Parallel group RCT Country: USA; Device: Mobile telephone; Media: SMS	164 young adult smokers aged ≥ 18 y. Mean age: Control 21.6 (SD 2.1); Intervention 21.6 (SD 2.1). Females: Control 44.4%; Intervention 43.6%. Recruited through advertisements. Eligibility criteria included seriously thinking about quitting smoking in the next 30 days.	To develop and pilot test a SMS-based smoking cessation programme for young adults.	Smoking cessation group participants were exposed to a 6-week smoking cessation program with content that was tailored to where participants were in the quitting process (i.e., Day 1 to 14 or the Pre-Quit phase, Day 15 to 21 or the Early Quit phase, or Day 22 to 42 or the Late Quit phase). Participants received SMS at Post-Quit Day 2 and 7 that asked their smoking status. If participants reported smoking, they were sent relapse SMS that focused on helping them recommit to quitting. Frequency of messages varied according to quitting stage. Intervention group participants had access to (a) Another person in the program that a participant was assigned to so they could SMS one another for support anonymously during the programme; (b) Immediate, on-demand SMS aimed at helping the participant through a craving. A project Web site provided additional resources. Duration: 3 months User involvement in development: Stop My Smoking USA intervention was refined for *young* adult smokers in US. Involved needs assessments focus groups (n = 35), content advisory team (n = 10), and tests of technological feasibility (n = 40) with young people aged 18–25.	Participants in the sleep/activity group received text messages at the same rate as the smoking cessation group in order to match the level of attention that the smoking cessation group was receiving; however, content of the text messages was aimed at improving participants’ sleep and physical activity habits within the context of how it would help the participant quit smoking

RCT, randomized controlled trial; SD, standard deviation; SMS, short message service; SCA, smoking cessation adviser; SGR, scheduled gradual reduction.

With the exception of one study carried out in Turkey [20], all trials were conducted in high-income countries.

#### Physical activity

There were 15 randomised controlled trials which aimed to increase physical activity (Table 2). The physical activity trials included a total of 1416 participants, with sample sizes ranging from 36 to 174. In four trials the intervention was delivered though a mobile application, seven trials were delivered by SMS, one through SMS and voice calls, one via fitbit with linked smartphone app, one via SMS with a linked pedometer, and one through audio files uploaded to mobile phone. All physical activity trials were conducted in high income countries.

**Table 2:** Description of trials of health behaviour change interventions: Physical activity.

Study	Study Design, Country, Device, and Media	Participants	Aims	Intervention	Comparator
**Cadmus- Bertram 2015 [38]**	Parallel group randomized Trial Country: USA Device: Fitbit Media: Web based tracking	Participants were 51 overweight, postmenopausal women performing >60 minutes/week of moderate to vigorous physical activity (MVPA) who were regular Internet users, owned a computer/tablet, and could exercise safely. Web-based tracking: n = 25 mean age (SD) 58.6 (6.5) Pedometer group: n = 26 Mean age (SD) 61.3 (7.5)	The aims of this trial are to examine (1) the acceptability of the Fitbit among postmenopausal, overweight/ obese women; and (2) the effect of a Fitbit-based intervention on PA	This study compared (1) a low-touch, Fitbit-based PA intervention focused on self-monitoring/self-regulation skills (Web-Based Tracking Group) and (2) provision of a basic step-counting pedometer (Pedometer Group). Both were asked to perform 150 minutes/week of MVPA and walk 10,000 steps/day. Participants in the web-based tracking group received a Fitbit One, an accelerometer-based device that clips to the waistband or bra, or is placed in a pocket. Summary data are shown on the tracker’s display and PA intensities and temporal patterns are available on the website. The intervention was based on the Coventry, Aberdeen, and London—Refined (CALO-RE) framework, involving self-monitoring, combined with other self-regulatory skills (e.g., goal setting, frequent behavioural feedback). Duration: 16 wks User involvement in development: not stated	Pedometer group participants received a basic pedometer and printed materials with tips for increasing steps. They also completed a brief goal-setting process, based on steps observed on the ActiGraph.
**Direito 2015 [39]**	Parallel three Group RCT Country: New Zealand Device: Smart phone Media: Mobile application for improved fitness (AIMFIT)	51 young people aged 14–17 year olds no achieving PA recommendations (60 mins of MVPA daily) Immersive app Intervention: 17 Mean age: 15.78(1.11) Female: 9 Non immersive app intervention: 16 Mean age: 15.69 (1.04) Female: 10 Control group: 18 \Mean age: 15.55(1.32) Female:10	To determine the effects of 2 smartphone apps (zombies, Run and Get Running) on cardiorespiratory fitness of insufficiently active healthy young people. And secondly to see which of the features of the app design may contribute to improved fitness and PA levels	The 2 intervention groups both received an app which contained a fully automated 8 week training program designed to improve fitness and ability to run 5km. The first group received an immersive app (zombies, run!) which featured a game themed design where the training program was imbedded into a story. The second group received a non-immersive app (Get Running-coach to 5k). Participants we’re encouraged to use their app 3 times per week and work their way through the workouts. Duration: 8 wk User involvement in development: not stated–commercially available apps.	Control group was asked to continue with their usual physical activities and were not offered any information about increasing PA.
**Glynn 2014 [40]**	Parallel group RCT; Country: Ireland; Device: Mobile telephone; Media: Application software	90 android smart users from rural primary care in the west of Ireland. Mean age: 44 y (SD 11); Control 46 y (SD 11); Intervention 42 y (SD 11). Females: 64%; Control 51%; Intervention 78%	To evaluate the effectiveness of a smartphone application to increase physical activity in primary care.	Participants were given access to a smartphone application and instructed in its usability features, encouraged to try to achieve 10,000 steps per day as an exercise goal and given an exercise promotion leaflet. Duration: 8 wk User involvement in development: not stated–commercially available apps.	Participants were encouraged to walk an additional 30 minutes per day along with their normal activity (the equivalent of 10,000 steps) as an exercise goal and given an exercise promotion leaflet.
**Kim 2013 [41]**	Parallel group RCT; Country: USA; Device: Mobile telephone; Media: SMS	36 African-Americans aged ≥ 60 y. Mean age: Control 70.5 y (SD 7.5); Intervention 69.3 y (SD 7.3). Females; Control 80%; Intervention 81%	To examine if a motivational mobile phone SMS intervention would increase step count among older community-dwelling African Americans. A secondary aim was to study the effects of SMS on self-reported leisure-time exercise behaviour.	Participants received a pedometer and a walking instructional manual. Participants received 3 motivational SMS per day, 3 days per wk. Duration: 6 wk User involvement in development: states that texts were modelled on those used previous research. The previous research referenced included 42 women users writing texts related to weight management.	Participants received a pedometer and a walking instructional manual.
**Liu 2008 [42]**	Parallel group RCT; Country: Taiwan; Device: Mobile telephone; Media: MP3/audio	60 male COPD patients aged ≥ 40 y in stable condition. Mean age: Control 72.8 y (SD 1.3); Intervention 71.4 y (SD 1.7)	Document the clinical efficacy, compliance and applicability of a home-based exercise training programme supervised via a mobile phone.	Participants performed daily endurance exercise training by walking in speed with music played on a mobile phone. The tempo of the music was changed as appropriate every 3 months as fitness changed. Duration: 1 y User involvement in development: not stated.	Participants were provided with a home rehabilitation programme booklet and a DVD, including written instructions for home walking exercise training. Reinforcement telephone call every 2 weeks for first 3 months
**Maddison 2014 [43]**	Parallel group RCT; Country: New Zealand; Device: Mobile telephone; Media: SMS	171 adults aged ≥ 18 y with IHD. Mean age 60.2 (SD 9.3); Control 59 (SD 9.5); Intervention 61.4 (8.9). Females 19%	To determine the effectiveness and cost-effectiveness of a mobile telephone- delivered exercise CR programme for people with IHD to improve exercise capacity and physical activity levels compared to current services.	Participants were free to participate in any CR service or support that they wished to use. This typically involves participating in community-based CR education sessions, encouragement to be physically active and an offer to join a local cardiac club that provides supervised exercise. Participants received a personalized, automated package of SMS aimed at increasing exercise behaviour. Duration: 24 wk. User involvement in development: 38 CR patients and 3 CR nurses in formative qualitative research (focus groups and interviews). 20 CR patients completed online survey to provide feedback on content.	Participants were free to participate in any CR service or support that they wished to use. This typically involves participating in community-based CR education sessions, encouragement to be physically active and an offer to join a local cardiac club that provides supervised exercise.
**Martin 2015 [44]**	Parallel group two phase RCT; Country: USA. Device: Accelerometer and mobile phone. Media: SMS	48 outpatients attending an academic CVD prevention centre aged 18–69 years who used a ‘Fit-bug’ (accelerometer)-compatible smartphone. Mean age: Control: 60 (SD 7); Intervention (no texts): 58 (SD 8); Intervention (texts) 55 (SD 8). Females: Control: 44%; Intervention (no texts): 44%; Intervention (texts) 50%.	To evaluate whether a fully automated mobile health (mHealth) intervention with tracking and texting components would increase physical activity.	The mHealth intervention had 2 core components—tracking and texting. Participants were randomized to unblinded and blinded tracking. Unblinded tracking participants had continuous access to activity data through a smartphone interface. Participants in the unblinded group were then randomised to either receive smart texts or no texts. Smart texts provided smartphone-delivered coaching 3 times/day aimed at individual encouragement and fostering feedback loops by a fully automated, physician-written, theory-based algorithm using real-time activity data and 16 personal factors with a 10 000 steps/day goal. Intervention group 1: unblinded activity tracker and smart texts Intervention group 2: unblinded activity tracker and no texts. Study occurred over two study phases after the 1-week blinded run-in. In the first 2-week phase, participants were randomized to unblinded or blinded tracking. In the second 2-week phase, the unblinded participants were randomized to receive smart texts or no texts. Duration: 5 wks User involvement in development: ‘physician-designed mhealth intervention’.	Control group were allocated to blinded activity tracking and no text messages.
**Newton 2009 [45]**	Parallel group RCT; Country: New Zealand; Device: Mobile telephone; Media: SMS	78 adolescents aged 11–18 y; attending outpatient clinic in 4 regional adolescent diabetes services in New Zealand	To assess whether pedometers and text messaging increase physical activity in adolescents with type 1 diabetes	Participants wore an open pedometer with a daily goal of at least 10,000 steps. Participants received a weekly motivational text message reminding them to wear the pedometer and be active. Duration: 12 wk User involvement in development: not stated.	Standard diabetes care.
**Nguyen 2009 [46]**	Parallel group RCT; Country: USA; Device: PDA phone; Media: Application software, SMS, Voice	17 COPD patients aged 40 y with stable condition. Mean age: Intervention 1 (Mobile-C) 72 y (SD 9); Intervention 2 (Mobile-SM) 64 y (SD 12). Females: Intervention 1 67%; Intervention 2 63%	Determine the feasibility and efficacy of a mobile phone-based exercise persistence intervention for patients with COPD following completion of pulmonary rehabilitation	Participants were asked to do 150 min of moderate-intensity exercise per week in 3 to 5 sessions and received booklets with exercise tops, local resources, and pictures of exercises. Two intervention groups: (1) submitted daily information about their COPD symptoms and exercise via SMS on the PDA phone and received 1 SMS reply per week to thank them for the information only; (2) submitted COPD symptom and exercise information and received personalized weekly feedback and encouragement SMS written by the study nurse or telephone calls to discuss problems in more detail. Duration: 6 months User involvement in development: not stated.	None
**Petrella 2014 [47]**	Parallel group RCT; Country: Canada. Device: smartphone, Bluetooth enabled blood pressure monitor, glucometer, pedometer.	149 adults aged 18–70 years with at least two metabolic syndrome risk factors. Mean age: Control 57.8 (SD 8.7); Intervention 55.7 (SD 10.1). Females: Control: 75.7%; Intervention: 73.3%.	To investigate the effects of a mHealth supported exercise intervention compared to an active control group receiving only the exercise prescription over a short-term (12-week) period; and second, to examine the long-term maintenance over 24 and 52 weeks of follow-up.	Participant fitness was assessed and a tailored exercise program was prescribed by an exercise specialist according to the Step Test and Exercise Prescription (STEP™) protocol. In addition to the exercise prescription, participants in the mHealth intervention group received a kit, which included a smartphone data portal (Blackberry® Curve 8300 or 8530) equipped with Healthanywhere health monitoring application (Biosign Technologies Inc., Markham, Ontario, Canada), a Bluetooth™ enabled blood pressure monitor (A & D Medical, UA-767PBT, San Jose, California, USA), a glucometer (Lifescan One Touch Ultra2™, Milpitas, California, USA) with Bluetooth™ adapter (Polymap Wireless, PWR-08-03, Tucson, Arizona, USA) and a pedometer (Omron, HJ-150, Kyoto, Japan). The mHealth technologies were provided primarily as a self-management tool. The home-monitoring protocol required participants to input pedometer steps and exercise daily; measure blood pressure and fasting blood glucose three times per week; and measure body weight with their own home scale once per month. Duration: 52 wks User involvement in development: not stated.	Following a light, standardized snack, participants’ fitness was assessed and a tailored exercise program was prescribed by an exercise specialist according to the Step Test and Exercise Prescription (STEP™) protocol.
**Prestwich 2009 [48]**	Parallel group RCT; Country: UK; Device: Mobile telephone; Media: SMS	155 adults aged ≥ 18 y who exercise less than 3 times per wk. Mean age 23.8 (SD 4.6). Females 58%	Test whether the effects of implementation intentions on exercise can be strengthened by combining them with text message reminders	Participants read recommendations about exercising for 20 min at least 3 times per week. Three intervention groups: (1) formed implementation intentions for achieving the exercise goal and were offered opportunity to receive SMS reminder of these plans; (2) SMS reminders to exercise (no implementation intentions formed); (3) formed implementation intentions for achieving the exercise goal only. Duration: 4 wk User involvement in development: not stated.	Two control groups: (1) no treatment; (2) read recommendations about exercising for 20 min at least 3 times per week and were informed of the benefits of forming implementation intentions (but were not asked to form any).
**Prestwich 2010 [49]**	Parallel group RCT; Country; UK Device: Mobile telephone; Media: SMS	149 adults with low physical activity levels. Mean age: Control 23.6 y (SD 4.5); Intervention 1 (SMS plan reminder) 22.2 y (SD 5.0); Intervention 2 (SMS goal reminder) 24.4 y (SD 6.9). Females: Control 68%; Intervention 1 56%; Intervention 2 64%.	Test whether interventions that paired implementation intentions with text messages reminders of plans or goals increased brisk walking in a student-based sample	Participants set goals for and formed implementation intentions for walking for at least 3 min 5 or more days per week. Two intervention groups: (1) received SMS reminders of the plans; (2) received SMS reminders of the brisk walking goal. Participants received at least 1 SMS reminder during the 4-wk study and could change the content, time and frequency of SMS through a secure website. Duration: 4 wk User involvement in development: not stated.	Participants were asked to try to walk briskly for at least 3 min on ≥5 d per week to meet recommended physical activity levels.
**Sirriyeh 2010 [50]**	Parallel group RCT; Country: UK; Device: Mobile telephone; Media: SMS	120 participants aged between 16 and 19 y, in full time further education and in possession of a personal mobile phone.	To develop and pilot the feasibility and efficacy of a novel intervention using affective messages as a strategy to increase PA levels in adolescents.	The SMS text messages for the single instrumental intervention group included statements regarding the instrumental gains associated with regular moderate and vigorous PA. Examples of these messages are, “Physical activity can help maintain a healthy weight. What activity will you do today?” and “Physical activity can keep your heart healthy. What activity will you do today?” For the combined affective and instrumental intervention group, SMS text messages included statements regarding either the affective, or instrumental, gains associated with regular moderate and vigorous PA. An equal number of messages from interventions 1 and 2 were presented in the intervention period. Duration: 2 wk. User involvement in development: small pilot study was used to assess the face validity of the messages amongst 15 sixth form students.	Participants also received SMS text messages over the same 14-d period. However, this was limited to two messages (1 per week). The content of these messages was neutral, using only the final element of the phrase used in the intervention groups, “What activity will you do today?” for comparability.
**Wang 2015 [51]**	Parallel Group RCT Country: America Device: Fitbit one, Smart phone, Accelerometer (Actigraph and GT3X+) Media: SMS	67 adults aged 18–69 years with a BMI ≥25 kg/m2 doing <150 min/week of MVPA and had ability of improving PA, non-smokers. Mean age: control 47.1 (SD 11.9); intervention 49.3 (SD 11.5). Females: control 94%; intervention 88%.	To investigate the use of a wearable sensor/device (Fitbit One) and short message services (SMS) text messaging to increase PA in overweight and obese adults.	Participants completed baseline questionnaire, were weighed and measured. They then set a PA agenda with 5 min intervention setting goals. Study personnel demonstrated how to wear the Fitbit one and Actigraph GT3X+, how to upload data and how to access daily summaries. Intervention group wore Fitbit one and received SMS text messages. Participants wore Fitbit 1 and Actigraph GT3x+ concurrently for 7 days to access baseline PA levels. Valid day: 600 min/day. They also received 3 automated text messages a day for 6 weeks and were asked to upload their data everyday throughout the study. Duration 6 wks. User involvement in development: not stated.	Control group were also asked to continue to wear the Fitbit One tracker and upload data every day for the duration of the study.

#### Diet

We identified three trials which aimed to improve diet (Table 3), including a total of 906 participants with sample sizes ranging from 41 to 808. In one trial the intervention was delivered through a mobile application, in one trial through SMS with a web-based tool, and one was delivered through personalised emails sent to smartphones based on participants’ salt intake. All three trials focusing on diet were carried out in high income countries.

**Table 3:** Description of trials of health behaviour change interventions: Diet.

Study	Study Design, Country, Device, and Media	Participants	Aims	Intervention	Comparator
**Morikawa 2011 [52]**	Parallel group RCT; Country: Japan; Device: Mobile telephone; Media: email	41 hypertensive males working in a railroad company. Mean age: Control 47.1 y (SD 8.5); Intervention 48.3 y (SD 8.7)	To investigate the effectiveness of a workplace salt reduction intervention program on BP using an electronic salt sensor to monitor daily salt excretion and communication via personalised emails sent to mobile telephones.	Participants received initial counselling on lifestyle modification, measured their daily salt excretion during 1st and last week of intervention and received personalised guidance to reduce salt intake via smartphone email 10 times. Duration: 4 wk. User involvement in development: not stated.	Participants received initial counselling on lifestyle modification.
**Soureti 2011 [53]**	Parallel group RCT; Country: UK; Device: Mobile telephone, Personal computer; Media: SMS, web-based tool	808 overweight adults aged ≥ 50 y. Mean age 46 y (SD 8.6); Control 45.9 y (SD 8.4); Intervention 1 46.2 y (SD 8.6); Intervention 2 45.8 y (SD 8.7).	To explore the combined impact of a Web-based, fully automated planning tool and SMS reminders on intention to change saturated fat intake, self-reported saturated fat intake, and portion size changes over 4 wk.	2 intervention groups: (1) Participants used a web-based planning tool to select from a list of 13 situations in which they were tempted to eat unhealthily and then choose an approach to change their behaviour from a list of 13 solutions. Participants were asked to complete at least 3 situation-solution pairs. Once these pairs were chosen and saved, participants were not able to revisit the program to change them during the 4-week period. (2) Participants used the same web-based planning tool as in Intervention 1. After completing the planning session, participants then entered their mobile number and chose a time band to receive text reminders of their plans. Duration 4 wks User involvement in development: not stated.	Participants received educational information on the importance of a healthy diet low in saturated and on the association between high cholesterol and being overweight. At weeks 1 and 5, they filled out online questionnaires.
**Wharton 2014 [54]**	Parallel group RCT; Country: USA. Device: Smartphone; Media: Application	57 adults aged 18–65 years with normal BMI (25–40 kg/m^2^) recruited from a campus community. Mean age: Control 40.8 (SE 3.8); Iphone app intervention group 43.7 (SE 3.5); Smartphone memo intervention group 41.5 (SE 4.0). Females: Control 26.7%; Iphone app intervention group 68.4%; Smartphone memo intervention group 15.4%.	To assess the use of a popular smartphone app for dietary self-monitoring and weight loss compared to traditional diet counselling and entry methods.	Participants randomized into 3 groups: the app group (AP), trained to use the diet-tracking ‘‘Lose It!” app daily; the memo group (ME), trained to track dietary intake daily through use of the memo function on their smartphones; and the paper group (PA), trained to record dietary intake daily using a traditional paper-and-pencil method. The AP group recorded dietary intake using the ‘‘Lose It!” app interface, which provided a large database of commonly consumed foods for users to search and add to a diary at each eating occasion. It also provided immediate feedback in the form of a daily calorie gauge graphic that increased in real-time as foods were entered. The ‘‘Lose It!” app calculated the daily energy allotment for the user based on a pre-identified weight loss goal (1 lb/wk) and individual anthropometric data. No dietary advice was provided to the AP group; however, these participants received immediate feedback regarding calorie intake when dietary data were entered into the ‘‘Lose It!” app. Duration 8 wks User involvement in development: not stated–commercially available app.	The paper group (PA), trained to record dietary intake daily using a traditional paper-and-pencil method.

#### Physical activity and diet

There were 26 trials which targeted both physical activity and diet (Table 4). These trials included a total of 4092 participants, with sample sizes ranging from 24 to 502. Eleven trials tested interventions delivered via SMS, six trials were of interventions delivered through mobile applications, eight involved interventions delivered by a combination of media such as SMS, MMS, mobile apps, podcasts, and/or voice calls, and one trial was of an intervention delivered through voice calls alone. Twenty-four of physical activity and diet trials were conducted in high income countries. The other two trials were carried out in India [55] and Pakistan [56].

**Table 4:** Description of trials of health behaviour change interventions: Physical activity and diet combined.

Study	Study Design, Country, Device, and Media	Participants	Aims	Intervention	Comparator
**Allen 2013 [57]**	Parallel group RCT; Country: USA; Device: Mobile telephone; Media: Application software	68 obese adults aged ≥ 21 y. Mean age 44.9 y (SD 11.1). Females 78%	To evaluate the feasibility, acceptability, and preliminary efficacy of theoretically based behavioural interventions delivered by smartphone technology.	Three intervention groups: (1) Diet and exercise counselling plus self-monitoring smartphone intervention; (2) a less intensive diet and exercise counselling plus self-monitoring smartphone intervention; (3) self-monitoring smartphone intervention only. Duration: 6 months User involvement in development: not stated–commercially available app.	The control received an established intensive healthy eating and exercise counselling intervention from a nutritionist coach weekly for the first month and biweekly for the 2nd to the 6th month. No smartphone app.
**Brindal 2013 [58]**	Parallel group RCT; Country: Australia; Device: Mobile telephone; Media: Application software	58 overweight/obese adults aged ≥ 19 y. Mean age 42 y. Females 100%	To develop and evaluate weight-loss intervention application software for mobile telephones that supported individuals embarking on a diet and that was evidenced-based.	Participants were instructed to follow a commercially available meal replacement programme. Participants received meal replacement application software. During the first 4 wks meal replacements were provided and in the second 4 wks participants had to purchase their own if they wanted to continue. The application software provided information, simplified food intake recording, rewarded positive behaviour and prompted regular interaction through reminders and self-monitoring of weight and diet. Duration: 8 wk. User involvement in development: not stated.	Participants were instructed to follow a commercially available meal replacement programme. Participants received application software that reproduced the information available with the meal replacement programme.
**Carter 2013 [59]**	Parallel group RCT; Country: UK; Device: Mobile telephone; Media: Application software	128 overweight adults aged ≥ 18 y. Mean age 42 y (SD 9); Intervention 41.2 y (SD 8.5); Control 1 42.5 y (SD 8.3); Control 2 41.9 y (SD 10.6). Females 77%	To test the acceptability and feasibility (recruitment, dropout, and adherence) of weight loss application software for mobile phones with a view to informing a larger trial.	Participants were given a HTC Desire mobile phone with the weight loss application software pre-downloaded. Participants received standardized training in the equipment. Participants were instructed to use the study equipment every day for a week and then to use it as much as they desired over the trial period. Participants were given access to an Internet forum for social support. Duration: 6 months User involvement in development: not stated.	Two control groups: (1) Participants were given a paper food diary, a calorie-counting book, and a calculator; (2) Participants were given a voucher providing 6 months access to the Weight Loss Resources website. Participants in Control groups 1 and 2 were instructed to use the study equipment every day for a week and then to use it as much as they desired over the trial period and were given access to an Internet forum for social support.
**Cowdery 2015 [60]**	12-week randomized, controlled, parallel-group trial Country: USA Device: mobile phone Media: Interactive APP	40 participants between the ages of 18 and 69, had regular access to a smartphone with the Android (4.0 or later) or iPhone (IOS 7.0 or later) platform, had no physician-imposed limitations on physical activity, and had not had a myocardial infarction, coronary artery bypass surgery, or coronary stenting procedure within the prior 5 years Median age: control 32.0 (IQR 25.0, 45.5); intervention 31.5 (23.5, 41.8). Females: control 80%; intervention 90%.	The purpose of this study, therefore, was to test whether Exergame smartphone applications encourage and increase participation in physical activity, specifically walking and jogging. It was hypothesized that adults randomized to receive the Exergames for 12 weeks would have a greater increase in physical activity compared to a control group. Additional aims included the examination of the impact of the use of Exergame apps on enjoyment of exercise and motivation to exercise.	The Exergame apps (Zombies, Run! and The Walk) selected based on the preliminary study. These are both commercially available action adventure games. Zombies, Run! is an immersive running game and audio adventure that instructs players to collect supplies and avoid being attacked by Zombies as they exercise. The Walk is an audio adventure game that presents episodes and challenges to the player, who is tasked with a package that must be delivered in order to save the world. Participants randomized to the intervention group had one of the 2 Exergame apps placed on their smartphones. Participants were given a choice between the 2 apps. Participants were instructed to use the apps for the next 12 weeks when they walked or ran for exercise. Participants were also assisted in the download of an activity tracking app (MOVES). Participants in the intervention group also received weekly motivational e-mails. Duration: 3 months User involvement in development: not stated–commercially available app.	Control group participants did not receive the exergame apps or the motivational e-mails. Participants in the control group were similarly assisted in downloading the activity tracking app (MOVES) to their smartphones. This app continuously monitored their activity (frequency, duration, intensity, and distance) and thus was visible to all participants and allowed the study team to download activity data from the MOVES website.
**De Niet 2012 [61]**	Parallel group RCT; Country: Netherlands; Device: Mobile telephone; Media: SMS	141 overweight/obese children aged ≥ 7 y. Mean age 9.9 y (SD 1.3); Control 9.8 y (SD 1.3); Intervention 10.1 y (SD 1.3). Females 64%; Control 66%; Intervention 62%.	To evaluate whether a mobile phone SMS intervention during a paediatric lifestyle intervention positively affects BMI-SDS, and reduces treatment dropout after 1 year in overweight/obese children. Also to evaluate compliance with the intervention.	Participants began the mobile phone SMS intervention 3 months into their paediatric lifestyle intervention. Participants received a mobile phone and were instructed on its use. Participants were asked to send weekly self-monitoring data on relevant parameters via SMS. Participants could also SMS if they felt in need of communication about any positive or negative life events, thoughts, or feelings. Participants received tailored SMS feedback in response. Compliance was enhanced by sending an SMS reminder after 1 week of non-responding. Duration: 9 months User involvement in development: not stated.	A paediatric lifestyle intervention with no additional mobile phone SMS intervention at 3 months.
**Fassnacht 2015 [62]**	8 week cluster RCT. Country Portugal Device: Mobile phone. Media: SMS	49 participants aged 8–10 in 2 4^th^ grade school classes in Braga Portugal. Mean age: control 9.6 (SD 0.4); intervention 9.5 (SD 0.3). Females: control 66.7%; intervention 36.4%.	Aimed to explore participants' adherence to and satisfaction with the SMS-based monitoring and feedback system. A secondary aim of the pilot study was to explore the preliminary efficacy of the program to promote health behaviours.	Children were instructed to report data in a standard format via SMS using their parent's mobile phones. The children received feedback messages. The feedback messages aimed to motivate and encourage children to reach each behavioural goal and to support and reinforce positive development based on improved or deteriorated behaviour. Feedback messages had a limit of 160 characters. Duration: 2 months User involvement in development: not stated.	All children participated in 2 60-minute educational sessions presented in a group format and facilitated by 2 trained psychologists. The only material that differed between the intervention and control groups was presented in session 2, which included detailed information about and training with the SMS program. Session 1 focused on increasing physical activity, decreasing screen time, and the risks of sedentary behaviour. Session 2 focused on a healthy diet in general and the importance of the consumption of fruits and vegetables specifically.
**Filion 2015 [63]**	Secondary analysis of data from the Stop My Smoking USA (Ybarra, 2013) Parallel group RCT Country: USA; Device: Mobile telephone; Media: SMS	164 young adult smokers aged ≥ 18 y. Mean age: Control 21.6 (SD 2.1); Intervention 21.6 (SD 2.1). Females: Control 44.4%; Intervention 43.6%.	This study examined the effectiveness of a text message-based active control intervention in improving sleep and physical activity habits among a U.S. national sample of young adult smokers participating in a smoking cessation intervention.	Smoking cessation group participants were exposed to a 6-week smoking cessation program with content that was tailored to where participants were in the quitting process (i.e., Day 1 to 14 or the Pre-Quit phase, Day 15 to 21 or the Early Quit phase, or Day 22 to 42 or the Late Quit phase). Participants received SMS at Post-Quit Day 2 and 7 that asked their smoking status. If participants reported smoking, they were sent relapse SMS that focused on helping them recommit to quitting. Frequency of messages varied according to quitting stage. Intervention group participants had access to (a) Another person in the program that a participant was assigned to so they could SMS one another for support anonymously during the programme; (b) Immediate, on-demand SMS aimed at helping the participant through a craving. A project Web site provided additional resources. Duration: 3 months User involvement in development: User involvement for smoking messages (described for Ybarra, 2013, above). For the sleep/activity arm, text messages were initially developed by Ybarra and colleagues and then underwent expert review.	Participants in the sleep/activity group received text messages at the same rate as the smoking cessation group in order to match the level of attention that the smoking cessation group was receiving; however, content of the text messages was aimed at improving participants’ sleep and physical activity habits within the context of how it would help the participant quit smoking
**Haapala 2009 [64]**	Parallel group RCT; Country: Finland; Device: Mobile telephone; Media: SMS	125 overweight (BMI 25–36 kg/m^2^) adults aged 25–44 y. Mean age: Control 38 y (SD 4.7); Intervention 38.1 y (SD 4.7). Females: Control 76%; Intervention 79%.	Investigate the effectiveness of a mobile phone weight-loss programme among healthy overweight adults	Participants received a daily automated SMS indicating a daily target weight and progress to that goal, the percent reduction in food consumption compared to normal diet and total target Kcals, days remaining until reaching target weight. Participants also received SMS tips on how to reduce calorie intake/increase physical activity. Participants could adjust the target weight at 3-monthly clinic visits. Participants also had access to a secure website to view weight loss progress. Duration: 1 year. User involvement in development: not stated–commercially developed weight loss programme ‘Weight Balance’ (GeraCap Invia Ltd, Seinajoki, Finland).	No treatment and only had monitoring clinic visits at baseline and 12 month follow-up.
**Hebden 2014 [65]**	Parallel group RCT; Country: Australia; Device: Mobile phone; Media: SMS, email, application and internet forum	51 University students and staff aged 18–35 years. Mean age: control 23.1 y (SD 3.7); Intervention 22.6 y (SD 5.4). Females: control 76%; intervention 85%.	To measure the effect of a mHealth intervention programme on body weight, body mass index (BMI) and the specific lifestyle behaviours addressed by the programme [i.e. physical activity and sedentary behaviour, intake of fruit and vegetables, energy-dense takeaway meals and sugar sweetened beverages (SSB)] compared to a control group.	Text messages were tailored to the processes of change identified in the Transtheoretical Model and moved from addressing cognitive to behavioural processes to facilitate movement through stages of change. Participants received each text as an e-mail at the same time and date, written in full without ‘text talk’. Some e-mails contained additional information that was referred to in the corresponding text message Application Four smartphone applications were developed by the investigators (one per behaviour). Each application enabled users to record their behaviour (e.g. daily minutes of physical activities performed, daily servings of fruit and vegetables, or weekly frequency, and energy and fat content, of takeaway meals) and to then receive instantaneous tailored motivational advice, as well as feedback in reference to population health guidelines. Duration: 3 months User involvement in development: text messages–not stated. Applications– 10 participants provided qualitative feedback on prototype versions.	Booklet and session with a dietitian
**Kim 2015[66]**	Parallel group RCT; Country: Korea; Device: mobile phone: Media: SMS	205 male employees of public institutions undergoing standardized annual medical examinations at hospitals. Mean age: control 41.55 (SD 6.98); intervention 41.02 (SD 6.82). Females: control 0%; intervention 0%.	To examine the efficacy of a tailored text-messaging intervention for obese male participants in a worksite weight loss program of 6 months duration.	A text message-based application that was tailored to participants’ individual dietary behaviours and physical activity levels using responses to questionnaires and metabolic risk profiles that were assessed by laboratory examinations and anthropometric measurements. Duration: 6 months. User involvement in development: Not stated—Three family physicians, 1 psychiatrist, and 2 dietitians collaborated to develop text message content.	The comparison group received identical support as the intervention group with the exception of not receiving automatic tailored text messages.
**Laing 2014 [67]**	Parallel group RCT; Country: USA; Device: Mobile telephone; Media: Application	212 adults with BMI of 25 kg/m^2^ or more. Mean age: Control 43.2 (SD 15); Intervention 43.1 (14). Females: Control 76%; Intervention 70%.	To evaluate the effect of introducing primary care patients to free smartphone app for weight loss.	Research assistants help intervention group participants download the MyFitnessPal (MFP) app on to their smartphone and showed an instructional video developed by MFP. These participants also received a telephone call from the same research assistant 1 week after enrolment to assist with any technical problems with the app. MFP was designed by software engineers in collaboration with dietitians to create an app for calorie counting. The app provides a database of foods for logging food and exercise. Users enter their current weight, goal weight, and goal rate of weight loss (limited to 0.23 to 0.90 kg/wk). The MFP app then shows the user their daily, individualized calorie goal. MFP also generates real-time reports showing users their weight trend, caloric intake in the past week, and nutritional summaries of their diet (for example, grams of fat, carbohydrates, and protein and milligrams of sodium). The app also includes a bar code scanner for store-bought foods and a social networking feature that enables users to find friends and share their progress. Study participants were encouraged to use the social networking feature with friends and to set reminders to log their food. Duration: 6 months User involvement in development: not stated–publicly available app.	Research assistants told control group patients to “choose any activities you’d like to lose weight,” without specifying any particular interventions. Control group participants were aware that they were participating in a study of a weight-loss app but were blinded to the name of the app. To minimize contamination of the control group, providers and clinic staff were also blinded to the name of the app and to group assignment. At the 3-month follow-up visit, all participants received a 1-page educational handout on healthy eating from www.myplate.gov.
**Lin 2015 [68]**	Parallel group RCT; Country: USA; Device: Mobile telephone; Media: text messages	124 African American adults aged ≥21 years, with a body mass index >27 recruited from churches. Mean age: Control 52.3 (SD 12.0) Intervention 49.2 (SD 12.7). Females; Control 78.7%; Intervention 90.5%.	To investigate whether a behavioural theory-based mobile health intervention would enhance weight loss when added to standard care among overweight/obese African American adults.	The intervention group received the same assessment, recommendations, and materials as the standard care control group, but also received an automated, 6-month text message program tailored at enrolment by participants’ selection of the 3 most personally relevant goals among 8 options. For 6 months, participants were sent messages pertaining to these targeted behaviours, customized to each participant’s wake, lunch, and sleep times. Duration: 6 months (for primary outcome), final follow-up at 12 months. User involvement in development: Not stated.	Participants received an initial clinical assessment consisting of a 20-minute one-on-one session with a dietitian, a visit with a study physician to review their health status, educational materials on diet and physical activity, and a digital pedometer. The dietitian constructed a weight control plan for each participant based on the US Department of Agriculture Food Guide Pyramid and consensus governmental exercise recommendations. Participants were asked to return for follow-up visits at 3, 6, and 12 months. At the 3-month visit, in-person biometric assessment was conducted, and participants received additional educational materials. At the 6-month visit, participants received additional educational materials and a brief feedback session with a dietitian.
**Martin 2015 [69]**	Parallel group RCT; Country: USA; Device: Mobile phone (smartphone); Media: text, email, phone calls, remote monitoring	40 overweight and obese adults (BMI 25-35kg/m^2^) aged 18 to 65 years. Mean age: control 43.3 (SD 2.63); intervention 45.6 (SD 2.67). Females: control 85%; intervention 80%.	Test the efficacy of SmartLossSM, a smartphone-based weight loss intervention, in a pilot study.	The SmartLoss platform provides remote monitoring of progress and the delivery of personalized treatment recommendations and lesson material via the multimedia capabilities of smartphones. SmartLoss participants were prescribed a low calorie diet and received guidance on gradually increasing physical activity, with a goal of achieving 10,000 steps/day, consistent with the guidelines of national organizations to achieve this goal. The participant was instructed to weigh daily on a bathroom scale provided to them that automatically and wirelessly transmitted their data from the scale to a transceiver on an Internet-enabled computer, which then transmitted the data to a website that was accessible by their counsellor. Duration: 3 months User involvement in development: not stated.	Participants in the attention-matched Health Education control group received health information via text messages or e-mails delivered to the smartphone. Topics included suggestions for stress management, healthy eating, exercise, and sleep hygiene.
**Napolitano 2013 [70]**	Parallel group RCT; Country: USA; Device: Personal computer, Mobile telephone; Media: Electronic social networking, SMS	24 overweight/obese university students aged ≥ 18 y. Mean age 20.47 y. Females 86.5%.	To evaluate the feasibility, acceptability, and preliminary efficacy of a novel, technology-based weight loss intervention for college students using adapted evidence-based weight-loss content.	Two intervention groups: (1) Participants received information about the private Facebook group and privacy settings. Participants were ‘‘friended” by the private group and instructed to accept the request. The private Facebook group served as the portal to access the intervention content (i.e. handouts and podcasts). Participants also had access to polls and healthy activity or eating events to which they could respond. Participants were alerted to the availability of new intervention content via group postings and Facebook mail. Participants were encouraged to gradually increase their physical activity with the target of engaging in moderate intensity exercise for at least 250 min per week; (2) Participants received access to a private Facebook group with the same content described for Intervention 1 (separate groups were used for interventions 1 and 2 to limit cross contamination). Participants also received additional theoretically-driven intervention targets: goal-setting, self-monitoring, and social support communicated via SMS. Duration: 8wk User involvement in development: not stated.	Waiting list controls
**Patrick 2013 [71]**	Parallel group RCT; Country: USA; Device: Mobile telephone, Personal computer; Media: SMS, website, emails, voice	101 overweight/obese adolescents aged ≥ 12 y. Mean age 14.3 y (SD 1.5). Females 36.6%	To evaluate the effectiveness of an intervention delivered through combinations of three modalities: the web, group sessions for adolescents and parents, and SMS in overweight/obese adolescents.	Three intervention groups: (1) Participants received weekly “check-in” emails, monthly mailed tip sheets, and access to the program website and its web tutorials. (2) Participants had access to the program website and its web tutorials, monthly mailed tip sheets, and monthly 90 min group sessions of 5–10 adolescents and their parents where they discussed the behavioural skills from the web tutorials. Participants received brief (~20 min) bimonthly phone calls from the health counsellor reviewing concepts presented in the web tutorial and reinforcing behavioural strategies. Attendance and participation in the group sessions were rewarded with mileage incentives and a lottery for prizes such as cookbooks or other materials to assist with healthy behaviour change. (3) Participants had access to the program website and its web tutorials, monthly mailed tip sheets, and a minimum of 3 SMS per wk that related to weekly challenges and intervention goals. Reminder SMS were sent if the participant did not log on to the website by the fourth day of the intervention. Participants could also communicate via SMS with a health counsellor if they had questions. Participants were provided with mobile telephones and prepaid SMS plans. Duration: 12 months Nutrition demonstrations and physical activities were also integrated in each group session. User involvement in development: During the development phase content was “piloted and revised after input from a diverse group of adolescents regarding reading level, understanding of concepts, ability to hold their attention, and usability of information.”	Participants were given printed materials produced by the American Diabetes Association and the American Heart Association. Participants were encouraged to attend three 1 h group nutrition sessions during the first 6 wks. Participants received monthly tip sheets by mail. This reflected the prevailing community standard of care.
**Ramachandran 2013 [55]**	Parallel group RCT; Country: India; Device: Mobile telephone; Media: SMS	537 working men with impaired glucose tolerance aged ≥ 35 y. Mean age: Control 46.1 y (SD 4.6); Intervention 45.9 y (SD 4.8)	To assess whether SMS that encouraged lifestyle change could reduce incident type 2 diabetes in Indian Asian men with impaired glucose tolerance.	In addition to standard lifestyle modification advice, participants received SMS at frequent intervals. SMS contained information about healthy lifestyle, the benefits of physical activity and diet, cues to start physical activity and healthy dietary practices, and strategies to avoid relapse and remain motivated to maintain physical activity and healthy dietary habits. SMS content was based on the transtheoretical model of behavioural change. Duration: 2 years User involvement in development: not stated.	Participants received standard lifestyle modification advice. This consisted of personalised education and motivation about healthy lifestyle principles, and written information about diet and physical activity.
**Shahid 2015 [56]**	Parallel group RCT; Country: Pakistan; Device: mobile phone; Media: phone calls	440 participants aged 18–70 years of age, residing in rural areas of Pakistan, type-2 Diabetes Mellitus, HbA1c ≥8.0% presenting to the hospital outpatient endocrinology services. Mean age: control 49.21 y (SD 7.92); intervention 48.95 y (SD 8.83). Female: control 38.6%; intervention 38.6%.	To determine the effect of mobile phone intervention on HbA1c in type-2 Diabetes Mellitus (DM) patients living in rural areas of Pakistan.	The intervention group patients were called directly on mobile phone after every 15 days for a period of 4 months. They were asked about the self-monitoring blood glucose, intake of medications, physical activity, healthy eating and were physically examined. Duration: 4 months User involvement in development: not stated.	Both groups were given a glucometer and asked to monitor their glucose levels. The control group was examined initially and after 4 months physically in the clinic and there were no mobile phone contacts with these patients.
**Shapiro 2012 [72]**	Parallel group RCT; Country: USA; Device: Mobile telephone, Personal computer; Media: SMS, MMS, website	170 overweight/obese adults aged ≥ 21 y. Mean age 41.9 y (SD 11.8): Control 40.9 y (SD 12.1); Intervention 43.1 y (SD 11.4). Female 65%: Control 64%; Intervention 67%	To evaluate an SMS intervention for weight loss.	Participants received SMS and MMS 4 times/day. SMS included: tips, facts, motivation, messages requesting answers to knowledge questions, or self-monitoring data on weight and steps. MMS included portion control pictures and weight/step graphical feedback over time. Participants were given a pedometer and those who did not have a scale were given a digital scale. SMS for self-monitoring data requested step count (daily) and weight (weekly). Participants received personalized feedback on progress via: 1) weekly weight and step graphical MMS charts that depicted the previous 5 weeks; and 2) a daily pedometer goal for the upcoming week. Participants received monthly e-newsletters with diet and PA information from credible publicly available sources. They also had access to a website that provided health tips, recipes, food and PA logs, and a personal weight chart. Duration: 12 months User involvement in development: intervention ‘text2diet’ modified Patrick et al’s (2009) content. According to Patrick et al: “Designing the system began with formative research with overweight men and women to solicit feedback about dietary behaviours, current mobile phone and text and picture message habits, the type and frequency of text and picture messages helpful for weight loss, and nutrition-related topic areas that should be included in a weight loss program. Focus group participants also tested prototypes of the system”	Participants received monthly e-newsletters with diet and PA information from credible publicly available sources. Participants were given a pedometer and those who did not have a scale were given a digital scale.
**Shapiro 2008 [73]**	Parallel group RCT; Country: USA; Device: Mobile telephone; Media: SMS	58 children aged 5–13 y. Mean age: Control 1 (group sessions) 8.5 y (SD 2.3); Control 2 (group sessions and paper diary) 9.3 y (SD 2.2); Intervention 8.4 y (SD 2.3). Females: Control 59%; Intervention 72%.	Assess the impact of SMS on self-monitoring behaviours related to weight management in children and on dietary and physical activity behaviour change	Families attended 3 educational group sessions on increasing physical activity, reducing sugar-sweetened beverage consumption and screen time (computer or television). Parent and child pairs were instructed to send 2 SMS daily to the study team, reporting separately for the parent and child: the number of steps taken (measured by pedometer); the number of sugar-sweetened beverages consumed; the minutes of screen time. Parents and children received automated feedback messages selected from a database using an algorithm based on the number of goals met, comparison to the previous day. Duration: 8 wk. User involvement in development: not stated.	Families attended the same educational group sessions as the intervention group. There were 2 control groups: (1) attended group sessions only; and (2) attended group sessions and recorded target behaviours in a paper diary
**Shaw 2013 [74]**	Parallel group RCT; Country: USA; Device: Mobile telephone; Media: SMS	120 adults receiving treatment at a residential weight loss management programme who had lost 5% of body weight since entering programme. Mean age 52 y (SD 15.5): Control 54.8 y (SD 15.9); Intervention 1 51.0 y (SD 12.9); Intervention 2 54.3 y (SD 15.5). Females: Control 64%; Intervention 1 63%; Intervention 2 50%	To evaluate the acceptability, feasibility, and efficacy of daily SMS using regulatory focus theory to help individuals sustain weight loss.	Two intervention groups: (1) Participants received messages that targeted regular physical activity, a low calorie healthy diet and monitoring of body weight behaviours needed to sustain weight loss. SMS content focused on promoting success and rewarding oneself. (2) Participants received messages that targeted regular physical activity, a low calorie healthy diet and monitoring of body weight behaviours needed to sustain weight loss. SMS content focused on preventing failure and avoiding temptations. Duration: 30 days User involvement in development: pilot study with 16 participants who gave qualitative feedback on intervention.	Participants received general health SMS for 30 days following the weight loss program.
**Steinberg 2013 [75]**	Parallel group RCT; Country: USA; Device: Mobile phone; Media: SMS	50 women aged 25–50 years, with a body mass index (BMI) greater than or equal to 25 kg/m2. Mean age: 38.3y	To evaluate the feasibility of a text messaging intervention for weight loss among predominantly black women.	The intervention (Shape Plan) included daily tracking of tailored behaviour change goals through text messaging and personalized daily and weekly feedback via text messaging and email, respectively. Participants also received information sheets about behavioural goals, a pedometer, 2 face-to-face group sessions, and a skills training DVD. Duration: 6 months User involvement in development: not stated.	To control for contact and isolate the behaviour change goals, self-monitoring via text messaging and feedback, participants randomized to the education control arm received (1) 2 in-person group education sessions, one at baseline and another at 6 months; (2) a set of videos at 3 months that covered topics such as healthy eating patterns, eating cues, recognizing hunger, exercise recommendations, and how to read a nutrition facts food label; (3) pedometers; and (4) a “prescription” to walk 10,000 steps per day. Control arm participants received no text messaging during the study period, but had the option to receive a 3-month version of the text messaging intervention after the study was complete.
**Svetkey 2015 [76]**	Parallel group RCT; Country: USA. Device: Smart phone; Media: Application	365 young adults aged 18–35 years with BMI ≥ 25 kg/m^2^. Mean age: Control 29.6 (SD 4.3); Smartphone intervention group: 29.2 (SD 4.2). Females: Control 69.1%; Intervention group: 68.9%.	To determine the effect on weight of mobile technology-based (mHealth) behavioural weight loss interventions in young adults.	The intervention was delivered through an investigator-designed smartphone app which included goal setting, challenge games, and social support through a “buddy system” that allowed exchange of pre-determined messages to a randomly assigned buddy participant. Self-management behaviours for CP were regularly and frequently prompted by the app according to a protocol-driven schedule; participants did not have a choice in the timing or frequency of prompts. Tailoring within the CP intervention occurred mainly via setting personal goals. Self-monitoring by smartphone was achieved by tracking weight, dietary intake, and physical activity, with frequent prompts to self-monitor and feedback on the results. Duration: 24 months User involvement in development: “Both interventions were designed with input from the target population obtained through focus groups that were conducted in the year before the trial began.” Six focus groups with 33 participants. However not clear to what extent this informed content of intervention or only strategies for recruitment.	Participants randomized to the Control group were given three handouts on healthy eating and physical activity from the Eat Smart Move More NC program (http://www.eatsmartmovemorenc.com/) but otherwise received no intervention and were not asked to self-monitor.
**Turner-McGrievy 2011 [77]**	Parallel group RCT; Country: USA; Device: Mobile telephone, personal computer; Media: SMS, Podcast, Application software, Electronic social networking	96 overweight/obese adults aged ≥ 18 y. Mean age: Control 43.2 y (SD 11.7); Intervention 42.6 y (SD 10.7)	To examine whether a combination of podcasting, mobile support communication, and mobile diet monitoring can assist people in weight loss.	Participants received the same Podcasts as in the comparison group. Participants were also instructed to download a diet and physical activity monitoring application and a social networking site’s (Twitter) application to their mobile device. Participants created a user account on Twitter, were told to log on to Twitter at least once daily to read messages posted from the study coordinator, and were encouraged themselves to post at least daily to Twitter. Participants could choose any user name they wanted (to protect their identity) and were instructed on how to make their Twitter account private (if they chose to do so). During months 0–3, participants were divided into 4 groups to create Twitter cohorts of 11–12 people. They were sent a list of everyone’s Twitter user names within their cohort, were instructed to follow everyone in their cohort, and were reminded to send follow requests to participants and to accept requests until everyone in each cohort was following one another. During months 3–6, participants were asked to follow everyone in the study, and similar procedures were used to allow everyone within the group to follow one another. The study coordinator sent out 2 messages per day to the group, which reinforced messages from the podcasts, posed questions to the group to facilitate discussion, and encouraged participants to share tips and recipes with one another that would assist in weight loss. Such messages were prompts to attend to weight-loss behaviour, and encouraged communication but were not individualized. The study coordinator did not participate in discussions initiated by participants. All participants received information on safe exercise practices. Duration: 6 months. User involvement in development: Not stated—App was commercially available (fatsecret.com), podcast developed by research team.	Participants received 2 podcasts per wk for 3 months (approximately 15 minutes each) and 2 mini-podcasts per week for months 3–6 (approximately 5 minutes each). Participants had access to a group-specific podcast site, where they could subscribe to the podcast using their mobile device or listen directly to the podcast on a computer. Podcasts delivered in the first 3 months contained a section on nutrition and physical activity information, an audio blog of a man or a woman trying to lose weight, a soap opera, and a goal-setting activity. Podcasts delivered in months 3–6 contained only the nutrition and exercise portion of the podcast and focused on overcoming barriers and problem-solving issues. Participants received a book with calorie and fat gram amounts of food to assist them in monitoring their dietary intake.
**Van der Weegen 2015 [78]**	Three arm cluster RCT. Country: Netherlands. Media: mobile phone and tablet. Device: mobile phone. Media: app.	24 practices agreed to participate. Participants were between 40 and 70 years old with DM2 or COPD, and who did not, comply with Dutch Norm for Healthy Exercise (having at least 30 minutes of moderate to vigorous physical activity on 5 or more days of the week). Additional inclusion criteria for the DM2 patients was a body mass index (BMI) >25, and for the COPD patients, a clinical diagnosis of COPD according to the GOLD-criteria stage 1–3, known to be stable in their respiratory function for at least 6 weeks, and on a stable drug regimen. Participants needed to be able to access a computer with an Internet connection and master the Dutch language sufficiently. Group 1 received the complete intervention (monitoring and feedback tool and SSP), practices in Group 2 received the SSP only, whereas practices in Group 3 received care as usual. Group 1 n = 65 Mean age 57.5 (SD 7.0); Female 52.3%; Group 2 n = 66 Age mean 56.9 (SD 8.3); Female 47%. Group 3 n = 68 Age mean 59.2 (SD 7.5); Female 54.4%.	Aim: to evaluate the longitudinal effects of this multifaceted intervention on 40–70 year-old patients with chronic obstructive pulmonary disease (COPD) and diabetes type 2 (DM2) in primary care. Furthermore, the additional effect of using this tool on top of the SSP was evaluated.	SSP: The program consisted of four individual consultations with the PN; in the first week, after 2 weeks, after 2–3 months, and after 4–6 months. First, the participants received an information booklet about the course of the intervention containing the Short Questionnaire to Assess Health-Enhancing PA and a list of locally organized PA activities. The tool consists of a three-dimensional (3D) activity monitor, a mobile phone app, and a Web app. Participants were asked to wear the activity monitor on a daily basis. They could see their real-time activity results and history in minutes of moderate to vigorous activity on the mobile phone and Web app, in relation to a personal goal. The personal activity goal was set in the second consultation of the SSP. Hereafter, automated feedback messages were sent related to the personal goal. Moreover, the participant was asked in a dialogue session to set up an activity plan to achieve the daily goal. During the entire intervention, activity results and answers to dialogue sessions were visible for the PN on a secured Web app. Duration: 9 months User involvement in development: intervention development informed by interviews and focus groups with 15 patients and 16 care professionals. Further usability and pilot testing with users (n = 10).	Usual care
**Van Drongelen (2014) [79]**	Parallel Group RCT Country: Netherlands Device: Smart Phone/Tablet Media: Mobile application and website	502 pilots owning smartphone or tablet. Could not participate if on sick leave for more than 4 weeks at start of recruitment. Intervention: 251 Average Age: 41.0 (SD 8.0) Female:21% Control: 251 Average age: 40.7 (SD 8.7) Female: 13%	To evaluate effect of mHealth intervention regarding exposure to daylight, sleep, physical activity and nutrition aiming to improve health-related behaviour thereby reducing sleep problems and fatigue and improve health perception of airline pilots,	Participates took part in a baseline questionnaire. They were then emailed with login details to a mobile application and a secure section of the project website which could only be entered by intervention group. The app contained advice tailored to flight schedules and personal characteristic to reduce fatigue and circadian disruption. They could choose personal advice for the preparation from home, time spent during layover abroad and arrival home. They could also choose advice based on sleep and daylight exposure, nutrition and physical activity. Participants also received timed reminders from the app if they had not been on it for longer than 3 weeks and geofencing alerts (If the participant arrived outside the Netherlands, max. 1 alert per 4 days) Duration: 6 months User involvement in development: app developed after developed after focus group interviews with a random sample of 30 pilots, and interviews with key management stakeholders of the airline company.	Control group were given minimal intervention allowing the access to a secure part of the project website which contained basic, non-tailored, fatigue and health-related information that was already available within the airline company.
**Varnfield 2014 [80]**	Parallel group RCT. Country: Australia. Device: Mobile phone. Media: smartphone monitoring, text messages, audio and video files. Platform included a web portal with participant data for mentors to provide weekly consultations.	94 patients from Primary & Community Health Services. All post-MI patients referred to CR were considered for participation. Subjects excluded if they were unable to participate in self-management programmes due to medical care needs, operate smartphone for purposes of trial (e.g. vision, hearing, cognitive or dexterity impairment) or attend TCR, or were involved in another trial or had no experience with mobile/smartphones. Mean age: control 56.2 (SD 10.1); intervention 54.9 (SD 9.6). Female: control 17%; intervention 9%.	This study aims to investigate the effect of a smartphone-based home service delivery (Care Assessment Platform) of CR (CAP-CR) on CR use and health outcomes compared with a traditional, centre-based programme (TCR) in post-MI patients	The CAP-CR platform used a smartphone for health and exercise monitoring, and delivery of motivational and educational materials to participants via text messages and preinstalled audio and video files (including understanding cardiovascular disease (CVD), symptoms and management). The platform included a web portal with participant data for mentors to provide weekly consultations. Each participant was equipped with a smartphone (Nokia N96, Nokia Inc) preinstalled with health diary (WellnessDiary, Nokia Research) and activity monitoring (StepCounter, Nokia Research) applications; blood pressure (BP) monitor (AXIS Pacific C/-Delmond flexibles Pty Ltd); and weight scale (Glass Body Analysis scale, Propert). Activity monitoring (step number, duration and intensity) was automatic through the phone’s in-built accelerometer. All participants received detailed programme information and 1 h of face-to-face training on technology use (supported by a device instruction manual) and technical phone support during the trial if required. Duration: 6 months User involvement in development: not stated–programme developed according to National Heart Foundation of Australia and the Cardiac Society of Australia and New Zealand guidelines.	The TCR programme comprised of two supervised exercise and 1 h educational sessions on a weekly basis for 6 weeks at one of four Health Service District community centres. Participants started education sessions once enrolled to CR and twice-weekly exercise sessions commenced once centre appointments became available. Participants followed an individualised, supervised, circuit-based exercise programme of light (6–10) to moderate (11–13) intensity according to Borg’s scale. The programme included cardiovascular and strengthening routines involving, for example, treadmill, rower, resistance bands, weights, squats and modified push-ups
**Wong 2013 [81]**	Parallel group RCT; Country: China; Device: Mobile telephone; Media: SMS	104 adult professional drivers. Mean age: Control 55.2 (SD 6.5); Intervention 54.1 y (SD 6.1). Females: Control 4%; Intervention 9.3%	To determine the efficacy of using SMS to provide IGT and DM knowledge and to reduce the risk of developing T2DM at 12 and 24 months among Chinese professional drivers with pre-diabetes.	Participants received the same information booklets as the comparison group. Participants were sent SMS grouped under four broad themes: (i) information about diabetes and pre-diabetes, (ii) information about lifestyle modification, (iii) social norms of how others would appreciate the lifestyle modification and (iv) self-efficacy enhancing statements of how to control and stay on behaviour control. One SMS was randomly sent to participants at specified times as follows. In the first 3 months, SMS were sent 3 times a week. In the subsequent 3 months, SMS were sent once per week. In the subsequent 6 months and in the subsequent 12 months, SMS were sent once per month. The sequence of the SMS sent was generated randomly by computer. Duration: 2 y User involvement in development: not stated.	Participants received information booklets on pre-diabetes, diabetes, and health behaviour information when they had their baseline laboratory (oral glucose tolerance test and full lipid profile) results. Participants were treated with usual care by their own doctors.

BMI, body mass index; BMI-SDS, body mass index standard deviation scores; COPD, chronic obstructive pulmonary disorder; FB, feedback; PA, physical activity; RCT, randomized controlled trial; SD, standard deviation; CR, cardiac rehabilitation; IHR, ischaemic heart disease; IGT, impaired glucose tolerance; DM diabetes mellitus; T2DM, type 2 diabetes mellitus.

#### Physical activity, diet, and smoking

There were 2 trials [82, 83] which targeted physical activity, diet, and smoking cessation as part broad lifestyle interventions (Table 5). One, conducted in Australia, included 710 respondents and trialled an intervention delivered by SMS [82], and the other, conducted in Iran, involved 180 participants and assessed the effectiveness of an app-based intervention [83].

**Table 5:** Description of trials of health behaviour change interventions: Physical activity, diet and smoking cessation.

Study	Study Design, Country, Device, and Media	Participants	Aims	Intervention	Comparator
**Chow 2015 [82]**	Parallel-design, single-blind, randomized clinical trial Country: Australia Device: Mobile telephone Media: text messages	710 participants recruited at a large tertiary referral center and university teaching hospital in Sydney, Australia. Patients were eligible if they were older than 18 years and had documented CHD. Mean age: control 57.3 (SD 9.3); intervention 57.9 (SD 9.1). Females: control 17.6%; intervention 18.5%.	The study aimed to evaluate, in a randomized clinical trial, the effect of a text message–based intervention to encourage lifestyle change on objective measures of cardiovascular risk in individuals with coronary heart disease (CHD).	The text message–based prevention program involved delivery of regular semipersonalized text messages providing advice, motivation, and information that aimed to improve diet, increase physical activity, and encourage smoking cessation (if relevant). Participants received 4 messages per week for 24 weeks. Each message was sent on 4 of 5 randomly selected weekdays and arrived at random times of the day during working hours. Duration: 6 months User involvement in development: 53 participants completed questionnaire for evaluation of message content, usefulness, and language. 16 participants involved in pilot testing and provided feedback.	Control participants received usual care, which generally included community follow-up with the majority referred to inpatient cardiac rehabilitation, as determined by their usual physicians. Both groups received 3 study management text messages providing them with their allocation assignment, study contact details, and a reminder prior to the follow-up appointment.
**Golshahi 2015 [83]**	Parallel group 4-arm RCT; Country: Iran; Device: mobile phone; Media: SMS	180 hypertensive patients referring to two major clinics of Isfahan University of Medical Sciences (IUMS) and two health centers in Isfahan, Iran. 4 arms, 45 in each: A: patients and their family educated by cardiology resident about self-care behaviours through eight sessions. Mean age: 56.72 B: obtained self-care education through four pamphlets. Mean age: 57.44 C: obtained self-care education through eight SMS. Mean age: 56.76 D: obtained only usual care of hypertension without any training about self-care management. Mean age: 57.51	To examine whether self-care behaviours could modulate blood pressure levels and also comparing the different training methods of self-care on controlling hypertension.	Patients in group A, B and C were advised to adhere to take medication daily; increase physical activity aimed for 30–45 min of moderate-intensity aerobic activity (such as a brisk walk); most days of the week; follow the dietary approach to stop hypertension (DASH diet) including eat a diet rich in vegetables and reduce dietary sodium to below 1500 mg/day; stop smoking. Group A received this advice through 8 sessions, Group B through 4 pamphlets, and group C through SMS. Duration: 8 months User involvement in development: not stated.	Group D did not obtain any education about self-management of hypertension and they had only usual care of hypertension in the clinics.

#### Alcohol

We identified eight randomised controlled trials which aimed to reduce alcohol intake (Table 6). The alcohol reduction trials included a total of 4782 participants with sample sizes ranging from 18 to 1929. In five trials the intervention was delivered by SMS, in two trials the intervention was delivered by mobile phone application and in one trial the intervention was delivered through interactive voice response.

**Table 6:** Description of trials of health behaviour change interventions: Alcohol consumption.

Study	Study Design, Device, and Media	Participants	Aims	Interventions	Comparators
**Agyapong 2013 [84]**	Parallel group RCT; Country: Ireland; Device: Mobile telephone; Media: SMS	54 patients were randomised. Patients were eligible if they fulfilled the following criteria: a) Age over 18 years and able to provide informed consent. b) Mini Mental State Examination score ≥ 25. c) Patient fulfilled the criteria for both Major Depressive Disorder and Alcohol Dependency Syndrome/Alcohol Abuse and was enrolled on the in-patient dual diagnosis treatment programme in St Patrick's University Hospital d) Did not fulfil the criteria for bipolar affective disorder, psychotic disorder or current poly-substances dependence or abuse e) Patient had a mobile phone, was familiar with text messaging technology, was able to read and be available for follow-up during the study period. Mean age: Intervention = 48y (SD 10.4); control = 49.1y (SD 10.5)	To explore the effects of supportive text messages on mood and abstinence outcomes for patients with depression and co-morbid AUD at 6 months	Starting from the day of discharge from in-patient care, patients in the intervention group received twice daily supportive text messages for three months. The messages were sent by a computer programme at 10.00 and 19.00 h each day and were set up and monitored by the research worker who undertook the randomisation. The intervention lasted 3 months. Participants were followed up at 6 months User involvement in development: not stated–messaged written by research team and two addiction counsellors.	Participants in the control arm did not receive text messages
**Andersson 2015 [85]**	Parallel 5 arm RCT. Country: Sweden. Device: mobile phone. Media: IVR.	1,678 university students in Sweden who reported hazardous drinking in their baseline assessment. Average age 23.2 y (SD 2.9), Female: 41% (not reported by intervention group—paper reports ‘no baseline difference in age or gender’.)	To evaluate a brief automated alcohol intervention designed to reduce heavy episodic drinking, comparing single and repeated IVR delivery, single and repeating WEB (email) delivery, and control group (screening only).	The same intervention content was delivered either by WEB or IVR. The IVR prompts heard by participants were read by a known Swedish television and radio personality whose voice was easy to recognize, the WEB intervention read by participants was written in plain text delivered as a link attached to an email. The interventions were delivered as a single or repeated intervention. Each intervention was brief and involved less than 500 words; personalized information was included in the text shown. The content of the intervention included personalised information on past-month BAC and information on negative consequences if BAC above 0.06%, information on drinks, personalised consumption recommendations, personal goals. Duration: 6 wks. User involvement in development: None.	The control group received no treatment.
**Gajecki 2014 [86]**	Parallel 3 arm RCT. Country: Sweden Device: smartphone Media: Mobile applications.	1929 university students in Sweden with established levels of risky drinking. Promillekoll app intervention: Mean age: 24.6 (SD 4.99) Female: 48.1%. PartyPlanner intervention: Mean age: 24.8 (SD 4.6). Female: 51.7% Control group: Mean age: 24.7 (SD 4.8). Female: 53.5%.	Investigate the effects of 2 smartphone apps with real time eBAC calculations among university students with established levels of risk drinking. Secondly to explore whether there are any gender differences for these 2 applications in terms alcohol outcomes.	Participants were emailed a link to the smartphone app and instructed to use the app for the following 8 weeks. One group received the Promillekoll app which predicts real-time estimated blood alcohol concentration (eBAC) calculation and offers a number of strategies to maintain alcohol consumption at a level that is not harmful. The second group were given access to the PartyPlanner app, which allowed people to simulate a drinking event beforehand and comparing the simulation to real-time events. Duration: 7 wks. User involvement in development: not stated. One app was commercially available, one app was developed by research team.	The control group did not receive access to any intervention or feedback on risky drinking.
**Gustafson 2014 [87]**	Unmasked parallel group RCT involving 3 residential programmes. Country: USA Device: Mobile telephone; Media: Smartphone application	179 participants were randomised. Patients who met the criteria for DSM-IV alcohol dependence upon entering treatment at 3 residential programs. Patients had to be at least 18 years old, willing to be randomized, and able to identify 2 backup contacts people who could provide information about how to reach the patient for one year. Mean age: 38y (SD 10)	To determine whether patients leaving residential treatment for alcohol use disorders with a Smartphone application to support recovery have fewer risky drinking days than control patients.	The A-CHESS group received treatment as usual plus a Smartphone with A-CHESS for the 8-month intervention period and treatment as usual only during the 4-month follow-up. A-CHESS had both static content (e.g., audio-guided relaxation) and interactive features. Duration: 12 months. User involvement in development: Focus groups with 48 participants for needs identification and reactions to components of ACHESS.	The control group received treatment as usual for 12 months
**Haug 2015 [88]**	Parallel group RCT. Country: Switzerland. Device: mobile phone. Media: SMS	50 clients treated for alcohol use disorders from three Swiss outpatient alcohol treatment centres. Average age: Control 50.4 (SD 12.7); Intervention 43.8 (SD 10.7). Female: control 28%, intervention 20%.	To test the feasibility, acceptability and initial effectiveness of a text message-based aftercare treatment programme among alcohol outpatients.	The intervention was primarily based on behavioural self-control techniques (e.g. ‘goal setting’ and ‘self-monitoring’) as well as social support. The intervention included (a) monitoring of self-selected drinking goals at regular intervals, (b) motivational text messages to stick to the self-selected drinking goal and (c) proactive telephone calls from the counsellor for participants that were either not sticking to their drinking goal or in need of support. For a period of 6 months, a computer expert system automatically generated individually tailored text messages for the weekly (Weeks 1–8) or bi-weekly (Weeks 10–26) monitoring of self-selected drinking goals. Based on participant’s response to the monitoring messages, participants would receive a supportive text message or a phone call from a counsellor. Duration: 6 months. User involvement in development: not stated.	Participants in the control group received usual care.
**Mason 2014 [89]**	Parallel group RCT. Country: USA. Device: mobile phone. Media: SMS	18 students enrolled undergraduate psychology courses at a large southeastern university who met criteria of ‘hazardous’ drinking. Mean age: 19.2 y (SD 1.3). Females: 56%. (Not provided by intervention group)	To assess the feasibility and effectiveness of an alcohol counselling intervention delivered via personalised text messages for college students with problem alcohol use.	The intervention group received between four and six text messages daily for 4 days that required brief participant responses during the week following the web-based baseline assessment. Participants in the intervention group could also request booster texts for additional support. Texts were personalized using data collected at baseline. Duration: 1 month. User involvement in development: not stated.	No texts
**Suffoletto 2012 [90]**	RCT with 3 arms. Country: USA Device: Mobile telephone; Media: SMS	109 18–24 year olds were screened for hazardous drinking and 52 screened positive. To be eligible for the trial, participants had to own a personal mobile phone with text messaging features. Those who reported previous treatment for alcohol dependence or current treatment for any psychiatric condition were ineligible. Those who met inclusion criteria were enrolled and randomized. 45 were randomised. Mean age: 21y (SD 1.8)	To determine whether text messaging can be used to assess drinking in young adults and can deliver brief interventions to young adults discharged from the emergency department.	Each week for 12 weeks, assessment group participants received the following text message: ‘‘Pittsburgh Alcohol Research: Please respond ‘‘Yes” within 6 hours to start your weekly questions. DON’T TEXT WHILE DRIVING.” For the Intervention group initial TM-based prompts were identical to the Assessment group. When participants replied they received a tailored message depending on the amount of alcohol they reported to have consumed. Duration: 3 months. User involvement in development: not stated.	Each week for 12 weeks, participants in the Control group received the following text message, ‘‘Pittsburgh Alcohol Research: Look for our email in [X] weeks to complete your final survey,” where [X] was the number of weeks until study completion.
**Suffoletto 2014 [91]**	Parallel three group RCT. Country: USA. Device: mobile phone. Media: SMS	765 young adult emergency department patients who screened positive for past hazardous alcohol use. Mean age: Control: 21.8 (SD 2.1); SMS and feedback group: 22.0 (SD 2.0); SMS only group: 22.0 (SD 2.0). Females: 67%; SMS and feedback group: 65.4%; SMS only group: 63.8%.	To evaluate the efficacy of a 12-week SMS intervention that encourage lower alcohol consumption, specifically binge drinking (≥5 drinks per occasion for men and ≥4 drinks per occasion for women) among young adults.	SA+F participants received a series of welcome text messages within 1 hour of enrolment, describing what to expect during the course of intervention exposure. Each Thursday, for 12 weeks, they were sent a text asking them to report their weekend drinking plans. If they reported anticipating a heavy drinking day, they were then asked whether they were willing to set a low-risk drinking goal (<5 drinks per occasion for men or <4 drinks per occasion for women). Depending on the response to each query, participants were provided with real-time text feedback to either strengthen their low-risk drinking plan or goal, or to promote reflection on their drinking plan or decision not to set a low-risk goal. Then, on Sunday, participants were sent a text asking them to report the most drinks they had during a single occasion during the weekend. Depending on their response, they were provided with text feedback to either support their low-risk drinking behaviour or promote reflection on their binge-drinking behaviour. Participants in the SA group did not receive any pre-weekend text message assessments but received identical text drinking assessments each Sunday for 12 weeks without receiving any alcohol-related feedback. Duration: 3 months User involvement in development: intervention based on that used in Suffoletto et al., 2012, “further developed by a multidisciplinary team of emergency physicians and alcohol treatment specialists using feedback from young adult drinkers.”	No SMS

AUD, alcohol use dependency; SMS, short messaging service; RCT, randomised controlled trial

All of the alcohol reduction trials were conducted in high income countries.

#### Behaviour change techniques

According to our behaviour change technique coding of the studies (Table 7), smoking cessation studies included between 1 and 13 BCTs (median: 8), physical activity/diet studies included between 0 and 9 BCTs (median: 5), the two combined physical activity, diet and smoking trials included 1 and 11 BCTs, and alcohol studies included between 5 and 13 BCTs (median: 8).

**Table 7:** Behaviour change techniques (BCTs) employed in studies.

	Target behaviour					
BCT	Smoking	Alcohol	Physical activity	Diet	Diet and Physical activity	Lifestyle (smoking, diet and physical activity)
1. Provide information about behaviour health link. (IMB)	[20, 22–24, 27, 33, 34, 37]	[85, 91]	[44, 92]		[63, 81]	[82]
2. Provide information on consequences. (TRA, TPB, SCogT, IMB)	[20–26, 28, 32, 34–37]	[85, 86, 90, 91]	[44, 46, 48, 49, 92]		[55, 66, 80, 81]	[82]
3. Provide information about others’ approval. (TRA, TPB, IMB)	[22, 23, 25, 26, 32, 37]				[81]	
4. Prompt intention formation. (TRA, TPB, SCogT, IMB)	[20, 21, 25, 26, 32, 34, 36]	[84–86, 91]	[39, 41, 46, 51]	[53]	[55, 58]	[82]
5. Prompt barrier identification. (SCogT)	[20, 22, 23, 25–27, 32, 34–37]	[87, 90, 91]	[46, 92]	[53]	[58, 66, 69–72, 74, 77, 78, 93]	[82]
6. Provide general encouragement. (SCogT)	[20, 22–24, 27, 33, 34, 36, 37]	[84, 88, 89]	[39, 41, 44]		[55, 58, 61, 62, 66, 68, 72, 81]	[82]
7. Set graded tasks. (SCogT)	[25, 26, 32]	[88]	[39, 92]		[69, 70]	[82]
8. Provide instruction. (SCogT)	[21, 23–28, 32–34, 36, 37]	[84, 85, 87, 89–91]	[39, 41, 44, 46, 92]		[54, 55, 57, 58, 61–63, 65, 66, 68–72, 74, 77, 79–81, 83, 93]	[52, 82]
9. Model or demonstrate the behaviour. (SCogT)	[20, 36]	[91]			[71]	
10. Prompt specific goal setting. (CT)	[21–23, 25–27, 32–34, 36, 37]	[85, 86, 88–91]	[39, 40, 44, 47–49, 51]	[53, 67]	[38, 54, 57–61, 64–66, 68, 70–73, 76–78, 93]	
11. Prompt review of behavioural goals. (CT)	[20–23, 36]		[39, 48]	[53]	[38, 64]	
12. Prompt self-monitoring of behaviour. (CT)	[20–23, 25, 26, 30–32]	[86–88, 90, 91]	[39–41, 44–47, 92]	[67]	[38, 54, 56–59, 61, 62, 64–66, 68–74, 76–78, 80, 93]	[52, 82]
13. Provide feedback on performance. (CT)	[21, 25, 26, 28, 31, 32]	[85–87, 89–91]	[39, 40, 44, 46, 51]	[67]	[38, 54, 56–59, 61, 65, 68–73, 76, 78, 80, 93]	
14. Provide contingent rewards. (OC)	[21–23, 25, 26]	[87, 88, 90, 91]	[44]		[58, 61, 62, 65, 68, 71–74, 93]	
15. Teach to use prompts or cues. (OC)			[49, 51]		[72]	[82]
16. Agree on behavioural contract. (OC)	[31]	[88]				
17. Prompt practice. (OC)	[22, 23]		[48]			
18. Use follow-up prompts	[25, 26, 32, 35]		[48, 49]			
19. Provide opportunities for social comparison. (SCompT)	[33, 36]	[85, 89, 91]	[92]	[67]	[54, 57, 62, 77]	
20. Plan social support or social change. (social support theories)	[21, 25–27, 32, 35, 37]	[84, 87–89]		[67]	[54, 57, 61, 65, 69, 70, 76, 77]	
21. Prompt identification as a role model.						
22. Prompt self-talk.		[90, 91]			[59]	
23. Relapse prevention. (relapse prevention therapy)	[21–23, 25–29, 32–37]	[84, 87–89]			[55, 58, 66, 68, 70, 72, 74]	[82]
24. Stress management (stress theories)	[22, 23, 27, 37]	[84]			[70]	
25. Motivational interviewing		[87, 89, 91]				
26. Time management			[92]			[82]

### Outcomes

#### Smoking cessation

The smoking cessation trials reported up to fifteen outcomes. Primary outcomes were as follows: eight trials reported at least one biochemically verified measure of abstinence from smoking (measured using salivary-cotinine testing and/or exhaled carbon monoxide testing)–outcomes included continuous abstinence, 7-day point prevalence of smoking cessation, and 24-hour point prevalence of smoking cessation. Secondary outcomes included self-reported quitting, self-reported intentions and attempts to quit, self-reported use of nicotine replacement therapy, and cognitive and mediator outcomes such as depression and self-efficacy.

#### Physical activity

The physical activity trials reported up to fifteen outcomes. Nine studies reported objectively measured physical activity outcomes (e.g. step counts, activity time) using pedometers and/or smartphone integrated accelerometers; five reported objectively measured anthropometric outcomes such as BMI, body weight, and waist-to-hip ratio; and four studies reported objectively measured medical outcomes such as systolic blood pressure, diastolic blood pressure, heart rate, peak oxygen uptake, blood sugar control, insulin resistance, high density lipoprotein cholesterol, low density lipoprotein cholesterol, total cholesterol, and triglycerides. Secondary outcomes included self-reported measures of physical activity, body weight, and cognitive and mediator outcomes including quality of life, depression, anxiety, satisfaction, and self-efficacy.

#### Diet

The diet trials reported on up to five outcomes. Two trials reported on anthropometric outcomes such as BMI, waist circumference and body weight; one reported on medical outcomes specifically systolic blood pressure and diastolic blood pressure. Secondary outcomes included self-reported dietary behaviour.

#### Physical activity and diet

The trials targeting physical activity and diet reported up to twenty outcomes. Nineteen studies reported on anthropometric outcomes including body weight, waist circumference, hip circumference, BMI, body fat percentage. Eight studies included measure of medical outcomes including systolic blood pressure, diastolic blood pressure, high density lipoprotein cholesterol, low density lipoprotein cholesterol, total cholesterol, triglycerides, heart rate, incident diabetes, HbA1C, and glucose levels. Secondary outcomes included self-reported measures of physical activity, diet, body weight, and cognitive and mediator outcomes including quality of life, knowledge, depression, self-esteem, stress, anxiety, and self-efficacy.

#### Physical activity, diet and smoking

The physical activity, diet and smoking trials reported up to ten outcomes. Primary outcomes included medical outcomes such as systolic blood pressure, diastolic blood pressure, resting heart rate, high density lipoprotein cholesterol, low density lipoprotein cholesterol, total cholesterol, and triglycerides and biochemically confirmed smoking status. Secondary outcomes were self-report measures of physical activity and dietary intake.

#### Alcohol

The alcohol reduction trials reported up to ten outcomes–all of which relied participants to self-report their alcohol consumption.

### Study quality

#### Smoking cessation

**Fig 2:**
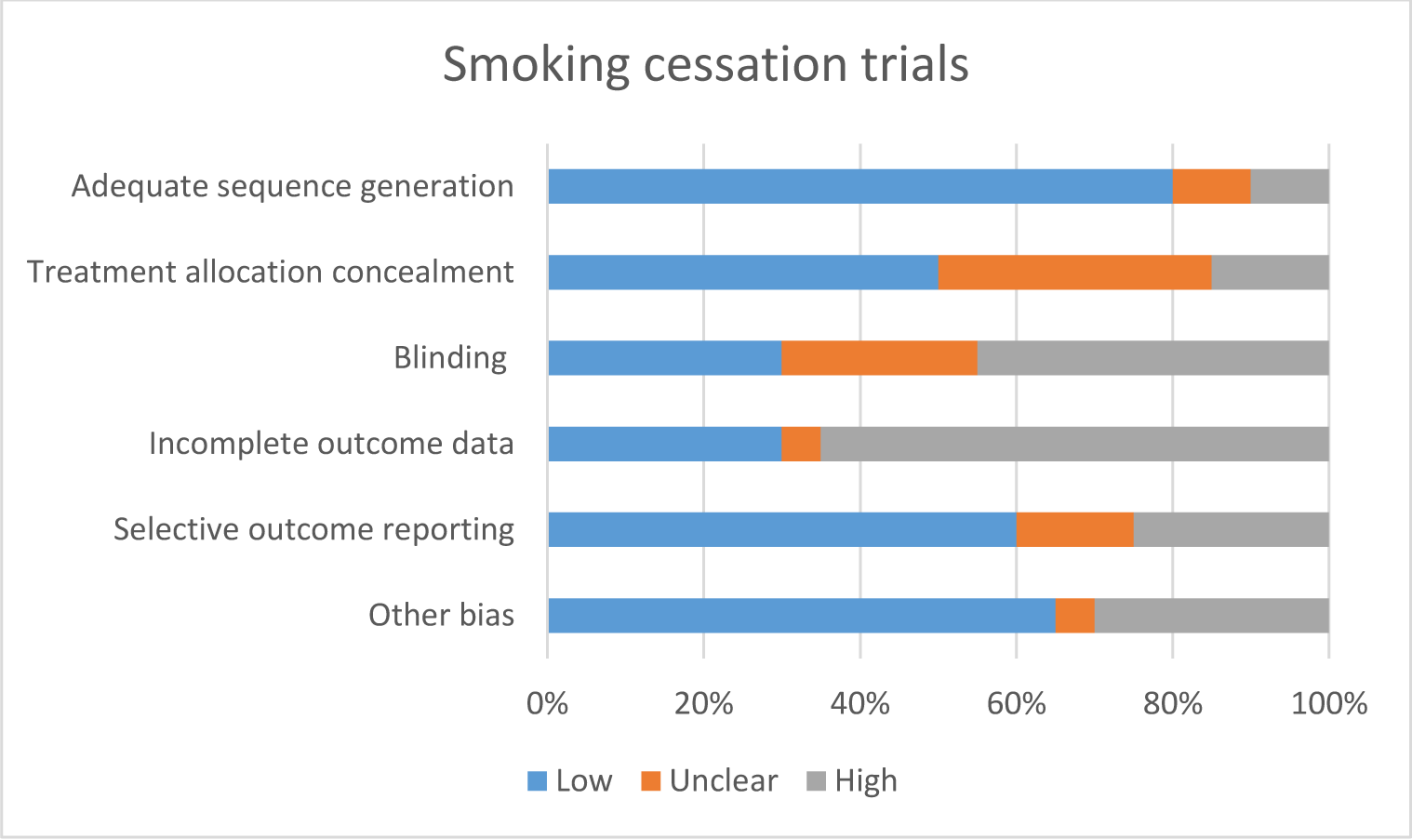
Risk of bias summary–smoking cessation trials.

The assessment of risk of bias for the smoking cessation trials is reported in S1 Table and the risk of bias summary is presented in Fig 2. Two trials targeting smoking cessation were at low risk of bias for all quality criteria [25, 26].

#### Physical activity and diet

**Fig 3:**
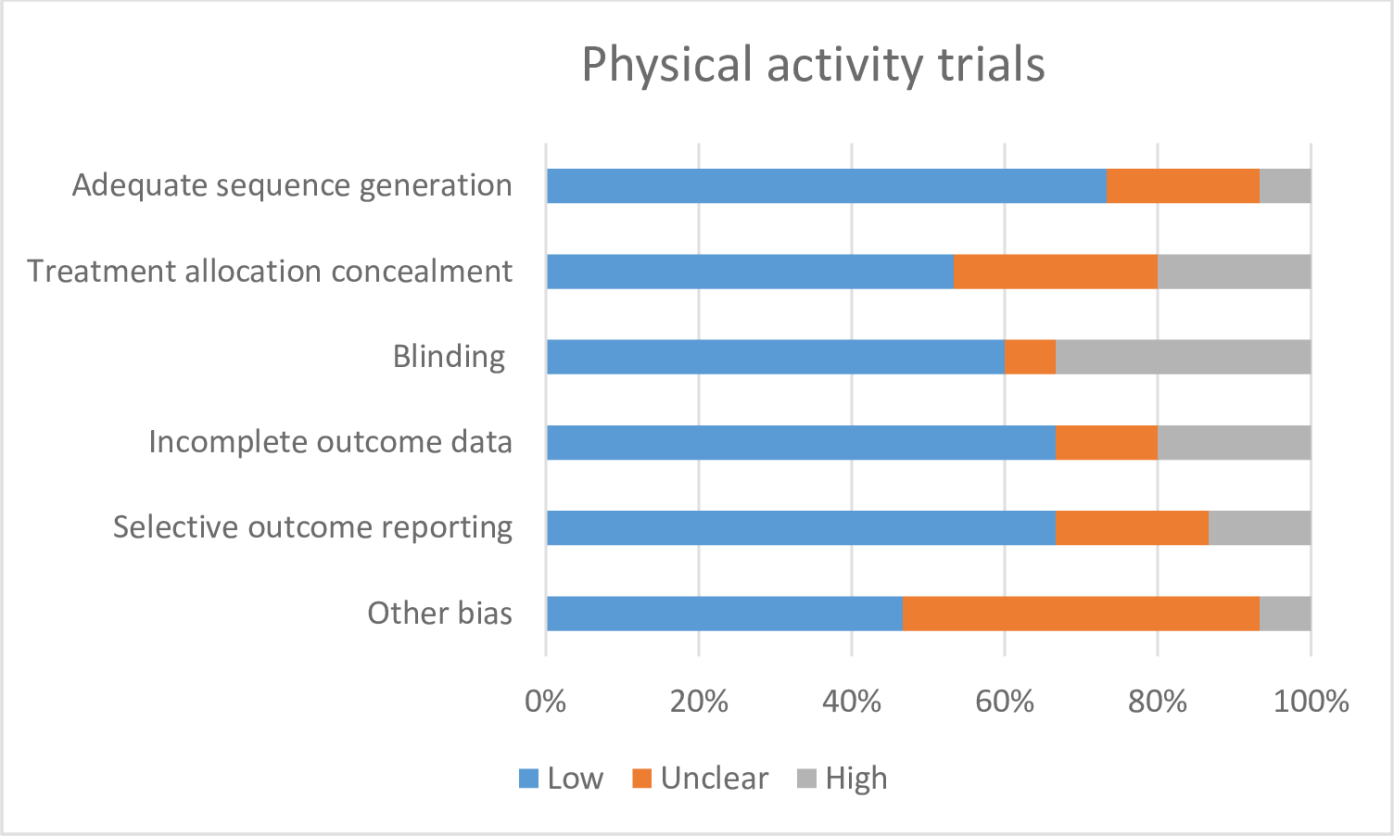
Risk of bias summary–physical activity.

**Fig 4:**
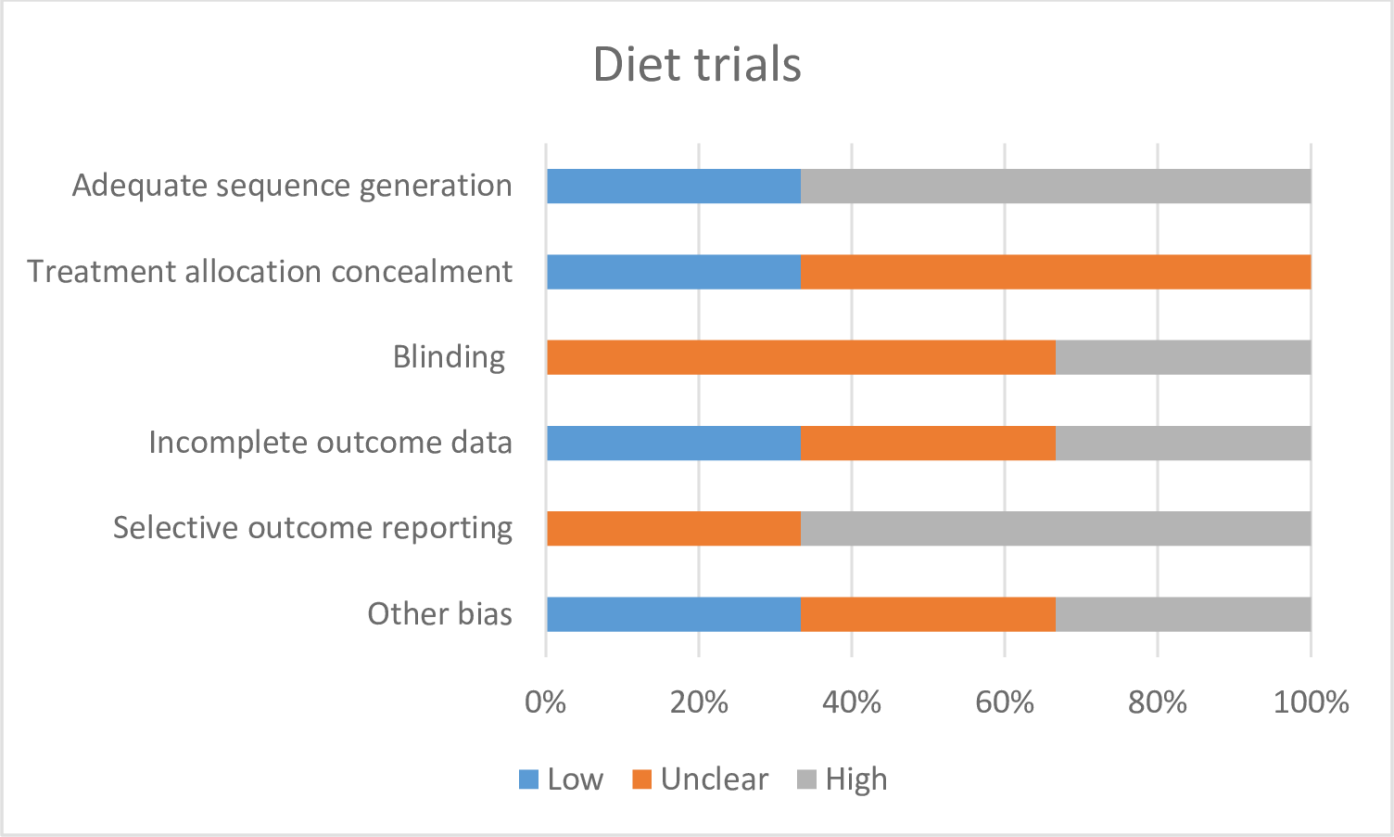
Risk of bias summary–diet.

**Fig 5:**
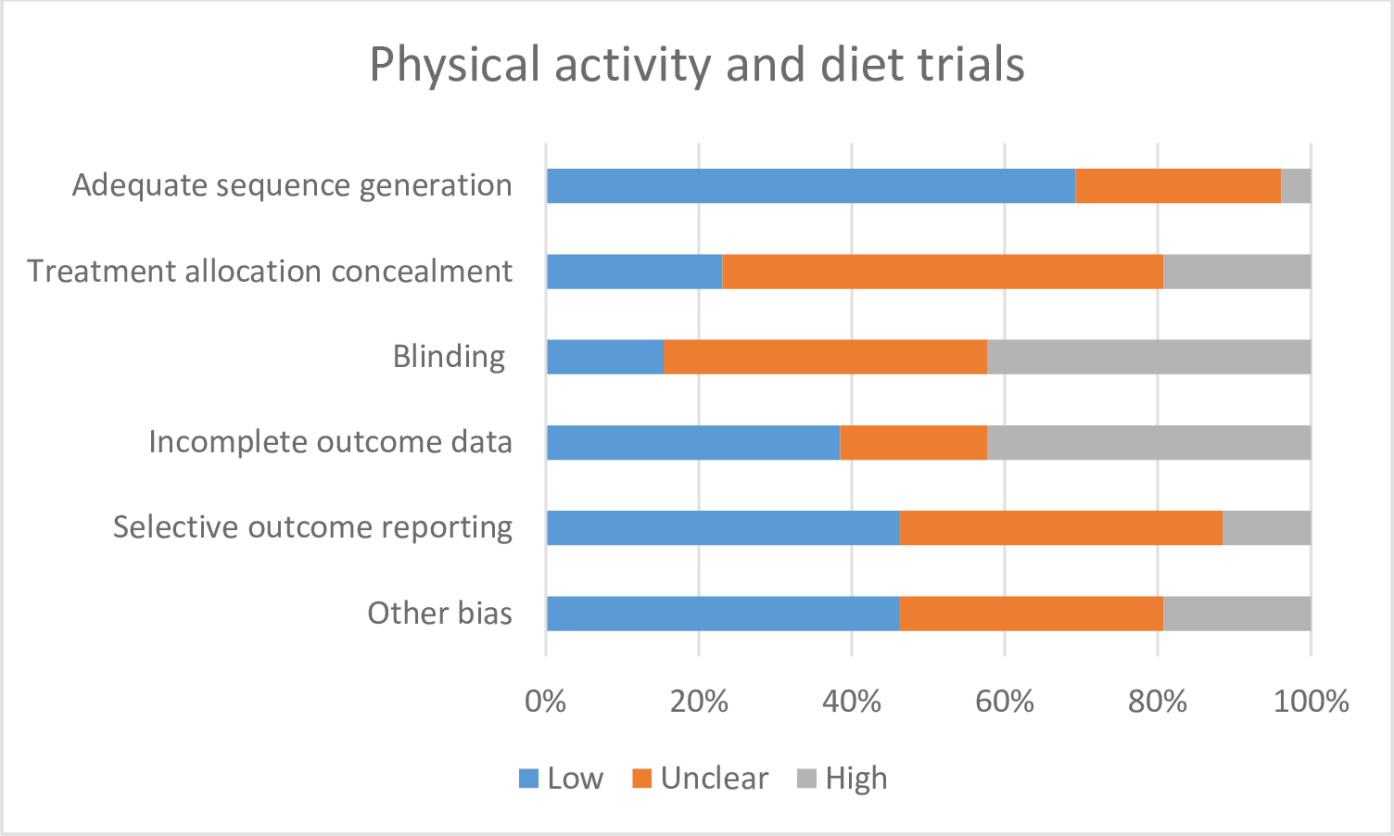
Risk of bias summary–physical activity and diet.

The assessment of risk of bias of the physical activity/diet trials is reported in S1 Table and the risk of bias summary is presented in Figs 3, 4 and 5 (for physical activity only, diet only, and physical activity and diet trials, respectively). Of 26 the trials targeting both physical activity and diet, one was judged to be at low risk of bias for all quality criteria [92]. None of the trials targeting only physical activity or only diet were considered to be at low risk of bias across all quality criteria.

#### Physical activity, diet and smoking

**Fig 6:**
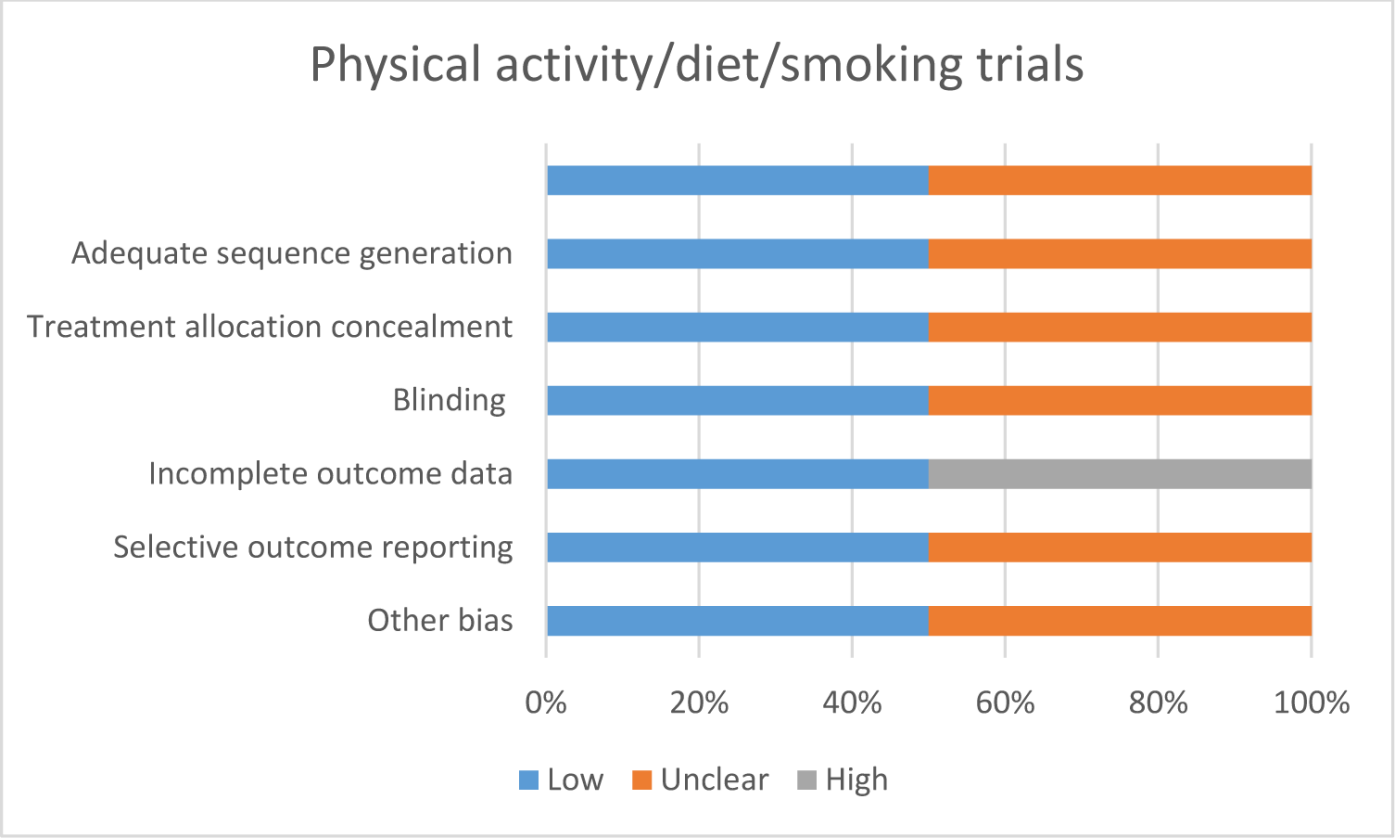
Risk of bias summary–physical activity/diet/smoking.

The assessment of risk of bias of the physical activity, diet and smoking trials is reported in S1 Table and the risk of bias summary is presented in Fig 6. Of the two trials, one was assessed as being at low risk of bias across all quality criteria [82].

#### Alcohol

**Fig 7:**
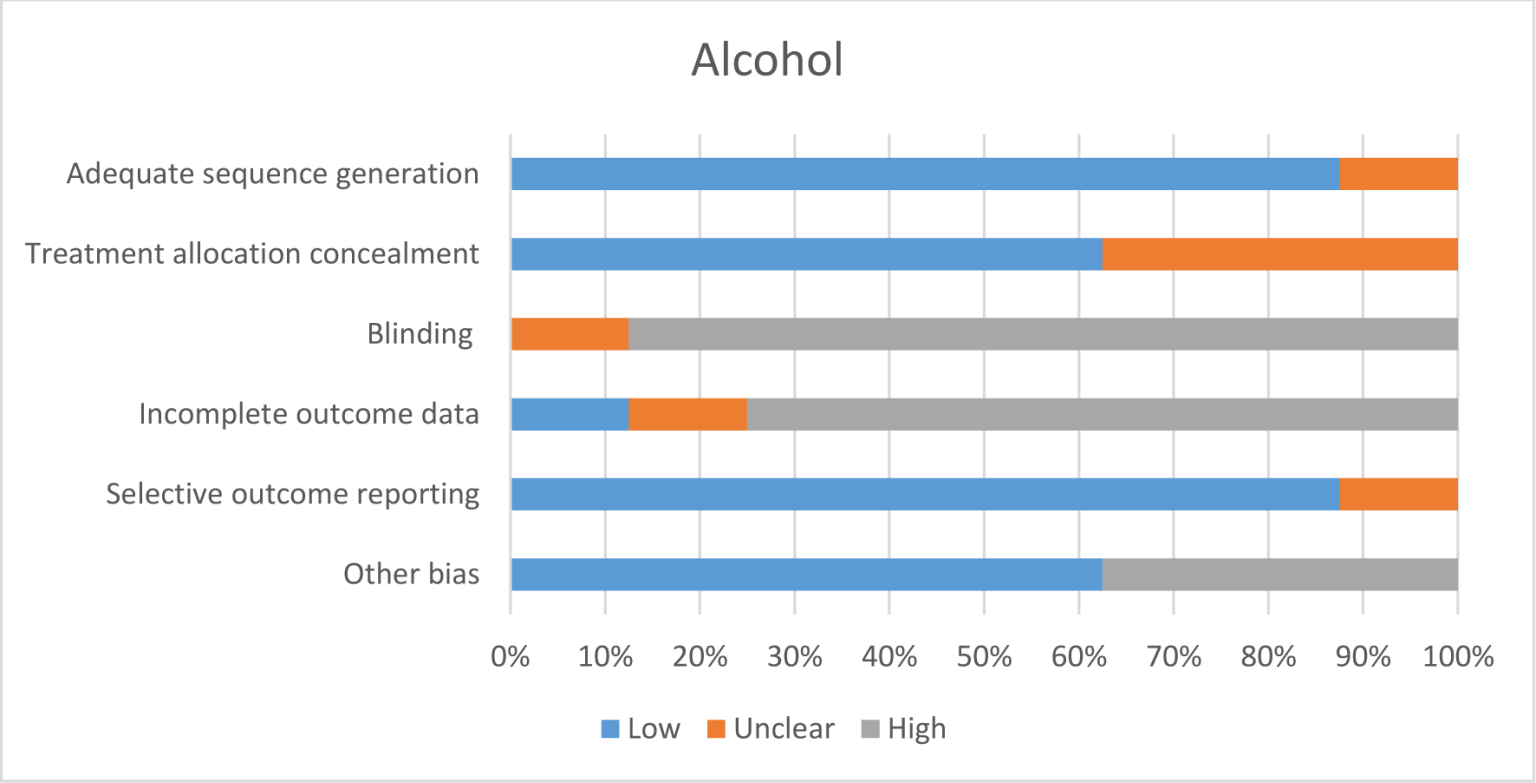
Risk of bias summary–alcohol reduction trials.

The assessment of risk of bias of the physical activity/diet trials is reported in S1 Table and the risk of bias summary is presented in Fig 7. None for the alcohol trials were assessed as being at low risk of bias for all quality criteria.

### Effects

#### Smoking cessation—primary outcomes

**Fig 8:**
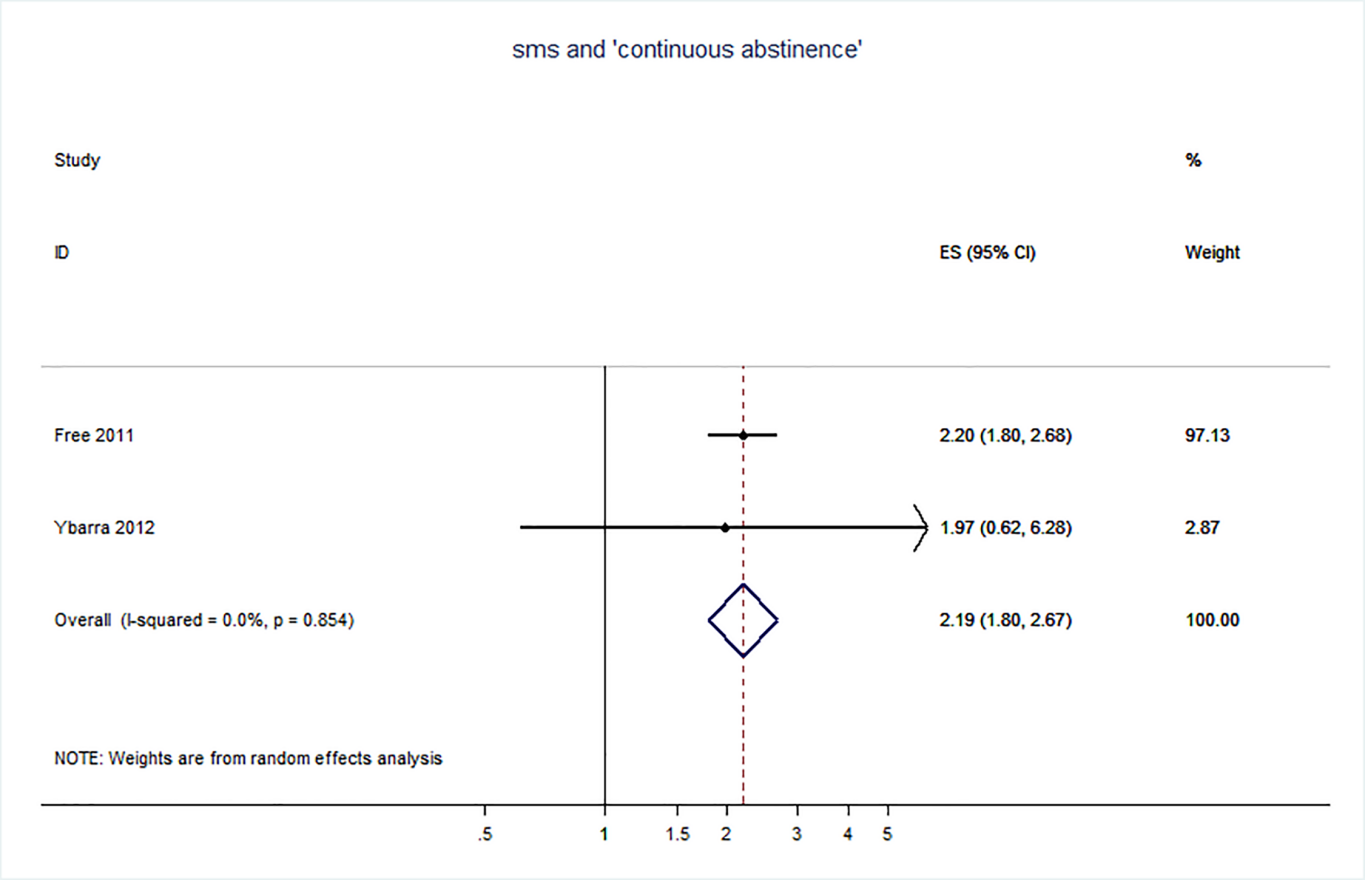
Smoking cessation trials using SMS function–continuous abstinence (biochemically verified).

**Fig 9:**
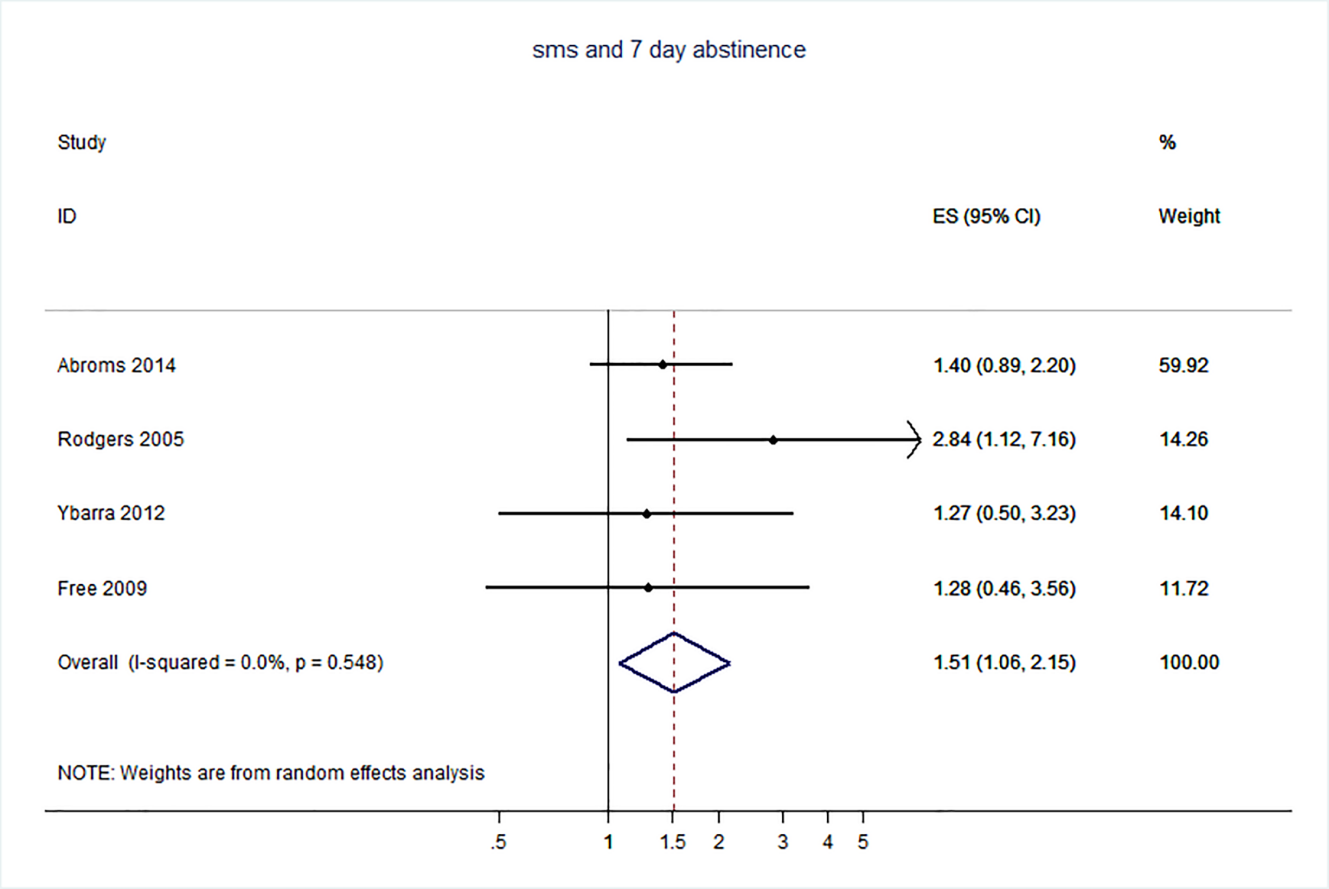
Smoking cessation trials using SMS function– 7 day point prevalence abstinence (biochemically verified).

Interventions delivered by SMS alone. SMS-based smoking cessation interventions providing support for a quit attempt more than doubled biochemically verified continuous smoking abstinence when measured between three and six months [20, 25] (pooled effect estimate relative risk [RR] 2.19 [95% CI 1.80–2.68]) (Fig 8). There was no evidence of between-study heterogeneity (I^2^ = 0%). Pooled analysis showed smoking cessation interventions providing support for a quit attempt significantly increased biochemically verified 7 day point prevalence of smoking cessation [20, 21, 26, 32] (pooled effect estimate RR 1.51 [95% CI 1.06–2.15]), with no evidence of between-study heterogeneity, when measured between three and six months (Fig 9). There was no evidence that SMS-based smoking interventions increased adverse events (car accident in which respondent was the driver pooled RR 1.01 [95% CI 0.71, 1.42], I^2^ = 0.0%; thumb strain pooled RR 1.02 [95% CI 0.83, 1.25], I^2^ = 33.5%) [25, 26, 32].

One trial of an SMS-based intervention promoting smoking cessation showed a statistically significant improvement in biochemically verified smoking cessation at 6 months (time frame of smoking abstinence not defined) [82] (Table 8).

**Table 8:** Effect estimates for primary outcomes (smoking cessation trials).

Clinical Area	Trial	Intervention	Outcome	RR/MD	LCI	UCI
**Smoking**	Abroms 2014 [21]	SMS versus quitting guidebook	Repeated point prevalence abstinence (biochemically verified at 6 months)	2.22	1.16	4.26
	Abroms 2014	SMS versus quitting guidebook	1 week abstinence (biochemically verified) - 6 months	1.4	0.89	2.2
	Chan 2015 [24]	SMS versus self-help booklet	Biochemically validated quit rate 12 months	0.58	0.27	1.25
	Chow 2015 [82]	SMS versus usual care	Current non-smoker at 6 months (self-reported, confirmed with breathalyser)	1.30	1.16	1.45
	Free 2011 [25]	5 SMS/day post quit date versus fortnightly generic SMS	Continuous abstinence (biochemically verified) 6 months	2.20	1.80	2.68
	Free 2009 [26]	5 SMS/day post quit date versus fortnightly generic SMS	Point prevalence smoking cessation (no smoking in last wk, self-reported and biochemically confirmed)– 6 months	1.28	0.46	3.56
	Pollak 2013 [31]	SMS versus SMS using supported gradual reduction	7 day point prevalence abstinence– 6 wk	1.88	0.19	18.60
	Rodgers 2005 [32]	5 SMS/day versus 1 SMS/fortnight	7 day point prevalence smoking cessation—6 wk	2.84	1.12	7.16
	Vidrine 2006 [35]	Counseling sessions on mobile phone versus usual care	7 day point prevalence abstinence—3 months	2.74	0.78	9.55
	Vidrine 2006	Counseling sessions on mobile phone versus usual care	24-h point prevalence abstinence at 3 months follow-up–biochemical)	3.59	1.30	9.94
	Ybarra 2012 [20]	SMS versus no SMS	7 day point prevalence abstinence– 4 wk	1.27	0.50	3.23
	Ybarra 2012	SMS versus no SMS	Continuous abstinence (biochemically verified)– 3 months	1.97	0.62	6.28

Interventions delivered by other or mixed mobile technology media. A trial of mobile phone-based counselling sessions demonstrated a statistically significant improvement in biochemically verified 24-hour point prevalence abstinence at 3 months, but not for 7-day point prevalence at 3 months [35] (Table 8).

#### Smoking cessation—secondary outcomes

Fourteen of the 63 self-reported smoking outcomes showed statistically significant benefits and none showed statistically significant harms. There were eleven studies reporting outcomes relating to cognitive mediators of smoking behaviour change—none showed statistically significant benefits or harms. One study compared a smoking cessation intervention delivered by mobile application with one delivered by text message and found the text messaging arm to have statistically significantly higher self-reported quitting rates, although those in the application arm were more likely to have set a quit smoking date [23] (S2 Table).

#### Physical activity—primary outcomes

**Fig 10:**
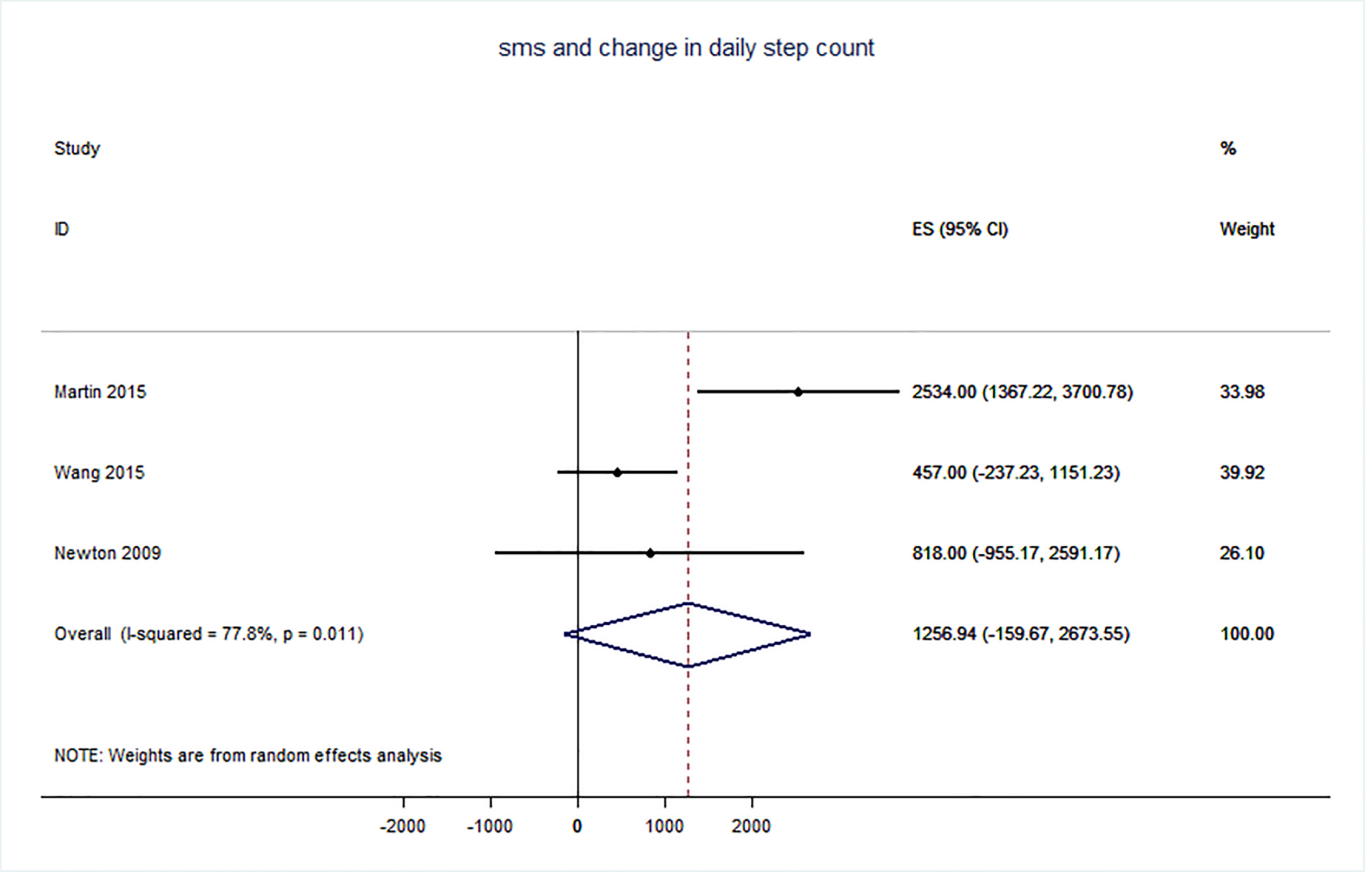
Physical activity interventions using SMS function–change in daily step count.

Interventions delivered by SMS alone: physical activity outcomes. Pooled analysis of three trials examining the effect of SMS-based interventions on physical activity showed a borderline statistically significant increase in objectively measured physical activity [44, 45, 51] (change in steps per day pooled MD 1256.9 [95% CI -159.7 to 2673.6, p-value = 0.081], with evidence of substantial between study heterogeneity (I^2^ = 77.8%) (Fig 10). The outcomes were measured between 4 and 12 weeks. These trials reported 4 other objectively measured physical activity outcomes. Of these, 2 were not in a positive direction and 2 showed statistically significant benefits [44] (Table 9). One additional trial of an SMS-based intervention demonstrated a statistically significant effect on end-line number of steps per day measured at 6 weeks (MD 1750.8 [95% CI 157.4 to 3344.2]) [41].

**Table 9:** Effect estimates for primary outcomes (physical activity trials).

Clinical Area	Trial	Intervention	Outcome	RR/MD	LCI	UCI
**Physical activity**	Cadmus-Bertam 2015 [38]	Fitbit versus standard pedometer	Minutes per week of PA moderate and vigorous—change at 16 weeks	49	-7.67	105.67
	Cadmus-Bertam 2015	Fitbit versus standard pedometer	Light intensity PA minutes per week—change at 16 weeks	19	-98.79	136.79
	Cadmus-Bertam 2015	Fitbit versus standard pedometer	Average steps per day—change at 16 weeks	427	-564.16	1418.16
	Cadmus-Bertam 2015	Fitbit versus standard pedometer	body weight (kg)—change at 16 weeks	-0.31	-1.60	0.98
	Direito 2015 [39]	Zombies run app versus no app	Time to complete 1-mile walk/run (sec) -follow-up 8 wks	-28.36	-66.54	9.82
	Direito 2015	Zombies run app versus no app	Average daily valid use (min) -follow-up 8 wks	-21.72	-84	40.56
	Direito 2015	Zombies run app versus no app	Average daily activity counts (counts/min) -follow-up 8 wks	17.74	-63	98.55
	Direito 2015	Zombies run app versus no app	Average daily time spent in sedentary activities (min) -follow-up 8 wks	-10.94	-69.83	48
	Direito 2015	Zombies run app versus no app	Average daily time spent in light PA (min) -follow-up 8 wks	-10.54	-53.96	32.88
	Direito 2015	Zombies run app versus no app	Average daily time spent in moderate PA (min) -follow-up 8 wks	1.42	-7.96	10.81
	Direito 2015	Zombies run app versus no app	Average daily time spent in vigorous PA (min) -follow-up 8 wks	1.26	-3.82	6.33
	Direito 2015	Zombies run app versus no app	Average daily time spent in MVPA (min) -follow-up 8 wks	1.74	-11.45	14.93
	Direito 2015	Get running non immersive app versus no app	Time to complete 1-mile walk/run (sec) -follow-up 8 wks	-24.67	-63.51	14.18
	Direito 2015	Get running non immersive app versus no app	Average daily valid use (min) -follow-up 8 wks	13.71	-49.56	76.99
	Direito 2015	Get running non immersive app versus no app	Average daily activity counts (counts/min) -follow-up 8 wks	0.90	-85.40	87.22
	Direito 2015	Get running non immersive app versus no app	Average daily time spent in sedentary activities (min) -follow-up 8 wks	3.95	-56.26	64.16
	Direito 2015	Get running non immersive app versus no app	Average daily time spent in light PA (min) -follow-up 8 wks	4.12	-39.94	48.17
	Direito 2015	Get running non immersive app versus no app	Average daily time spent in moderate PA (min) -follow-up 8 wks	-1.71	-11.51	8.10
	Direito 2015	Get running non immersive app versus no app	Average daily time spent in vigorous PA (min) -follow-up 8 wks	0.52	-4.79	5.83
	Direito 2015	Get running non immersive app versus no app	Average daily time spent in MVPA (min) -follow-up 8 wks	-1.8	-16	12.36
	Glynn 2014[40]	Smartphone app versus no app	Change in body mass index (kg/m2) at 8 wks	-0.18	-0.52	0.16
	Glynn 2014	Smartphone app versus no app	Change in weight (kg) at 8 wks	-0.17	-1.11	0.77
	Glynn 2014	Smartphone app versus no app	Physical activity—walking (change in steps per day) at 8 wks	2017	271.52	3762.48
	Glynn 2014	Smartphone app versus no app	Change in systolic blood pressure (mmHg) at 8 wks	-4.4	-9.81	1.01
	Glynn 2014	Smartphone app versus no app	Change in diastolic blood pressure (mmHg) at 8wks	-1.78	-5.72	2.16
	Glynn 2014	Smartphone app versus no app	Change in resting heart rate (beats per minute) at 8 wks	2.6	-1.47	6.67
	Kim 2013 [41]	Motivational SMS versus no SMS	Physical activity—walking (steps per day) - 6 wks	1750.78	157.35	3344.22
	Liu 2008 [42]	Endurance exercises accompanied by music on mobile phone versus booklet and DVD	Post-exercise breathlessness—Borg scale score– 12 months	−0.70	−0.81	−0.59
	Maddison 2014 [43]	SMS plus usual care versus usual care	Peak oxygen uptake (PVO_3_) - 12 wks	-0.2	-1.1	0.7
	Martin 2015 [44]	SMS and unblinded accelerometer versus no SMS and unblinded accelerometer	Step count (per day) change—pedometer (weeks 4–5)	2534	1367.22	3700.77
	Martin 2015	SMS and unblinded accelerometer versus no SMS and unblinded accelerometer	Activity time change (min/day)—pedometer (weeks 4–5)	21	8.14	33.86
	Martin 2015	SMS and unblinded accelerometer versus no SMS and unblinded accelerometer	Aerobic time change (min/day)—pedometer (weeks 4–5)	14	7.34	20.66
	Martin 2015	SMS and unblinded accelerometer versus blinded accelerometer	Step count (per day) change—pedometer (weeks 4–5)	3376	2008.71	4743.29
	Martin 2015	SMS and unblinded accelerometer versus blinded accelerometer	Activity time change (min/day)—pedometer (weeks 4–5)	29	14.07	43.93
	Martin 2015	SMS and unblinded accelerometer versus blinded accelerometer	Aerobic time change (min/day)—pedometer (weeks 4–5)	16	8.72	23.28
	Martin 2015	No texts and unblinded accelerometer versus blinded accelerometer	Step count (per day) change—pedometer (weeks 4–5)	842	-507.14	2191.14
	Martin 2015	No texts and unblinded accelerometer versus blinded accelerometer	Activity time change (min/day)—pedometer (weeks 4–5)	8	-6.014	22.01
	Martin 2015	No texts and unblinded accelerometer versus blinded accelerometer	Aerobic time change (min/day)—pedometer (weeks 4–5)	2	-4.27	8.27
	Newton 2009 [45]	Motivational SMS versus standard care	Physical activity—walking (steps per day)–change– 12 wks	818.00	-955.17	2591.17
	Newton 2009	Motivational SMS versus standard care	Body mass index (kg/m^2^)–change– 12 wks	−0.01	−0.14	0.12
	Newton 2009	Motivational SMS versus standard care	Systolic blood pressure—change (mmHg)– 12 wks	2.10	-9.08	13.28
	Newton 2009	Motivational SMS versus standard care	Diastolic blood pressure—change (mmHg)– 12 wks	1.30	-6.08	8.68
	Newton 2009	Motivational SMS versus standard care	Blood sugar control—change (% HbA1C)– 12 wks	0.37	-0.22	0.96
	Newton 2009	Motivational SMS versus standard care	Insulin intake—change (units per kg)– 12 wks	0.00	−0.02	0.16
	Petrella 2014 [47]	Exercise prescription plus blackberry, blood pressure measure, glucometer, pedometer versus exercise prescription only	Systolic blood pressure (mmHg)—change at 12 weeks	-5.68	-10.86	-0.5
	Petrella 2014	Exercise prescription plus blackberry, blood pressure measure, glucometer, pedometer versus exercise prescription only	Diastolic blood pressure (mmHg)—change at 12 weeks	-2.55	-5.24	0.13
	Petrella 2014	Exercise prescription plus blackberry, blood pressure measure, glucometer, pedometer versus exercise prescription only	Waist circumference (cm)—change at 12 weeks	-0.36	-2.18	1.46
	Petrella 2014	Exercise prescription plus blackberry, blood pressure measure, glucometer, pedometer versus exercise prescription only	Fasting glucose (mmol/mol)—change at 12 weeks	-0.060	-0.24	0.11
	Petrella 2014	Exercise prescription plus blackberry, blood pressure measure, glucometer, pedometer versus exercise prescription only	Blood sugar control (HbA1c %)—change at 12 weeks	0.02	-0.07	0.10
	Petrella 2014	Exercise prescription plus blackberry, blood pressure measure, glucometer, pedometer versus exercise prescription only	Blood sugar control (HbA1c mmol/mol)—change at 12 weeks	0.2	-0.80	1.1
	Petrella 2014	Exercise prescription plus blackberry, blood pressure measure, glucometer, pedometer versus exercise prescription only	Homeostasis model for Insulin resistance—change at 12 weeks	0.12	-0.30	0.53
	Petrella 2014	Exercise prescription plus blackberry, blood pressure measure, glucometer, pedometer versus exercise prescription only	High density lipoprotein cholesterol (HDL) mmol/L—change at 12 weeks	0.04	-0.03	0.10
	Petrella 2014	Exercise prescription plus blackberry, blood pressure measure, glucometer, pedometer versus exercise prescription only	Low density lipoprotein cholesterol (LDL) mmol/L—change at 12 weeks	-0.080	-0.31	0.15
	Petrella 2014	Exercise prescription plus blackberry, blood pressure measure, glucometer, pedometer versus exercise prescription only	Total cholesterol mmol/L—change at 12 weeks	-0.05	-0.30	0.21
	Petrella 2014	Exercise prescription plus blackberry, blood pressure measure, glucometer, pedometer versus exercise prescription only	Triglycerides mmol/L—change at 12 weeks	-0.040	-0.2	0.13
	Petrella 2014	Exercise prescription plus blackberry, blood pressure measure, glucometer, pedometer versus exercise prescription only	High sensitivity C-reactive protein mg/L—change at 12 weeks	-0.16	-0.84	0.53
	Prestwich 2010 [49]	SMS goal reminder versus no SMS	Waist-to-hip ratio– 4 wks	0.00	−0.03	0.03
	Prestwich 2010	SMS plan reminder versus no SMS	Waist-to-hip ratio– 4 wks	−0.01	−0.03	0.02
	Prestwich 2010	SMS goal reminder versus no SMS	Weight (kg)– 4 wks	1.19	−4.47	6.85
	Van der weegen 2015 [78]	Activity monitor, app, self-management support program versus self-management support program only	Minutes of PA moderate and vigorous > = 3 METs at 9 months	9.41	3.7	15.11
	Wang 2015 [51]	SMS plus Fitbit One versus Fitbit One only	Steps (n/day) -change at 6 wks	457	-237.23	1151.23
	Wang 2015	SMS plus Fitbit One versus Fitbit One only	Moderate to vigorous physical activity (minutes per week) change at 6 wks	-5.40	-11.52	0.72
	Wang 2015	SMS plus Fitbit One versus Fitbit One only	All intensity physical activity (minutes per week)—change at 6 wks	-9.40	-21.46	2.66

Interventions delivered by SMS alone: Anthropometric and bio-medical outcomes. Two trials of SMS interventions targeting physical activity reported 5 anthropometric outcome measures (waist-to-hip ratio, body weight, BMI), none of which showed benefits [45] [49]. Two of the physical activity trials included 5 bio-medical outcomes (such as blood pressure, blood sugar control, post-exercise breathlessness)–none of which demonstrated a benefit [45, 92] (Table 9).

Interventions delivered by other or mixed mobile technology media: physical activity outcomes. Two trials of mobile-application interventions reported 17 objectively measured physical activity outcomes–of these, one showed statistically significant improvement in change in number of steps per day measured at 8 weeks [40] (MD 2017 [95% CI 271.5 to 3762.5]). Five of the other 16 outcomes were in a positive direction but not statistically significant at 8 weeks [39]. A trial of a fit-bit versus standard pedometer reported increases in physical activity which were not statistically significant using 3 objective measures at 16 weeks [38]. A trial of an SMS based intervention plus an unblinded accelerometer reported statistically significant increases in 3 objective measures of physical activity at 4 weeks [44] (Table 9).

Interventions delivered by other or mixed mobile technology media: anthropometric and bio-medical outcomes. A trial of an app-based intervention found no effects on weight loss or BMI at 8 weeks [40], and a trial of a fit-bit intervention found no effect on weight loss at 16 weeks [38].

A trial of an app found no effect on three medical outcomes (change in systolic/diastolic blood pressure or change in resting heart rate) at 8 weeks [40]. A trial involving multiple monitoring devices connected to a blackberry phone found a small but statistically significant effect on change in systolic blood pressure, but no effect on the other eleven medical outcomes reported at 12 weeks [47].

#### Diet–primary outcomes

Interventions delivered by other or mixed mobile technology media: anthropometric and bio-medical outcomes. One trial in which personal email advice was sent by mobile phone in response to self-monitoring of daily salt excretion observed no statistically significant effect of the intervention on blood pressure, BMI, waist circumference or body weight [52]. One trial assessing the efficacy of a diet tracking application versus instructing participants to track their dietary intake on their phone’s memo function showed no statistically significant effect on weight loss [54] (Table 10).

**Table 10:** Effect estimates for primary outcomes (diet trials).

Clinical Area	Trial	Intervention	Outcome	RR/MD	LCI	UCI
**Diet**	Morikawa 2011 [52]	Smartphone Na excretion monitoring versus usual care	Systolic blood pressure (mmHg)– 4 wks	-2.80	-10.36	4.76
	Morikawa 2011	Smartphone Na excretion monitoring versus usual care	Diastolic blood pressure (mmHg)– 4 wks	-4.00	-9.35	1.35
	Morikawa 2011	Smartphone Na excretion monitoring versus usual care	BMI (kg/m^2^)– 4 wks	0.10	-1.84	2.04
	Morikawa 2011	Smartphone Na excretion monitoring versus usual care	Waist circumference (cm)– 4 wks	-0.90	-5.36	3.56
	Morikawa 2011	Smartphone Na excretion monitoring versus usual care	Body weight (kg)– 4 wks	0.20	-5.01	5.41
	Wharton 2014 [54]	Diet tracking app versus tracking on memo phone function	Change in weight (lb) at 8 wks	3.00	-0.37	6.37
	Wharton 2014	Diet tracking app versus tracking with pencil/paper	Change in weight (lb) at 8 wks	0.90	-2.16	3.96
	Wharton 2014	Tracking on memo phone function versus tracking with pencil/paper	Change in weight (lb) at 8 wks	-2.10	-5.71	1.51

#### Diet and physical activity—primary outcomes

**Fig 11:**
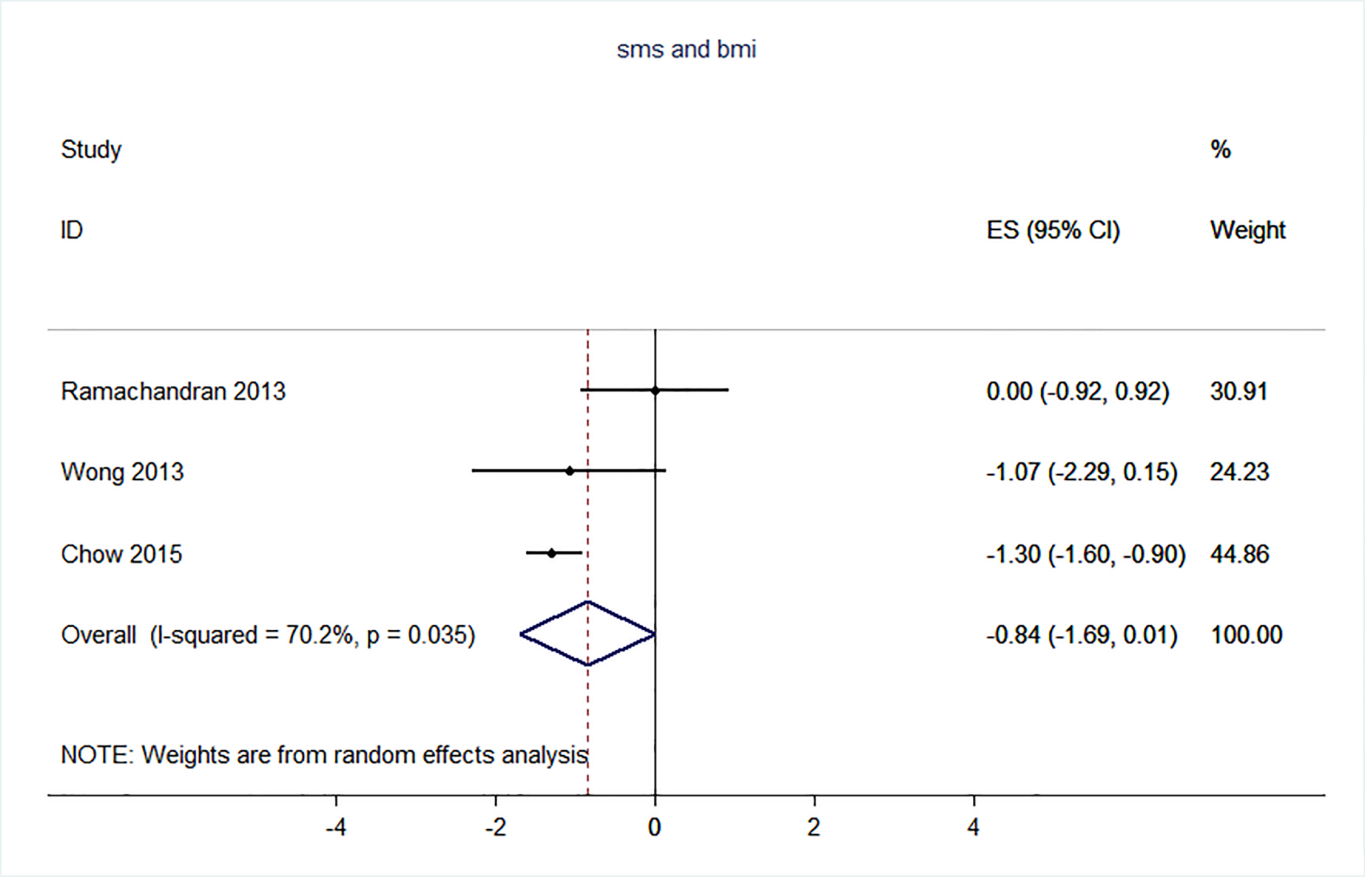
Physical activity and diet interventions using SMS function–BMI.

Interventions delivered by SMS alone: anthropometric outcomes. Random effects meta-analysis showed a borderline statistically significant effect of SMS-based interventions on BMI scores measured in three trials between 6 and 24 months[55, 81, 82] (pooled effect estimate mean difference [MD] -0.84 [95% CI -1.69, 0.01] p = 0.052), however there was evidence of substantial between-study heterogeneity (I^2^ = 70.2%) (Fig 11). In the two other SMS based intervention trials, one observed no effect on change in BMI at 6 months [93]. The other observed a small but statistically significant reduction in BMI when measured in percentiles (MD -0.10 [95% CI -0.13, -0.07]) but no effect when BMI was measured using Z-scores at 12 months (MD 0.00 [95% CI -0.30 to 0.30]) [71].

**Fig 12:**
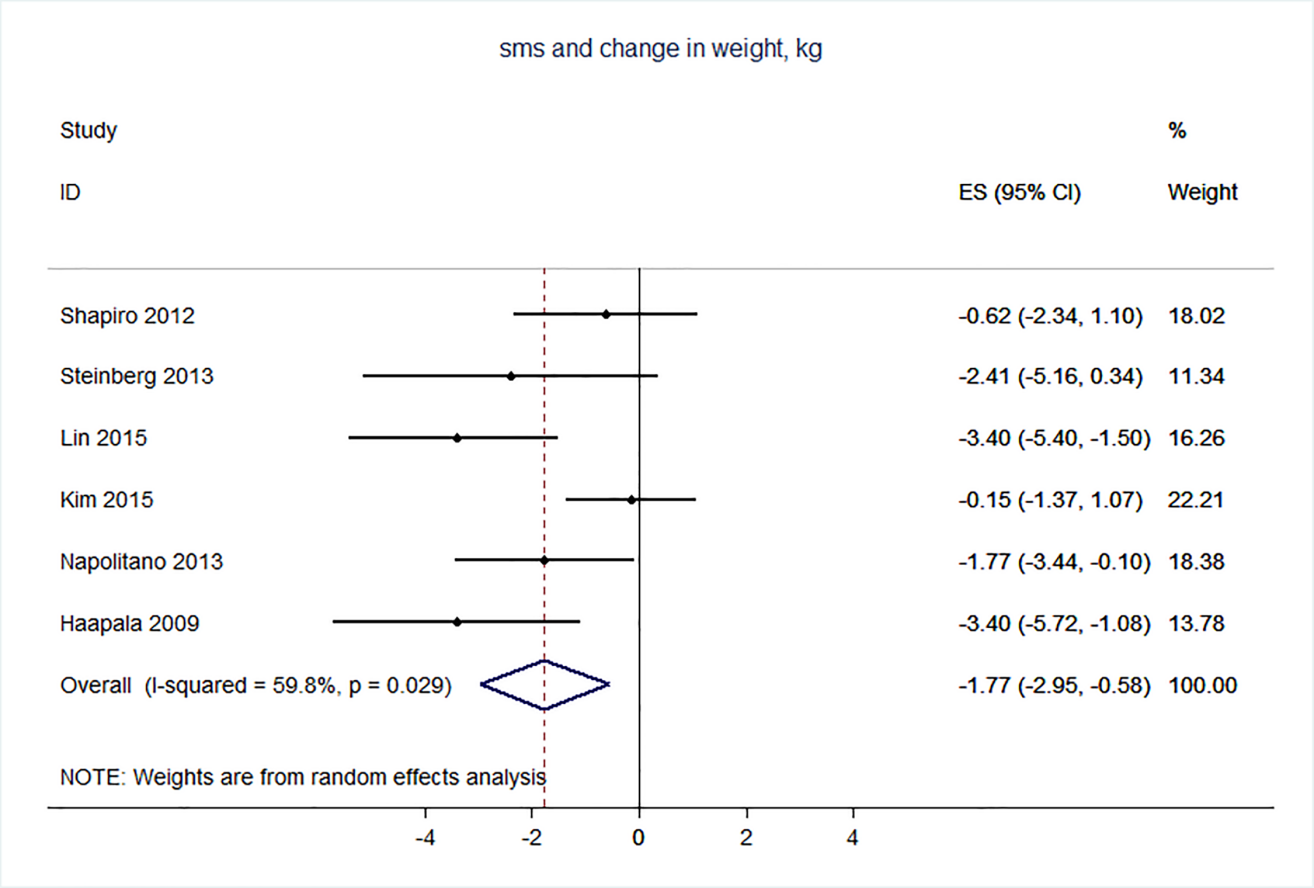
Physical activity and diet interventions using SMS function–change in weight (kg).

**Fig 13:**
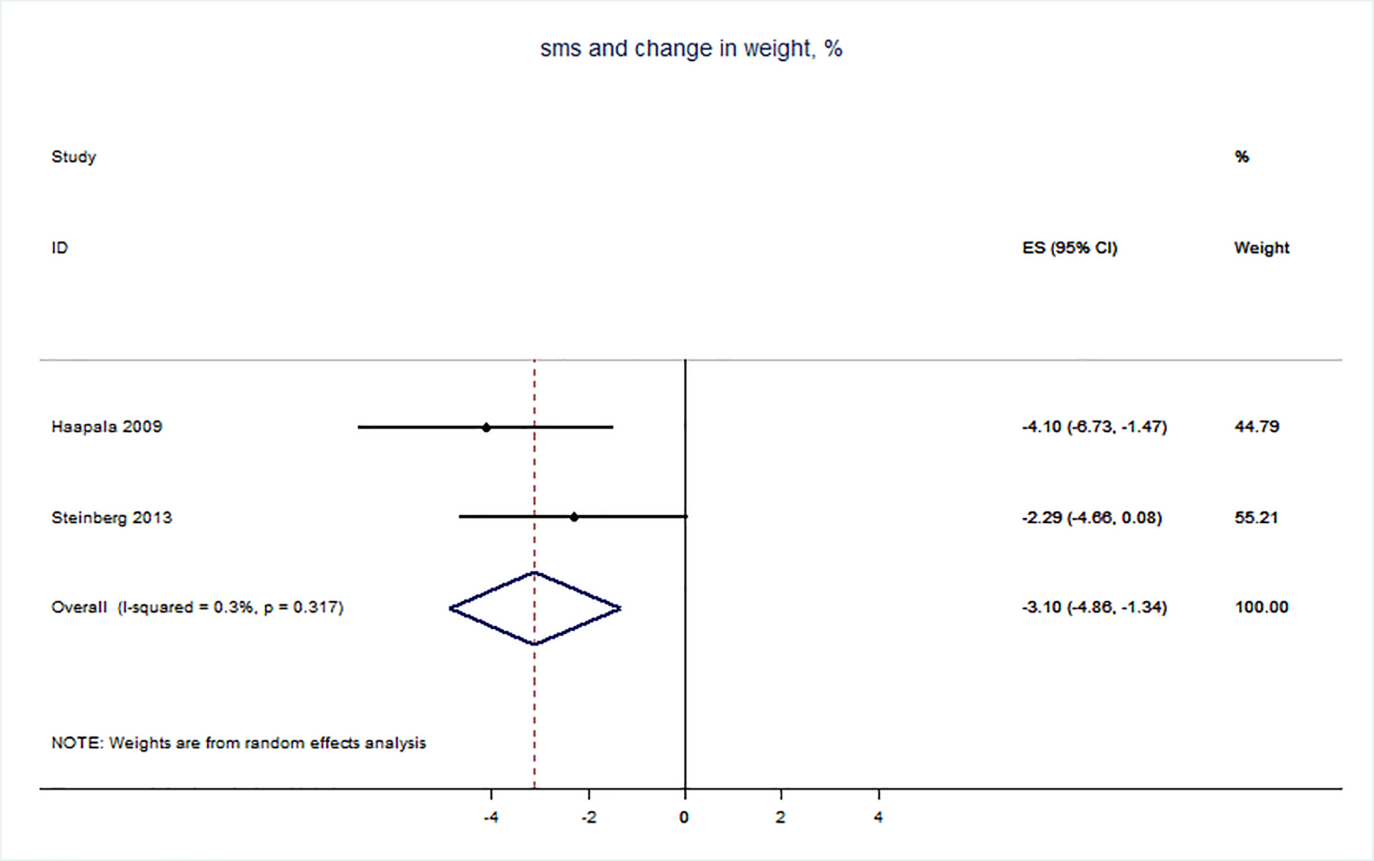
Physical activity and diet interventions using SMS function–change in weight (%).

**Fig 14:**
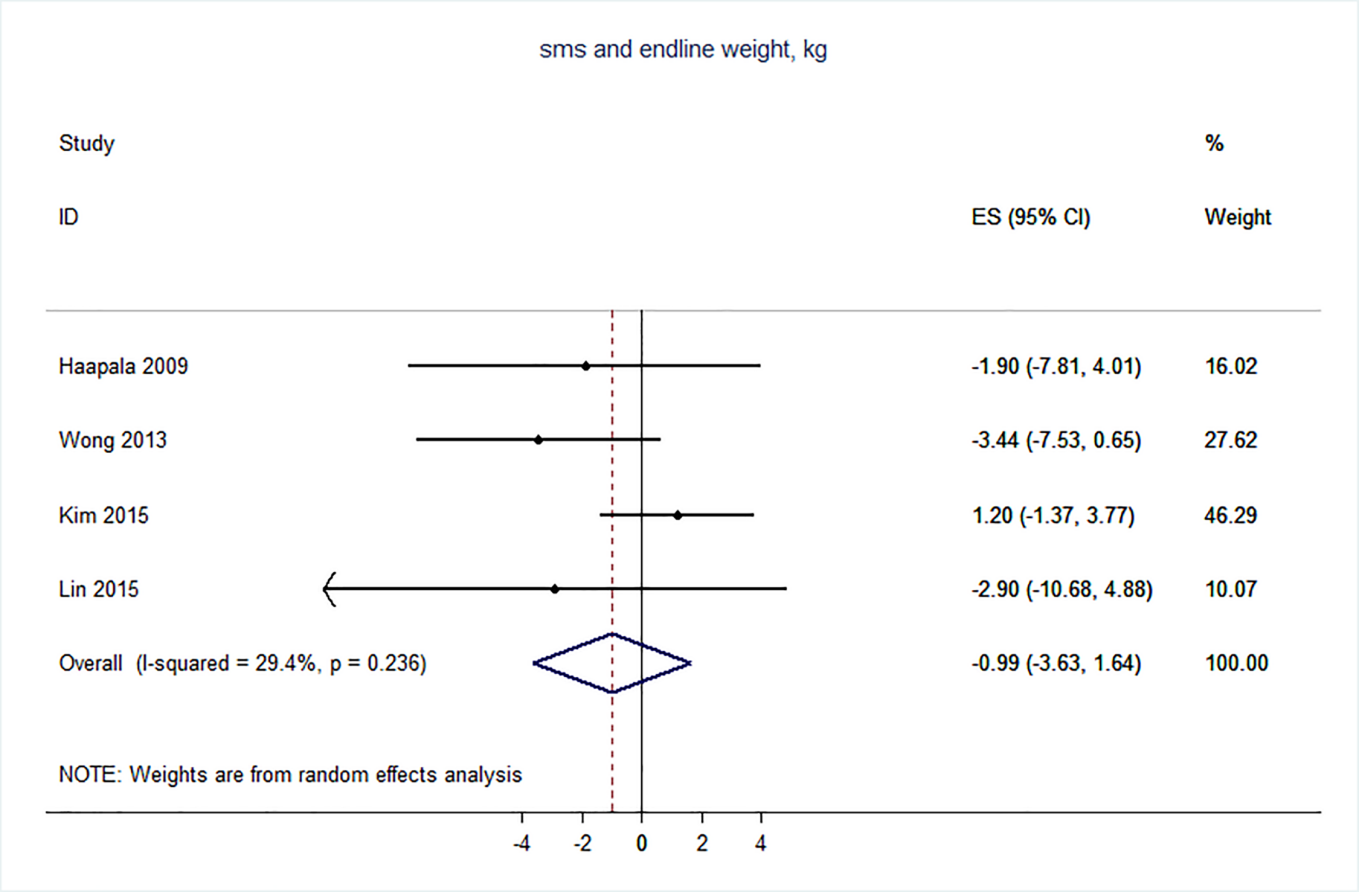
Physical activity and diet interventions using SMS function–weight endpoint (kg).

Six studies examined the effect of SMS based interventions on objectively measured change in body weight. Pooled analyses indicated effectiveness in promoting greater weight loss measured between 8 weeks and 12 months [64, 66, 68, 70, 72, 93] (pooled MD -1.77kg [95% CI -2.95, -0.58] p = 0.004) (Fig 12). There was evidence of substantial between-study heterogeneity (I^2^ = 59.8%). The effect of SMS-based interventions was also demonstrated in pooled analyses for the outcome of percentage change in weight measured between 6 and 12 months (pooled MD -3.10% [95% CI -4.86, -1.34], p = 0.001 I^2^ = 0.3%) (Fig 13) [64, 93]. However, no statistically significant difference was observed in pooled analyses of SMS-based interventions when using end-point weight as the outcome measured between 6 and 12 months (MD -0.99 [95% CI -3.63, 1.64], p = 0.461, I^2^ = 29.4%) [64, 66, 68, 81] (Fig 14). Two trials reported benefits in percentage fat or change in percentage fat at 6 and 12 months, which were not statistically significant [66, 94] (Table 11).

**Table 11:** Effect estimates for primary outcomes (physical activity and diet trials).

Clinical Area	Trial	Intervention	Outcome	RR/MD	LCI	UCI
**Diet and physical activity**	Allen 2013 [57]	Smartphone app and intensive counseling versus counseling only	Body weight—change (kg)– 6 months	-2.90	-6.21	0.41
	Allen 2013	Smartphone app and intensive counseling versus counseling only	Body mass index–change (kg/m2)– 6 months	-1.00	-2.10	0.10
	Allen 2013	Smartphone app and intensive counseling versus counseling only	Waist circumference—female (cm)– 6 months	-2.49	-7.21	2.23
	Allen 2013	Smartphone app and intensive counseling versus counseling only	Waist circumference—male (cm)– 6 months	-4.01	-6.06	-1.96
	Carter 2013 [59]	Smartphone app versus website	Body weight (kg)– 6 months	-2.90	-10.98	5.18
	Carter 2013	Smartphone app versus website	Body weight (kg)–change– 6 months	-3.3	-5.4	-1.2
	Carter 2013	Smartphone app versus website	Body mass index (kg/m^2^)– 6 months	-1.90	-4.18	0.38
	Carter 2013	Smartphone app versus website	Body fat (percent)– 6 months	-1.20	-2.96	0.56
	Chow 2015 [82]	SMS versus usual care	LDL cholesterol—6 months	-5	-9	0
	Chow 2015	SMS versus usual care	Sys BP—6 months	-8	-10	-5
	Chow 2015	SMS versus usual care	Dia BP—6 months	-3	-4	-2
	Chow 2015	SMS versus usual care	BMI—6 months	-1.3	-1.6	-0.90
	Chow 2015	SMS versus usual care	Waist circumference cm—6 months	-4.40	-6	-2.8
	Chow 2015	SMS versus usual care	Hip circumference cm—6 months	-4.70	-6.30	-3
	Chow 2015	SMS versus usual care	cholesterol Total—6 months	-9	-15	-4
	Chow 2015	SMS versus usual care	HDL cholesterol—6 months	-0.40	-2	1
	Chow 2015	SMS versus usual care	triglycerides—6 months	-20	-31	-8
	Chow 2015	SMS versus usual care	Heart rate /min– 6 months	-2	-3	-0.40
	Golshahi 2015 [83]	SMS versus pamphlets	SBP (mmHg) change at 8 months	1.07	-0.49	2.63
	Golshahi 2015	SMS versus pamphlets	DBP (mmHg) change at 8 months	0.36	-0.61	1.33
	Haapala 2009 [64]	SMS versus no SMS	Body weight—change (percent)– 12 months	-4.1	-6.73	-1.47
	Haapala 2009	SMS versus no SMS	Body weight (kg)–endpoint– 12 months	-1.90	-7.81	4.01
	Haapala 2009	SMS versus no SMS	Body weight (kg)–change– 12 months	-3.40	-5.72	-1.08
	Haapala 2009	SMS versus no SMS	Waist circumference–end point (cm)– 12 months	−2.00	−6.93	2.93
	Haapala 2009	SMS versus no SMS	Waist circumference–change (cm)– 12 months	-3.9	-6.18	-1.62
	Hebden 2014 [65]	SMS, application, email messages, booklet, dietitian session versus booklet and dietitian session only	body weight (kg)—change at 3 months	-0.30	-1.84	1.24
	Hebden 2014	SMS, application, email messages, booklet, dietitian session versus booklet and dietitian session only	BMI—change at 3 months	-0.11	-0.66	0.43
	Kim 2015 [66]	SMS and educational session versus educational sessions only	weight kg 6 month change	-0.15	-1.37	1.07
	Kim 2015	SMS and educational session versus educational sessions only	weight kg 6 month–end line	1.20	-1.37	3.77
	Kim 2015	SMS and educational session versus educational sessions only	% body fat 6 month change	0.27	-0.76	1.30
	Laing 2014 [67]	app versus encouragement to choose any weight loss activity	Weight—change (kg) - 6 months	-0.30	-1.5	0.95
	Laing 2014	app versus encouragement to choose any weight loss activity	Systolic blood pressure—change (mmHg) - 6 months	-1.7	-7.10	3.8
	Lin 2015 [68]	SMS versus usual care	Weight change (kg) - 6 months	-3.40	-5.40	-1.5
	Lin 2015	SMS versus usual care	Weight end line (kg) - 6 months	-2.9	-10.68	4.88
	Martin 2015b [69]	Smartphone programme incl wireless scale versus health education tips via smartphone	% weight change at 12 weeks	-8.80	-10.19	-7.41
	Martin 2015b	Smartphone programme incl wireless scale versus health education tips via smartphone	mean change in weight at 12 weeks—kg	-7.20	-8.48	-5.92
	Martin 2015b	Smartphone programme incl wireless scale versus health education tips via smartphone	Mean changed in waist circumference at 12 weeks (cm)	-8.60	-11.37	-5.83
	Martin 2015b	Smartphone programme incl wireless scale versus health education tips via smartphone	Change in systolic blood pressure (mm Hg) at 12 weeks	-4.80	-9.72	0.12
	Martin 2015b	Smartphone programme incl wireless scale versus health education tips via smartphone	Change in diastolic blood pressure (mm Hg) at 12 weeks	-4.60	-8.76	-0.44
	Napolitano 2013 [70]	SMS and Facebook versus Facebook only	Body weight–change (kg)– 8 wks	-1.77	-3.44	-0.096
	Patrick 2013 [71]	SMS versus website only	Body mass index (percentile)– 12 months	-0.10	-0.13	-0.07
	Patrick 2013	SMS versus website only	Body mass index (Z-score)– 12 months	0.00	-0.92	0.92
	Patrick 2013	SMS versus website only	Body fat (percent)– 12 months	-0.90	-6.28	4.48
	Ramachandran 2013 [55]	SMS versus standard advice	Body mass index (kg/m2)– 24 months	0.00	-0.92	0.92
	Ramachandran 2013	SMS versus standard advice	Waist circumference—change (cm)– 24 months	0.00	-1.32	1.32
	Ramachandran 2013	SMS versus standard advice	Cumulative incidence of diabetes (no. of participants)– 24 months	0.67	0.49	0.92
	Ramachandran 2013	SMS versus standard advice	Systolic blood pressure (mmHg)– 24 months	0.00	-2.20	2.20
	Ramachandran 2013	SMS versus standard advice	Diastolic blood pressure (mmHg)– 24 months	-0.10	-1.34	1.14
	Ramachandran 2013	SMS versus standard advice	Total cholesterol (mmol/l)– 24 months	0.00	-0.15	0.15
	Ramachandran 2013	SMS versus standard advice	HDL cholesterol (mmol/l)– 24 months	0.10	0.07	0.13
	Ramachandran 2013	SMS versus standard advice	Triglycerides (mmol/l)– 24 months	-0.08	-0.28	0.12
	Shahid 2015 [56]	Voice call delivered feedback versus no feedback	Systolic BP (mmHg)—change at 4 months	-1.66	-1.77	-1.55
	Shahid 2015	Voice call delivered feedback versus no feedback	Diastolic (mmHg)—change at 4 months	-6.02	-6.12	-5.92
	Shahid 2015	Voice call delivered feedback versus no feedback	BMI—change at 4 months	-0.060	-0.077	-0.043
	Shahid 2015	Voice call delivered feedback versus no feedback	HbA1c - change at 4 months	-1.94	-1.95	-1.93
	Shahid 2015	Voice call delivered feedback versus no feedback	LDL—change at 4 months	-13.96	-14.17	-13.75
	Shapiro 2012 [72]	SMS versus E-newsletters	Body weight–change (kg)– 12 months	-0.62	-2.34	1.10
	Steinberg 2013 [93]	SMS versus educational control	Body weight–change (percent)– 6 months	–2.29	–4.66	0.08
	Steinberg 2013	SMS versus educational control	Body weight–change (kg)– 6 months	–2.41	–5.16	0.34
	Steinberg 2013	SMS versus educational control	Body mass index–change (kg/m^2^)– 6 months	–0.89	–1.90	0.12
	Svetkey 2015 [76]	App versus no app	Weight change at 24 months (kg)	0.46	-1.45	2.36
	Svetkey 2015	App versus no app	% weight change at 24 months	0.33	-1.54	2.2
	Turner-McGrievey 2011 [77]	SMS and podcast versus SMS only	Body weight–change (percent)	0.00	-2.15	2.15
	Varnfield 2014 [80]	Mixed mobile technology versus usual care	Systolic BP—change at 6 wks	2.53	-3.63	8.68
	Varnfield 2014	Mixed mobile technology versus usual care	Diastolic BP—change at 6 wks	4.19	0.44	7.93
	Varnfield 2014	Mixed mobile technology versus usual care	Heart rate (resting BPM)—change at 6 wks	1.4	-2.23	5.03
	Varnfield 2014	Mixed mobile technology versus usual care	weight (kg)—change at 6 wks	0.96	-0.28	2.2
	Varnfield 2014	Mixed mobile technology versus usual care	Waist circumference (cm)—change at 6 wks	-0.18	-1.85	1.48
	Varnfield 2014	Mixed mobile technology versus usual care	total cholesterol (mmol/L)—change at 6 wks	-0.26	-0.65	0.13
	Varnfield 2014	Mixed mobile technology versus usual care	LDL (mmol/L)—change at 6 wks	-0.11	-0.42	0.2
	Varnfield 2014	Mixed mobile technology versus usual care	HDL (mmol/L)—change at 6 wks	-0.06	-0.18	0.060
	Varnfield 2014	Mixed mobile technology versus usual care	Triglycerides (mmol/L)—change at 6 wks	-0.26	-0.51	-0.01
	Varnfield 2014	Mixed mobile technology versus usual care	HbA1C (mmol/L)—change at 6 wks	-0.31	-1.13	0.52
	Wong 2013 [81]	SMS versus usual care	Body mass index (kg/m2)– 24 months	-1.07	-2.29	0.15
	Wong 2013	SMS versus usual care	Body weight (kg)– 24 months	-3.44	-7.53	0.65
	Wong 2013	SMS versus usual care	Waist circumference (cm)– 24 months	-2.38	-5.47	0.71
	Wong 2013	SMS versus usual care	Cumulative incidence of diabetes (no. of participants)– 24 months	0.62	0.24	1.61
	Wong 2013	SMS versus usual care	Diastolic blood pressure (mmHg)– 24 months	-1.89	-6.43	2.65
	Wong 2013	SMS versus usual care	Systolic blood pressure (mmHg)– 24 months	1.22	-5.54	7.98
	Wong 2013	SMS versus usual care	Total cholesterol (mmol/l)– 24 months	-0.14	-0.48	0.20
	Wong 2013	SMS versus usual care	HDL cholesterol (mmol/l)– 24 months	0.01	-0.09	0.11
	Wong 2013	SMS versus usual care	LDL cholesterol (mmol/l)– 24 months	-0.16	-0.46	0.14
	Wong 2013	SMS versus usual care	Triglycerides (mmol/l)– 24 months	-0.28	-0.90	0.34
	Wong 2013	SMS versus usual care	Fasting plasma glucose levels (mmol/l)– 24 months	-0.05	-0.28	0.18
	Wong 2013	SMS versus usual care	2 hour postprandial glucose levels (mmol/l)– 24 months	-0.52	-1.55	0.51

**Fig 15:**
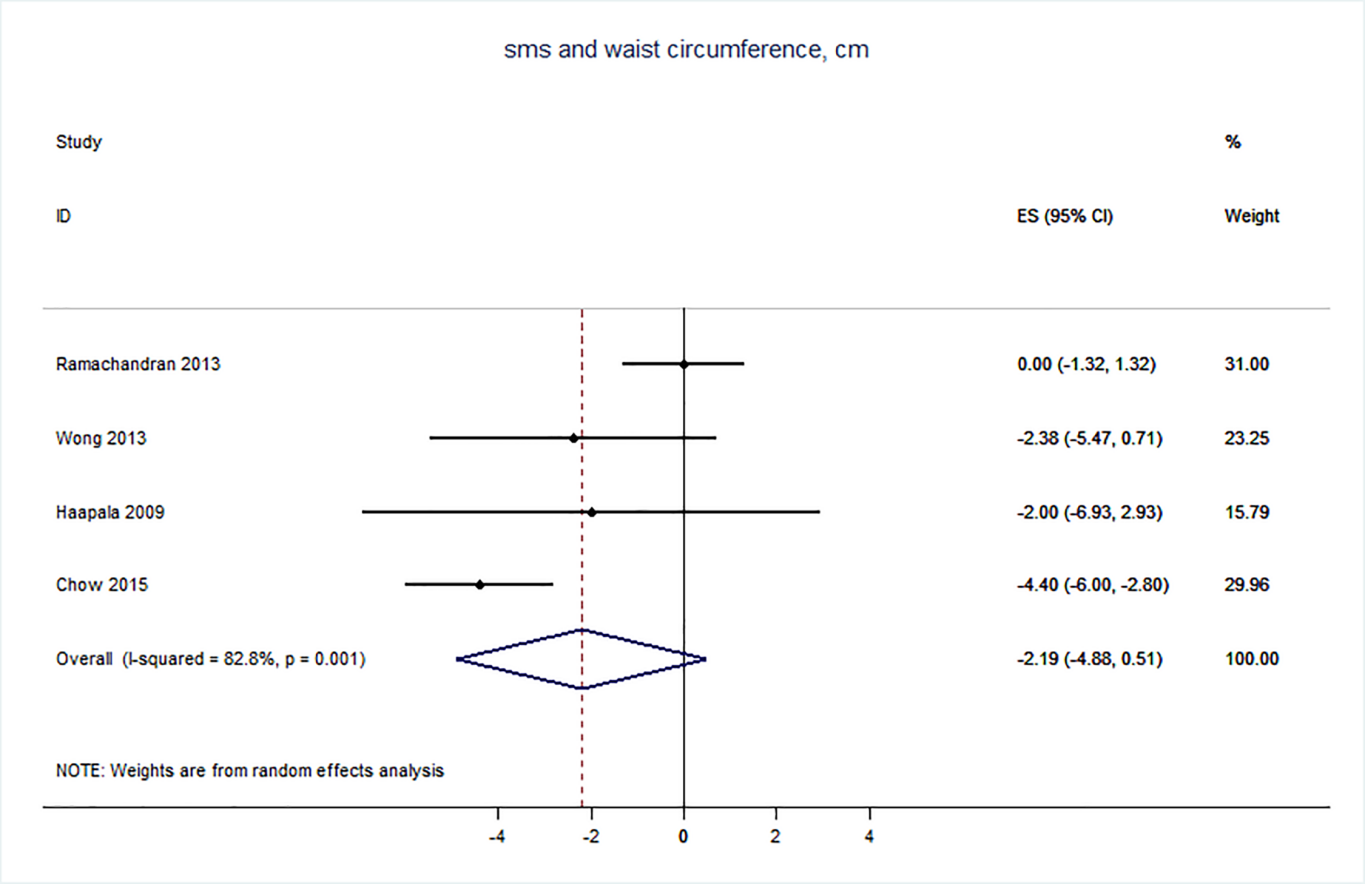
Physical activity and diet interventions using SMS function–waist circumference endpoint (cm).

Pooled analysis of four trials examining the effect of SMS-based interventions showed no statistically significant effect on waist circumference measured between 6 and 24 months [55, 64, 81, 82] (pooled MD -2.19 [95% CI -4.88, 0.51] p = 0.112, I^2^ = 82.8%) (Fig 15). However, one of these trials demonstrated a significant effect when the outcome was change in waist circumference at 12 months [64]. One trial reported a statistically significant reduction in hip circumference at 6 months [82]

**Fig 16:**
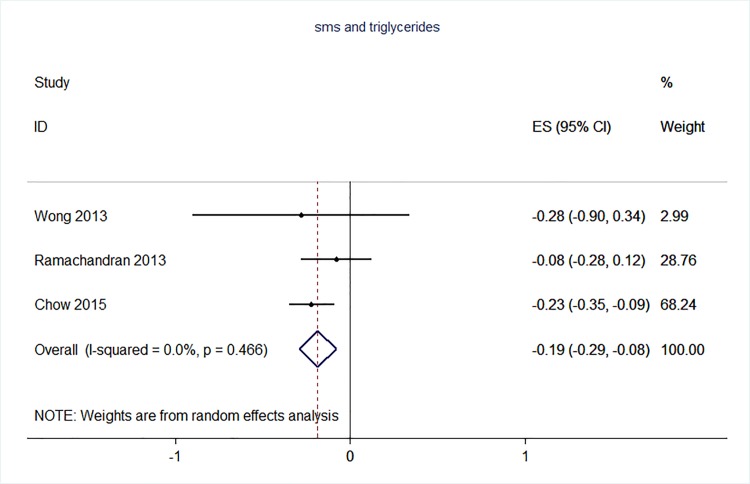
Physical activity and diet interventions using SMS function–triglycerides (mmol/L).

**Fig 17:**
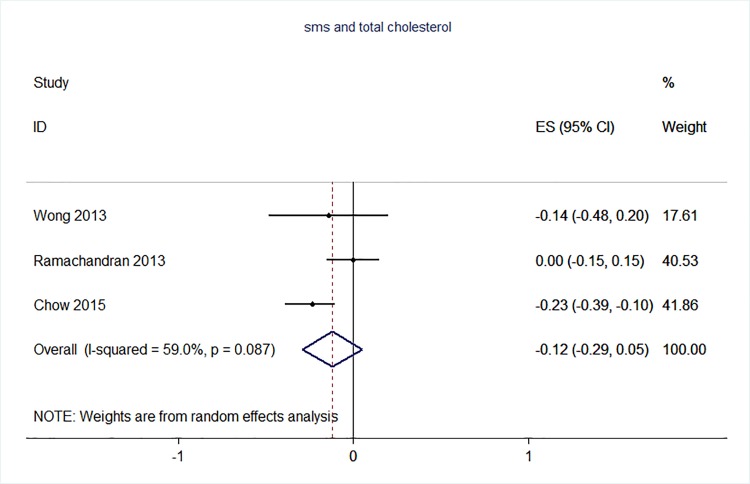
Physical activity and diet interventions using SMS function–total cholesterol (mmol/L).

**Fig 18:**
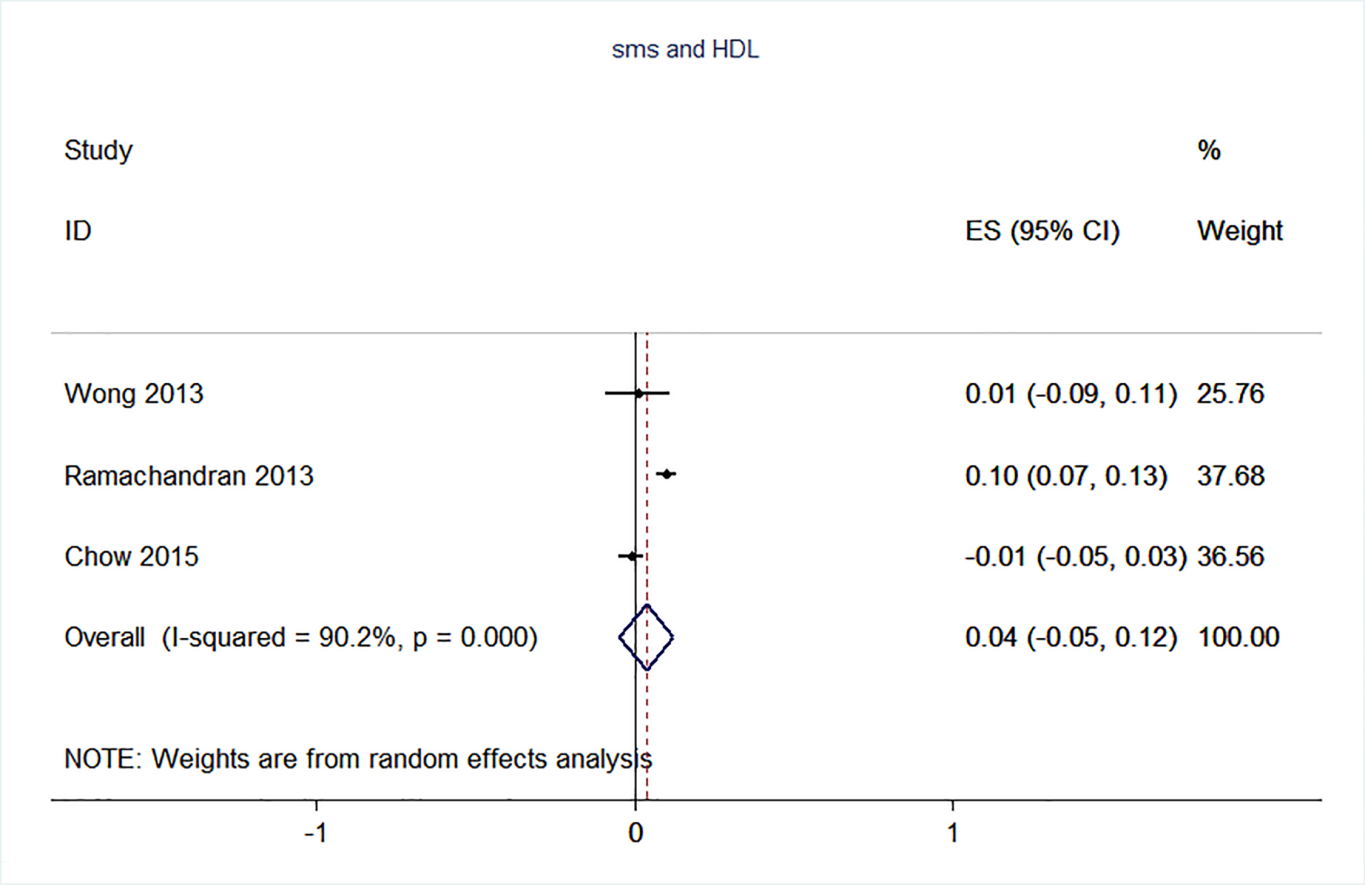
Physical activity and diet interventions using SMS function–high density lipoprotein cholesterol (mmol/L).

**Fig 19:**
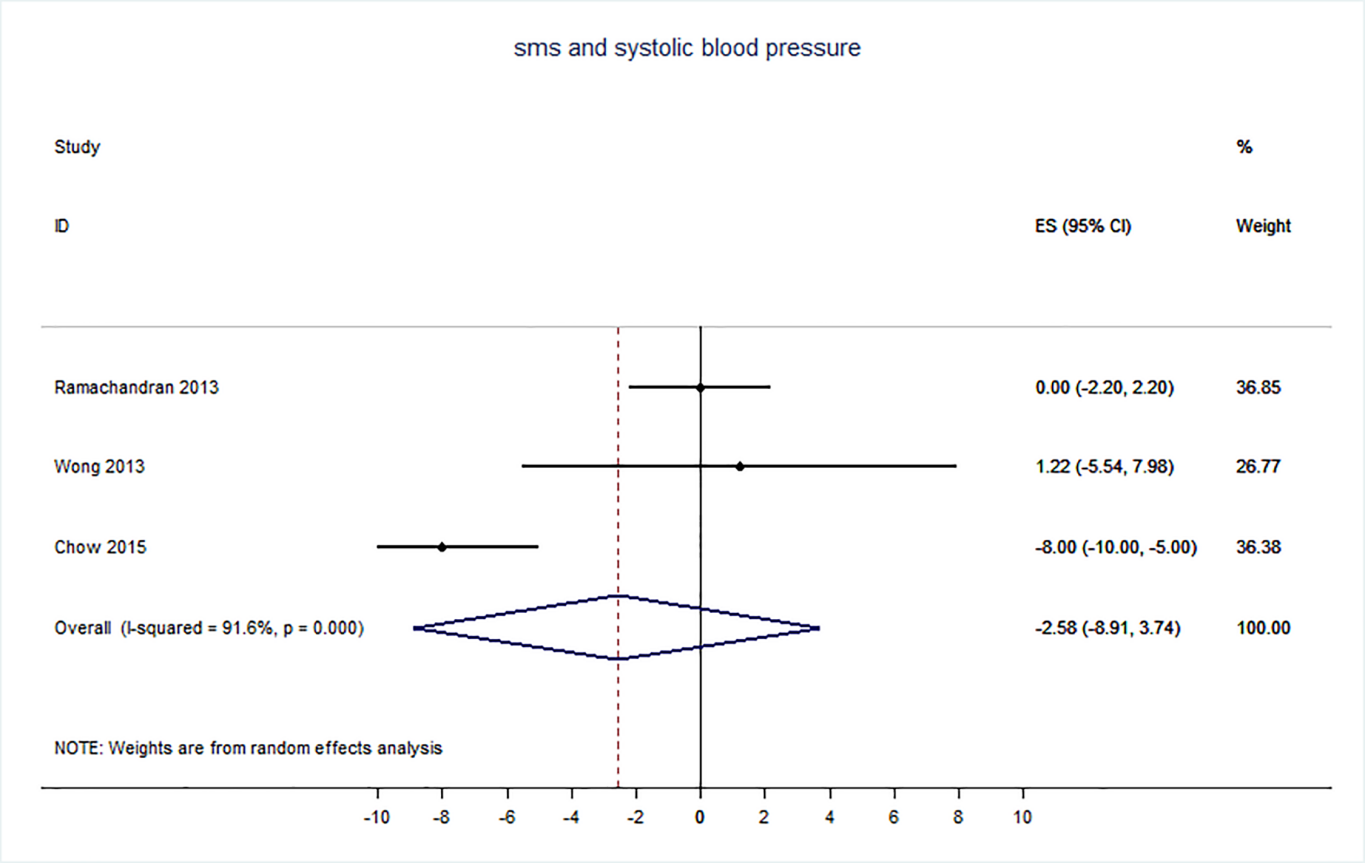
Physical activity and diet interventions using SMS function–systolic blood pressure (mmHg).

**Fig 20:**
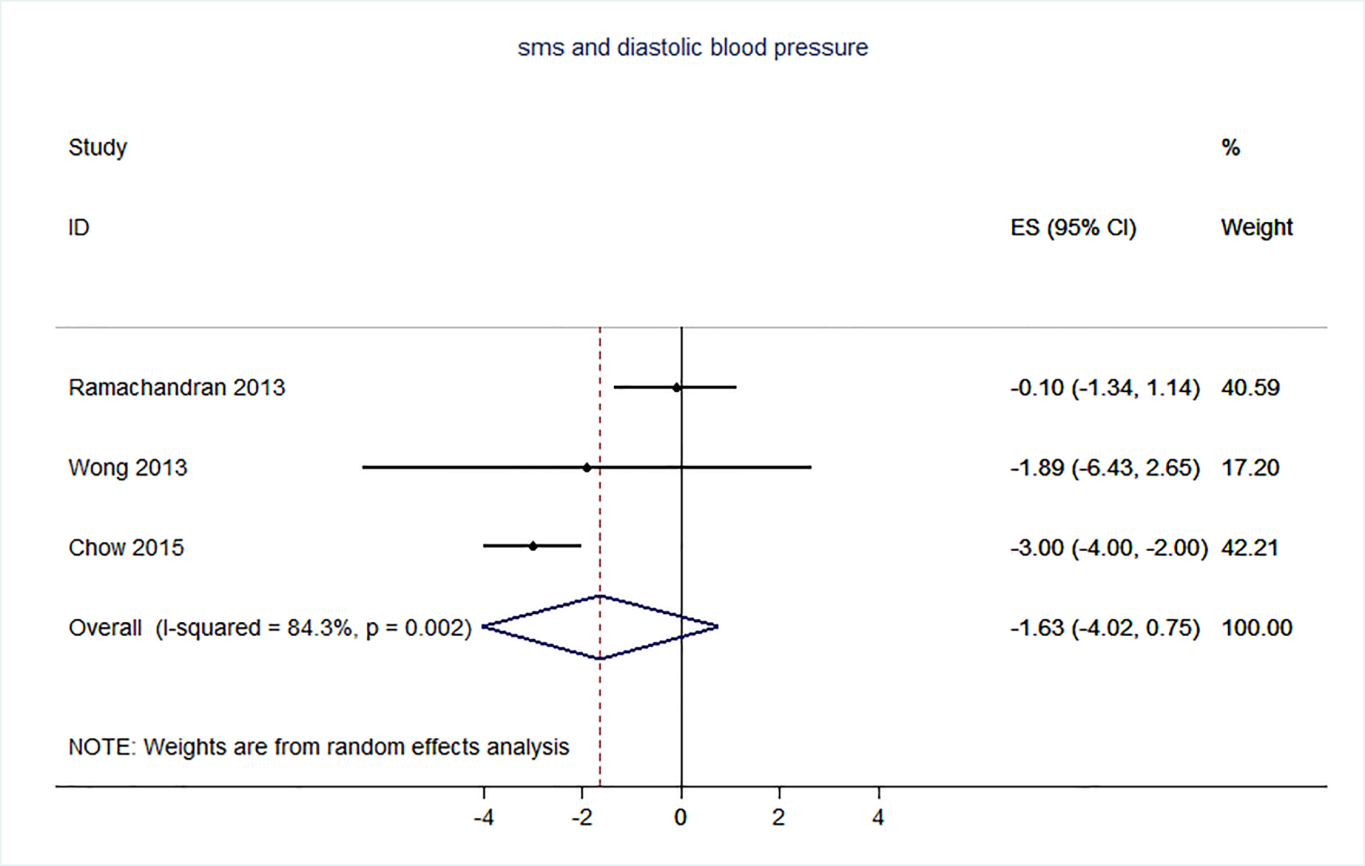
Physical activity and diet interventions using SMS function–diastolic blood pressure (mmHg).

**Fig 21:**
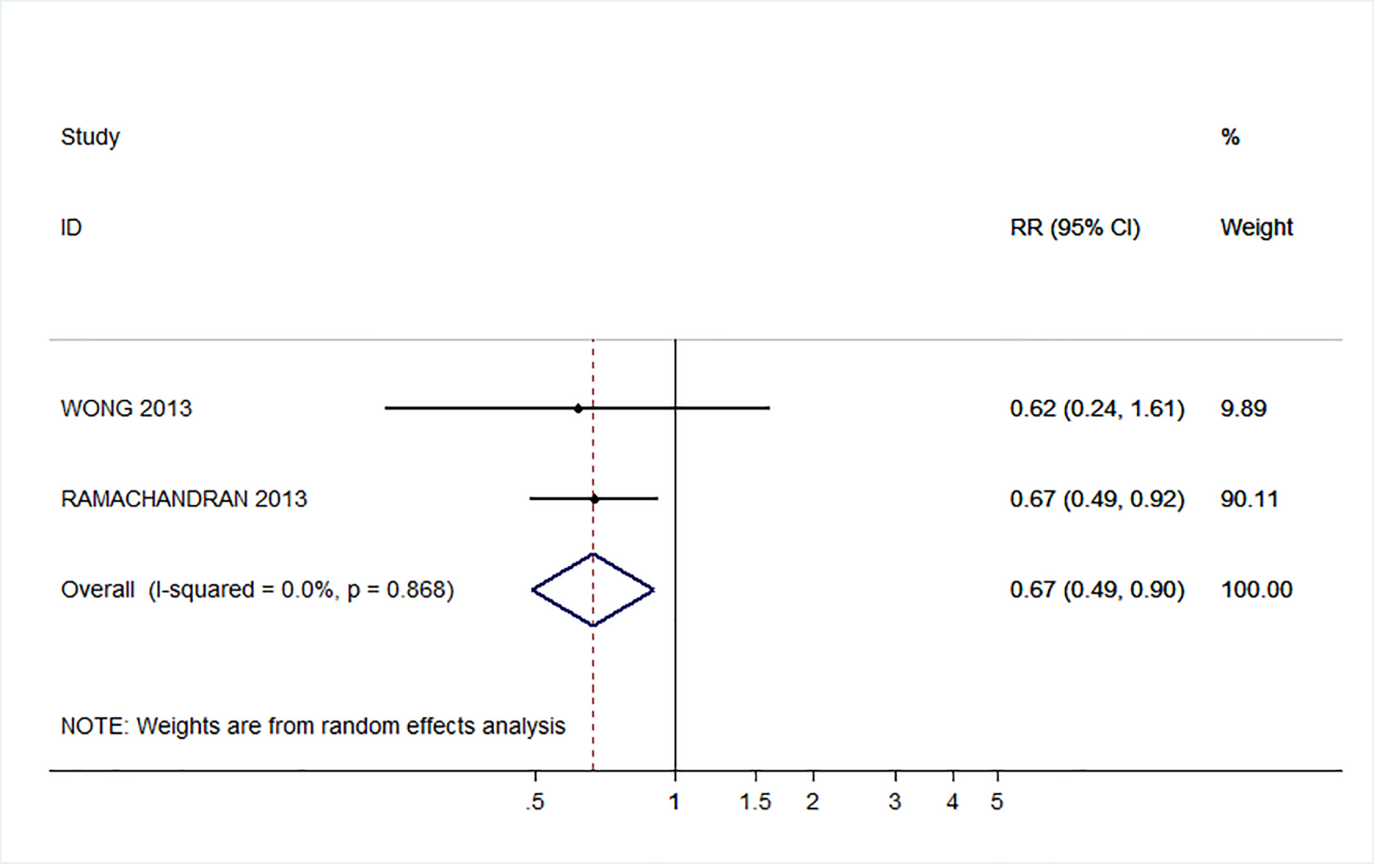
Physical activity and diet interventions using SMS function–cumulative incidence of diabetes.

Interventions delivered by SMS alone: bio-medical outcomes. Pooled analyses of three trials [55, 81, 82] of SMS-based interventions aiming to increase physical activity and improve diet found evidence of a statistically significant reduction in triglyceride levels (pooled MD -0.19 mmol/L [95% CI -0.29, -0.08, p-value = 0.001], I^2^ = 0.0%) (Fig 16). Pooled effects were heterogenous (I squared 59–90%), not statistically significant but in the direction of benefit for total cholesterol, high density lipoprotein cholesterol, systolic blood pressure and diastolic blood pressure, with one trial reporting statistically significant improvements (Figs 17, 18, 19 and 20). One trial also found a borderline significant effect in reducing LDL cholesterol and a significant reduction in heart rate [82]. Another of these trials showed no effect on glucose levels [81]. A trial of an SMS-based intervention vs information in a pamphlet also showed no effect on systolic or diastolic blood pressure [83]. Pooled analysis of two studies examining the effect of SMS based interventions for weight management showed a statistically significant reduction in the cumulative incidence of diabetes [55, 81] (pooled RR 0.67 [95% CI 0.49, 0.90], I^2^ = 0.0%) (Fig 21).

**Fig 22:**
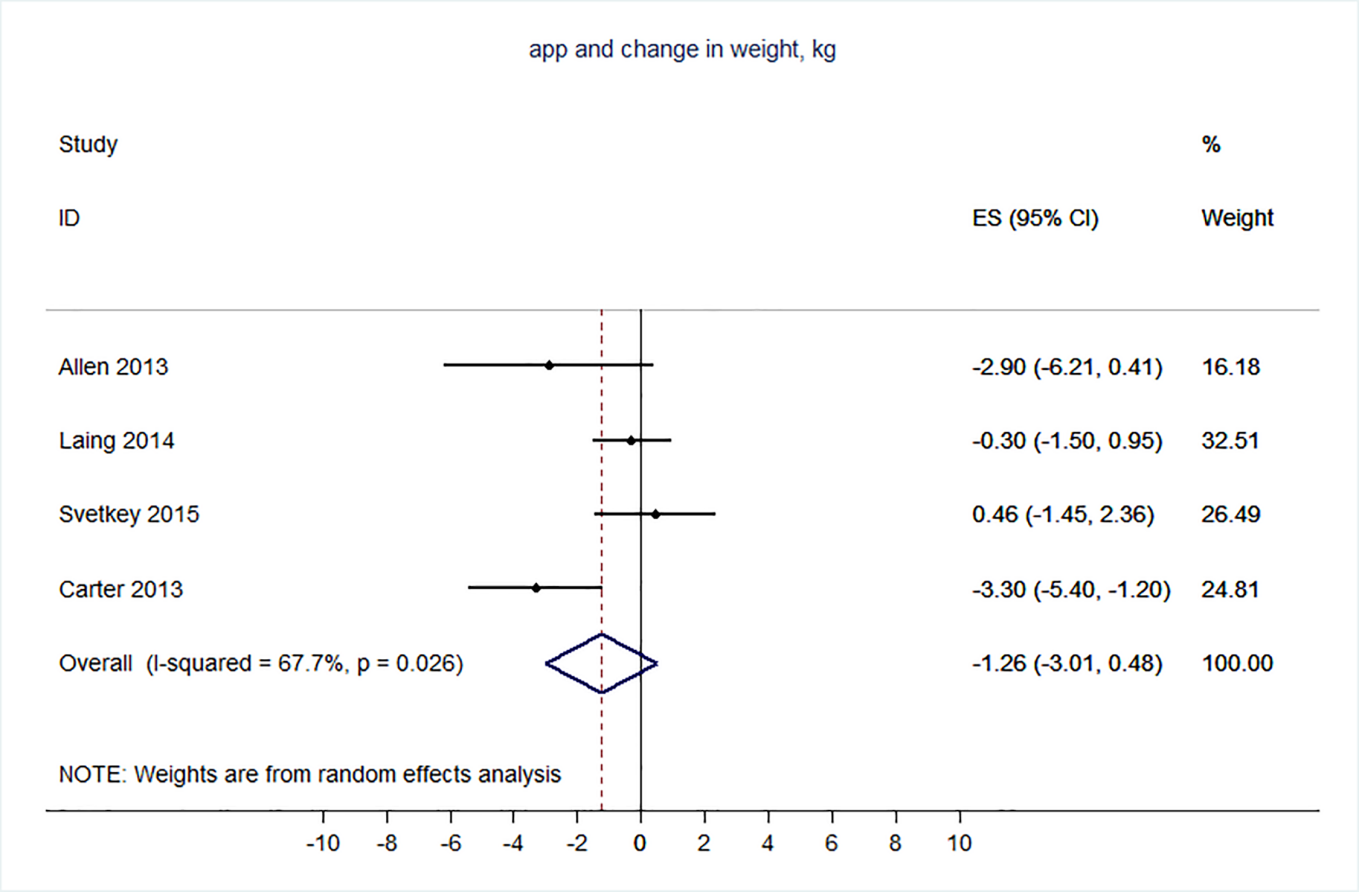
Physical activity and diet interventions using application function–change in weight (kg).

Interventions delivered by other or mixed mobile technology media: anthropometric outcomes. Pooled analysis of four studies [57, 59, 67, 76] found no evidence that interventions delivered through mobile apps resulted in greater change in weight when measured between 6 and 24 months (pooled MD -1.26 [95% CI -3.01, 0.48] p-value = 0.156, I^2^ = 67.7%) (Fig 22). A trial of a mobile phone activity monitor intervention reported no statistically significant changes in weight or waist circumference at 9 months [78]. A trial assessing an intervention delivered by SMS and app showed no significant effect on weight loss at 3 months (Hebden et al., 2014).

There was no statistically significant change in BMI for two trials that used smartphone weight loss applications at 6 months and 24 months [57, 59], or at 3 months in a trial of an intervention delivered by SMS and app [65]. A trial examining the effect of a podcast plus SMS intervention versus podcast-only found no effect on percentage change in body weight [77]. A trial of a multi-component smartphone-based intervention involving text messages, audio and video files, pre-installed health diary and activity monitoring, a blood pressure monitor and weight scale reported no statistically significant change in weight or waist circumference [80]. In Martin and colleagues’ [69] study involving overweight and obese adults, intervention participants were instructed to weigh themselves daily on a bathroom scale and to wear an activity monitor provided to them. This wirelessly transmitted data to a website accessible to a counsellor and feedback was delivered through a loaned smartphone via text, phone calls, email. This intervention had a significant effect on weight loss (MD -7.20 kg [95% CI -8.48, -5.92]), in addition to reductions in waist circumference. A trial of a mobile-app had no effect on waist circumference among women, but there was evidence of a statistically significant reduction among men [57] (Table 11).

Interventions delivered by other or mixed mobile technology media: bio-medical outcomes. A trial of a mobile-app intervention showed no effect on blood pressure [67], while a study of a voice-call delivered intervention demonstrated an effect on lowering blood pressure, HbA1c, and LDL cholesterol [56]. An intervention delivered by mixed mobile phone media showed statistically significant reductions in diastolic blood pressure and a reduction which was not statistically significant in systolic blood pressure [69]. A trial of a multi-component smartphone-based intervention involving text messages, audio and video files, pre-installed health diary and activity monitoring, a blood pressure monitor and weight scale, showed no statistically significant effect on cholesterol, HbA1c, heart rate, or systolic blood pressure, but had a statistically significant effect in reducing triglycerides, and increasing diastolic blood pressure [80].

#### Physical activity, diet and physical activity, diet–secondary outcomes

Of 128 self-reported outcomes 18 demonstrated statistically significant benefits and none showed statistically significant harms (S2 Table).

#### Alcohol–primary outcomes

There were no objectively measured outcomes reported in trials of alcohol reduction interventions.

#### Alcohol–secondary outcomes

For trials targeting alcohol consumption, one trial delivering supportive SMS observed a statistically significant increase in the number of days to first drink after inpatient discharge but no statistically significant effect on drinking frequency or cognitive outcomes [84]. A trial of an SMS-based drinking assessment intervention found a statistically significant reduction in the number of binge drinking days and number of drinks per drinking day among the intervention group receiving real time feedback, but no such effect in the intervention group who did not receive feedback [86]. A second trial delivering a smartphone intervention observed a small but statistically significant effect on the number of risky drinking days but no effect on continuous abstinence [87]. Another trial observed no effect of SMS-based drinking assessments and brief interventions on drinking frequency in young adults discharged from the Emergency Department [90]. One trial found a small but statistically significant beneficial effect of an intervention delivered by interactive voice response on a multi-item scale measuring alcohol consumption, alcohol dependence and alcohol-related harm [85]. A study among Swedish students assessing the effect of an application-based intervention found that those in the intervention group had slightly increased alcohol consumption [86] (S2 Table).

### Quality of evidence assessment

The assessment of quality of evidence for pooled outcomes is reported in Table 12.

**Table 12:** Quality assessment for pooled outcomes.

Quality assessment for objective outcomes in pooled analyses							Summary of findings			Importance
Number of studies	Study design	Risk of Bias	Inconsistency	Indirectness	Imprecision	Other	Number of participants	Effects	quality	
								**Relative or absolute**		
**Biochemically verified smoking cessation: SMS support in smokers willing to make a quit attempt**										
5	RCT	1 trial with 151 participants high risk bias	none	none	none	none	8359	Smoking cessation: Biochemically verified continuous abstinence: RR 2.19 (95% CI 1.80, 2.67) (2 trials) Smoking cessation: Biochemically verified Point prevalence: RR 1.51 (95% CI 1.06, 2.15) (4 trials)	++++ high	critical
**Smoking cessation SMS support during a quit attempt: Adverse events**										
3	RCT	Low risk of bias	none	none	none	none	7705	Self-reported car accidents: RR 1.01 (95% CI 0.71, 1.42) (3 trials) Self-reported thumb strain: RR 1.02 (95% CI 0.83, 1.25) (3 trials) No other adverse event reported in open feedback	++++ high	critical
**Diet and Physical activity interventions delivered by SMS**										
3	RCT	High risk bias	serious	none	none		1351	BMI md -0.84 (95% CI -1.69–0.01)	++ low	critical
**6**	RCT	High risk of bias	serious	none	none		698	Weight loss (kg) md -1.77 (95% CI-2.95, -0.58)	++ low	critical
**2**	RCT	High risk of bias	low	none	none		175	Weight loss (%) md -3.10 (95% CI-4.86, -1.34)	+ low	critical
**4**	RCT	High risk of bias	serious	none	serious		558	Weight endpoint (kg) md 0.99 (95% CI -3.63, 1.64)	+ very low	critical
4	RCT	High risk of bias	serious	none	none		1476	Waist circumference (cm) md -2.19 (95% CI -4.88, 0.51)	+ very low	critical
3	RCT	High risk of bias	low	none	none		1351	Triglycerides (nmol/L) md -0.19 (95% CI -0.29, -0.08)	+ very low	critical
3	RCT	High risk of bias	serious	none	none		1351	Total cholesterol (nmol/L) md -0.12 (95% CI -0.29, 0.05)	+ very low	critical
3	RCT	High risk of bias	serious	none	serious		1351	High density lipoprotein (nmol/L) md 0.04 (95% CI -0.05, 0.12)	+ very low	critical
3	RCT	High risk of bias	serious	none	serious		1351	Systolic blood pressure (mmHg) md -2.58 (95% CI -8.91, 3.74)	+ very low	critical
3	RCT	High risk of bias	serious	none	None		1351	Diastolic blood pressure (mmHg) md -1.63 (95% CI -4.02, 0.75)	+ very low	critical
2	RCT	High risk of bias	low	none	None		641	Cumulative incidence of diabetes RR 0.67 (95% CI 0.49, 0.90)	++ low	critical
**Physical activity and diet interventions delivered by app**										
4	RCT	High risk of bias	serious	None	None		773	Weight loss (kg) MD -1.26 (95% CI -3.01, 0.48)	++ low	critical
**Physical activity interventions delivered by SMS**										
3	RCT	Low/unclear risk of bias	Serious	None	None		193	Change in daily step count MD 1256.9 (95% CI -159.7, 2673.6)	++ low	important

According to the GRADE [19] criteria (Table 12), there was high quality evidence of benefit for smoking cessation support delivered by text message and no evidence of harms. For SMS based physical activity interventions, there was low quality evidence of changes in physical activity which was not statistically significant. For SMS based diet and physical activity interventions there was low quality evidence suggesting benefit in reducing the incidence of diabetes in those with pre diabetes and modest or small benefits in change in weight (KG or %), BMI and triglycerides. The evidence of benefit for end point weight, waist circumference, total cholesterol, and blood pressure was very low, with one trial at low risk of bias conducted in those with coronary heart disease reporting statistically significant improvements. The evidence of benefit on HDL cholesterol was very low with one trial reporting statistically significant improvements. The effect of diet and physical activity interventions delivered by app was in the direction of a small benefit, but not statistically significant.

## Discussion

### Key findings

We identified 71 trials of interventions delivered by mobile phone targeting prevention of NCD focussed on smoking cessation, physical activity, diet, and alcohol reduction. No trials reported effects on morbidity or mortality.

There is high quality evidence that smoking cessation support delivered by text message for smokers making a quit attempt increases smoking cessation in trials conducted in high income countries and no evidence for adverse effects of these interventions. In single trials there was no suggestion that text messages to prompt a quit attempt and link people with existing smoking cessation services are more effective than a leaflet with the same content [24]. There was low quality evidence that phone counseling by mobile phone increased smoking cessation at 3 months [35].

For physical activity interventions delivered by SMS, App or fit bit, trials reporting outcomes at 3 months or longer showed no evidence of benefit. The effects of multiple monitoring devices was mixed with statistically significant benefits in only one of 12 biomedical outcomes at 12 weeks.

For SMS based diet and physical activity interventions there was low quality evidence suggesting benefit in reducing the incidence of diabetes in those with pre diabetes and for modest or small benefits in change in weight (KG or %), BMI and triglycerides. The evidence of effects on end point weight, waist circumference, total cholesterol, and blood pressure were in the direction of benefits, but not statistically significant and highly heterogeneous in pooled analyses, with one trial at low risk of bias conducted in those with coronary heart disease reporting statistically significant improvements. The evidence of benefit on HDL cholesterol was heterogeneous and very low quality, with one trial reporting statistically significant improvements. The effect of diet and physical activity interventions delivered by app on change in weight at 6 months or longer was in the direction of a small benefit, but not statistically significant. There was mixed evidence regarding benefits of interventions delivered by multiple smartphone media.

There were some promising but inconclusive self-reported effects from alcohol reduction trials.

### Strengths and weaknesses of the review

This is a comprehensive review of all randomised controlled trials of interventions delivered by mobile phone designed to prevent non-communicable diseases. Our review has several strengths. The methods are reproducible, screening for trials and data extraction was conducted by two researchers, we used standard Cochrane tools for assessing risk of bias and used the GRADE criteria for assessing the overall quality of evidence. We only included randomised controlled trials, which are less prone to bias than other types of controlled study. We did not pool the results of interventions delivered by mobile phone with those delivered by mobile phone in conjunction with non mobile phone based components, as has been done in some previous reviews [10–12]. Only six trials did not provide sufficient data to calculate effect estimates.

There are also a number of limitations to our review. It was not possible to contact authors for data in this review, due to time and funding constraints. It was beyond the scope of our review to include interventions delivered by PDA or hand held computer or to review all internet or video based interventions, which in principal can be viewed on many modern mobile phones. Our review aimed to examine the effects of interventions delivered by mobile technologies alone. We excluded interventions combining mobile technologies with additional interventions such as face-to-face counselling, which could be subject to a separate systematic review. We only pooled the results of trial where the trial aim, outcomes and mobile phone media used were the same (e.g. SMS, application software). Nonetheless some of the results of pooled analyses were heterogeneous. This is likely to be due to the wide range of factors which could influence the effectiveness of particular mobile technology interventions including: trial quality [95], participant factors, the setting (low/middle or high income country), intervention design, intervention components (e.g. the behaviour change techniques employed), intensity or intervention duration. We only pooled objectively measured outcomes in meta-analyses due to prior evidence that self-reported outcomes in behaviour change trials can be prone to overstated benefits. Some evidence in our review supports this, for example, a smoking cessation trial showed a null result for a biochemically-confirmed measure, but a benefit in the equivalent self-report outcome [21]. Our review provides no insight into the mechanism of action of interventions. The examination of funnel plots in exploring publication bias was limited as few trials contributed to some pooled analyses.

The scope of the review encompasses trials of novel non-communicable disease prevention interventions delivered by mobile phone. It is plausible that in lower and middle income countries the main benefits of mobile phones would not be evaluated by RCT for example simply owning a phone might afford the potential for the first time for people to gain information about and access to existing health promotion interventions and services.

### Discussion in relation to existing literature

We provide an updated systematic review of evidence regarding the effects of interventions for smoking cessation, physical activity, diet and alcohol. Based on short term outcomes, self reported outcomes and non randomised study designs previous reviews have concluded that there are benefits of interventions targeting physical activity and /or diet. Our review demonstrated there is no reliable evidence of benefit for physical activity interventions delivered by SMS, app or fit bit at 3 months or longer. Whilst some diet and physical activity interventions have reported benefits, the overall quality of evidence for even modest benefits is low or very low. Our results are similar to the findings for existing systematic reviews of alcohol reduction interventions and show some promising findings but overall very low quality evidence for their benefits [96]. In contrast to the existing Cochrane review of smoking cessation interventions delivered by mobile phone we did not pool objective outcomes with self reported outcomes and we examined the effects of different mobile phone media (SMS, app, video, interactive voice and phone counselling) separately. The rational for this was that there are important differences between different mobile phone media. For example; SMS messages are sent directly to peoples phones, flash up on screens, and can be stored to be re-read at convenient times, whilst apps are dependent on motivated participants going to the app to view content (unless apps also use instant messages), voice messages are often only accessible when sent and cannot always be reviewed. This decision appears to be supported by the results of the review. Our resulting pooled analyses of the effects of SMS based smoking cessation support during a quit attempt demonstrated clear benefits without heterogeneity [13], but there was no suggestion of benefit for smoking cessation interventions delivered by video or interactive voice recording [13, 29].

In comparison to the findings of our 2013 review [15], this review confirms that SMS based smoking cessation support increases quitting in those willing to make a quit attempt, with a larger number of trials now included in the meta- analysis. Since our last review trials have been published showing that interventions designed to prompt a quit attempt have not shown clear benefits. This review includes recently published evidence of benefits in diet and physical activity interventions in preventing the onset of diabetes, in those with pre-diabetes. There is still no clear evidence of benefit of physical activity, diet and physical activity or dietary interventions for other populations at 3 months or longer. There are a larger number of alcohol reduction trials included in this review (8 compared to 1), but whilst the effects of some alcohol reduction interventions look promising the results are still inconclusive.

### Meaning of the study, implications for clinicians or policy makers

Continuous abstinence is considered the gold standard for smoking cessation trials. Estimates of effect using biochemically verified point prevalence for smoking cessation support delivered by SMS were lower than continuous abstinence effect estimates. This would be expected as point prevalence estimates are diluted by quit attempts occurring randomly throughout the follow up period in both intervention and control groups but unrelated to the intervention. The interventions for smoking cessation included in our pooled analyses contained at least 8 behaviour change techniques used in effective face to face smoking cessation support but adapted for delivery by text message. SMS support for smoking cessation is highly cost-effective [97] and has been implemented in New Zealand, UK, USA and India. In England between 60,000 and 90,000 people have registered for smoking cessation support delivered by text message each year since 2012. An evaluation of implementation showed that the 4 week quit rates achieved were similar or higher than in the UK trial [98]. An evaluation of implementation of an interventions delivered by SMS in India is ongoing. Health services should consider implementing smoking cessation support delivered by SMS with similar content to interventions found to be effective in trials.

Whilst some trials report benefits, there is currently insufficient evidence of beneficial effects on long term objective outcomes to warrant implementation of interventions for physical activity or diet and physical activity. The statistically significant effects of diet and physical activity interventions on change in weight, illustrates the greater power of these outcomes compared to absolute weight outcomes, which did not achieve statistical significance. The effects of behavioural support for weight loss delivered by text message is broadly consistent with the modest benefits achieved by behavioural support for weight loss in general [99, 100].

Few trials target smoking diet and physical activity, but there is no evidence from existing trials to suggest that targeting multiple behaviours reduces the intervention effects for individual behaviours. Most interventions were delivered by text message. There was no good evidence of benefit of app based interventions for smoking cessation, physical activity, diet or alcohol reduction.

### Unanswered questions and future research

Further evaluations of the impact and costs of smoking cessation interventions delivered by mobile phone in low and middle income settings, are needed.

There is a considerable body of existing research regarding effective interventions for behaviour change and much of this literature suggests multifaceted interventions are required [101–105]. Interventions should be developed using established methods including: needs assessment, reviewing the evidence regarding factors influencing the target outcome, drawing on behaviour change theory and evidence based behavioural change techniques and adapting content based on user views regarding the acceptability, comprehensibility and relevance of intervention content [106–108]. However, the impact of even the most well developed interventions delivered by mobile phone for preventative behaviours such as diet and physical activity, which are strongly influenced by environmental factors, are likely to be modest. Nonetheless, intervention delivery costs are low, so adequately powered high quality trials of optimised interventions targeting diet and physical activity are required to reliably establish their effects, especially in high risk groups such as for diabetes prevention in those with pre-diabetes. The effects of interventions targeting diet, physical activity and smoking cessation on morbidity and mortality in populations with existing coronary heart disease should be established.

Further research is required to evaluate the mechanism of action of interventions. Understanding the mechanism of action of interventions could inform the content of future interventions. A range of questions regarding the effects of mobile technologies remain open to question including whether some intervention functions are more effective delivery tools than others (SMS, video, oral communication, application software), which behaviour change techniques are effective when modified for delivery by mobile phone and whether the effectiveness of interventions is influenced by setting or participant demographics.

Whilst some trials have been conducted in low and middle income countries, the majority of the research has been conducted in high-income countries. In view of the high coverage of mobile technologies in these settings, trials of interventions in low and middle-income countries are required.

## Conclusion

In high income settings, SMS based smoking cessation support during a quit has been shown to result in increases in smoking cessation. Effective interventions included 8 or more behaviour change techniques. The effects, costs and impact of interventions in low income countries should be established. There is only weak evidence regarding the benefits of diet physical activity and alcohol reduction interventions delivered by mobile phone. Large scale high quality trials of the effects of optimised interventions on morbidity and mortality, especially in high risk groups, are warranted.

## Supporting information

S1 Text Medline search strategy. (DOCX)

S2 Text Review protocol. (DOCX)

S3 Text Prisma checklist. (DOC)

S1 Table Risk of bias assessments for individual trials. (DOCX)

S2 Table Effect estimates for secondary outcomes. (DOCX)
