# Supplementary material for: The effectiveness of smoking cessation, physical activity/diet and alcohol reduction interventions delivered by mobile phones for the prevention of non-communicable diseases: A systematic review of randomised controlled trials
Source: PLoS One. 2018 Jan 5;13(1):e0189801. doi: 10.1371/journal.pone.0189801 (PMC5755775; doi:10.1371/journal.pone.0189801)
Supplement: S1 Text — (DOCX) [file pone.0189801.s001.docx]

MEDLINE search strategy

| **#** | **Searches** | **Results** |
| --- | --- | --- |
| 1 | computers, handheld/ | 2341 |
| 2 | (computer$ adj2 handheld).ab,ti. | 392 |
| 3 | (computer$ adj2 hand-held).ab,ti. | 246 |
| 4 | (computer$ adj2 palm$).ab,ti. | 155 |
| 5 | (computer$ adj2 pocket).ab,ti. | 102 |
| 6 | (computer$ adj2 mobile).ab,ti. | 151 |
| 7 | (Pocket-PC$ or PocketPC$).ab,ti. | 50 |
| 8 | (Pocket adj computer$).ab,ti. | 87 |
| 9 | (Palm adj3 PDA$).ab,ti. | 15 |
| 10 | (PDA$ adj3 computer$).ab,ti. | 52 |
| 11 | (Personal adj digital adj assistant$).ab,ti. | 835 |
| 12 | (PDA$ adj3 phone$).ab,ti. | 42 |
| 13 | (PDA$ adj3 telephone$).ab,ti. | 3 |
| 14 | (Tablet adj computer$).ab,ti. | 124 |
| 15 | (Tablet adj PC$).ab,ti. | 105 |
| 16 | (Palm-Pilot$ or Palmpilot$).ab,ti. | 46 |
| 17 | (Palm adj (Pre or Treo or Centro)).ab,ti. | 3 |
| 18 | (smartbook$ or smart-book$).ab,ti. | 1 |
| 19 | ((ultra-mobile or ultramobile) and (PC$ or personal computer$)).ab,ti. | 10 |
| 20 | ((ultra-portable or ultraportable) and (PC$ or personal computer$)).ab,ti. | 1 |
| 21 | (enterprise adj digitial adj assistant$).ab,ti. | 0 |
| 22 | (EDA$ adj3 computer$).ab,ti. | 3 |
| 23 | cellular phone/ | 4540 |
| 24 | (cellular adj2 phone$).ab,ti. | 546 |
| 25 | (mobile adj2 phone$).ab,ti. | 2630 |
| 26 | (mobile adj2 telephone$).ab,ti. | 346 |
| 27 | (cellular adj2 telephone$).ab,ti. | 333 |
| 28 | (cell adj2 phone$).ab,ti. | 1087 |
| 29 | (cell adj2 telephone$).ab,ti. | 16 |
| 30 | (smartphone$ or smart-phone$).ab,ti. | 805 |
| 31 | ((blackberr$ or black-berr$) adj3 (mobile$ or phone$ or computer$)).ab,ti. | 6 |
| 32 | (google adj3 phone$).ab,ti. | 2 |
| 33 | (nexus adj one adj3 phone$).ab,ti. | 0 |
| 34 | (application adj software).ab,ti. | 149 |
| 35 | (MMS or multimedia messag$ service$).ab,ti. | 3201 |
| 36 | (SMS or short messag$ service$).ab,ti. | 2883 |
| 37 | (text$ adj messag$).ab,ti. | 856 |
| 38 | MP3 player/ | 128 |
| 39 | MP3 player$.ab,ti. | 71 |
| 40 | MP4 player$.ab,ti. | 1 |
| 41 | (MP3 or MP4).ab,ti. | 391 |
| 42 | (portable adj2 media adj2 player$).ab,ti. | 13 |
| 43 | (iphone$ or i-phone$).ab,ti. | 195 |
| 44 | (ipod$ or i-pod$).ab,ti. | 290 |
| 45 | (podcast$ or pod-cast$).ab,ti. | 223 |
| 46 | Medical informatics/ or medical informatics applications/ | 10210 |
| 47 | audiovisual aids/ | 6223 |
| 48 | Multimedia/ | 1503 |
| 49 | Public health informatics/ | 1044 |
| 50 | User-computer interface/ | 28356 |
| 51 | Interactive tutorial/ | 221 |
| 52 | ((mobile adj health) not van$ not unit$).ab,ti. | 281 |
| 53 | (mhealth or m-health).ab,ti. | 232 |
| 54 | (elearning or e-learning).ab,ti. | 1046 |
| 55 | Electronic mail/ | 1888 |
| 56 | (electronic adj mail$).ab,ti. | 639 |
| 57 | (electronic adj2 messag$).ab,ti. | 147 |
| 58 | (email$ or e-mail$).ab,ti. | 6967 |
| 59 | Hypermedia/ | 393 |
| 60 | Video games/ | 2259 |
| 61 | (computer adj2 gam$).ab,ti. | 945 |
| 62 | (video adj2 gam$).ab,ti. | 1506 |
| 63 | (electronic adj2 gam$).ab,ti. | 185 |
| 64 | (playstation adj1 portable).ab,ti. | 0 |
| 65 | (Sony adj1 PSP).ab,ti. | 1 |
| 66 | (gameboy adj (advance or micro)).ab,ti. | 1 |
| 67 | Nintendo DS$.ab,ti. | 7 |
| 68 | Nintendo DS$.ab,ti. | 7 |
| 69 | Gizmando.ab,ti. | 0 |
| 70 | (Tapwave adj zodiac).ab,ti. | 0 |
| 71 | Video recording/ | 17397 |
| 72 | (video or videos).ab,ti. | 48881 |
| 73 | computer graphics/ | 11994 |
| 74 | Internet/ | 50192 |
| 75 | internet.ab,ti. | 25872 |
| 76 | ("world wide web" or world-wide-web or "world-wide web" or "worldwide web" or website$ or web-site$).ab,ti. | 17178 |
| 77 | (WAP or "wireless application protocol").ab,ti. | 528 |
| 78 | online.ab,ti. | 33421 |
| 79 | on-line.ti,ab. | 18633 |
| 80 | web?based.ab,ti. | 14 |
| 81 | web-based.ab,ti. | 12968 |
| 82 | bluetooth.ab,ti. | 253 |
| 83 | (web adj2 technolog$).ab,ti. | 687 |
| 84 | (chat?room$ or chat-room).ab,ti. | 129 |
| 85 | (blog$ or web-log$ or weblog$).ab,ti. | 662 |
| 86 | Blogging/ | 545 |
| 87 | (bulletin adj board$).ab,ti. | 281 |
| 88 | (message adj board$).ab,ti. | 99 |
| 89 | (interactive adj3 health adj3 communicat$).ab,ti. | 61 |
| 90 | (interactive adj2 televis$).ab,ti. | 92 |
| 91 | (interactive adj2 TV).ab,ti. | 15 |
| 92 | (interactive adj2 technolog$).ab,ti. | 242 |
| 93 | (interactive adj2 multimedia).ab,ti. | 306 |
| 94 | (interactive adj2 software).ab,ti. | 283 |
| 95 | (e-health$ or ehealth$).ab,ti. | 1585 |
| 96 | (electronic adj health).ab,ti. | 4079 |
| 97 | (consumer adj1 health adj1 informatic$).ab,ti. | 73 |
| 98 | (virtual adj reality).ab,ti. | 3989 |
| 99 | (virtual adj learning).ab,ti. | 160 |
| 100 | (surf adj3 web$).ab,ti. | 7 |
| 101 | (surfing adj3 web$).ab,ti. | 34 |
| 102 | or/1-101 | 238781 |
| 103 | Randomized Controlled Trial.pt. | 385157 |
| 104 | randomized controlled trial/ | 385157 |
| 105 | Random Allocation/ | 81824 |
| 106 | Double Blind Method/ | 128786 |
| 107 | Single Blind Method/ | 19799 |
| 108 | clinical trial/ | 493952 |
| 109 | clinical trial, phase i.pt. | 14680 |
| 110 | clinical trial, phase ii.pt. | 23593 |
| 111 | clinical trial, phase iii.pt. | 9403 |
| 112 | clinical trial, phase iv.pt. | 969 |
| 113 | controlled clinical trial.pt. | 89610 |
| 114 | randomized controlled trial.pt. | 385157 |
| 115 | multicenter study.pt. | 178957 |
| 116 | clinical trial.pt. | 493952 |
| 117 | exp Clinical Trials as topic/ | 285207 |
| 118 | or/103-117 | 1052586 |
| 119 | (clinical adj trial$).tw. | 204764 |
| 120 | ((singl$ or doubl$ or treb$ or tripl$) adj (blind$3 or mask$3)).tw. | 126314 |
| 121 | PLACEBOS/ | 33277 |
| 122 | placebo$.tw. | 154654 |
| 123 | randomly allocated.tw. | 16066 |
| 124 | (allocated adj2 random$).tw. | 18570 |
| 125 | or/119-124 | 405044 |
| 126 | 118 or 125 | 1175997 |
| 127 | case report.tw. | 185614 |
| 128 | letter/ | 822486 |
| 129 | historical article/ | 305592 |
| 130 | or/127-129 | 1302266 |
| 131 | 126 not 130 | 1146137 |
| 132 | exp "Tobacco Use"/ or exp "Tobacco Use Disorder"/ or exp Tobacco/ or exp Tobacco, Smokeless/ | 153553 |
| 133 | exp "Tobacco Use Cessation"/ | 21286 |
| 134 | exp Smoking Cessation/ | 20595 |
| 135 | smoking/ | 121774 |
| 136 | (tobacco or smoking or smoke or cigar$ or cigarette$ or snuff).ab,ti. | 202176 |
| 137 | or/132-136 | 243533 |
| 138 | exp alcohol abuse/ or (alcohol-related disorders or alcohol-induced disorders or sobriety).sh. | 70504 |
| 139 | (alcoholi$ or (alcohol and (abstinence or detoxification or intoxicat$ or rehabilit$ or withdraw$))).ab,ti. | 73993 |
| 140 | (drinker$1 or (drink$ adj2 use$1) or ((alcohol$ or drink$) adj3 (abstinen$ or abstain$ or abus$ or addict$ or attenuat$ or binge$ or crav$ or dependen$ or detox$ or disease$ or disorder$ or excessiv$ or harm$ or hazard$ or heavy or high risk or intoxicat$ or misus$ or overdos$ or (over adj dos$) or problem$ or rehab$ or reliance or reliant or relaps$ or withdraw$))).ti,ab. | 72582 |
| 141 | (control$ adj2 drink$).tw. | 1315 |
| 142 | sobriet$.ti,ab,hw. | 726 |
| 143 | or/138-142 | 136689 |
| 144 | (health$ adj2 (diet$ or eating or food$)).ti,ab. | 12234 |
| 145 | (unhealth$ adj2 (diet$ or eating or food$)).ti,ab. | 1415 |
| 146 | ((fruit$ or vegetable$ or salt or sugar$ or fat$ or fiber$ or fibre$) adj (eat$ or intake or consum$ or increas$ or reduc$ or decreas$ or discourage$ or limit$ or lessen or eat$ less or portion$ or serving$ or frequenc$ or number$ or preference$ or choice$)).ti,ab. | 24919 |
| 147 | " five a day ".ti,ab. | 33 |
| 148 | " 5 a day ".ti,ab. | 145 |
| 149 | ((food or diet$) adj (choice$ or frequenc$ or intake)).ti,ab. | 53499 |
| 150 | food preferences/ | 10246 |
| 151 | diet/ | 110849 |
| 152 | obesity/ or overnutrition/ | 132225 |
| 153 | or/144-152 | 299707 |
| 154 | Motor Activity/ or Sedentary Lifestyle/ | 81681 |
| 155 | exercise/ or running/ or jogging/ or swimming/ or walking/ | 110654 |
| 156 | Physical Exertion/ | 53199 |
| 157 | Physical Fitness/ | 22249 |
| 158 | Exercise Therapy/ | 26587 |
| 159 | ((physical$ or aerobic or motor) adj2 (activit$ or training)).tw. | 78449 |
| 160 | (sport$3 or exertion$1 or walk$3 or bicycl$3 or bike$1 or biking or cyclist$ or (exercis$3 adj5 aerobic$1) or rollerblading or rollerskating or skat$ or athletics or baseball or basketball or boxing or football$ or soccer or golf$ or gymnastics or aerobics or recreation or playground$ or "martial arts" or hockey or racquet$ or swimm$ or volleyball or runn$ or jog$ or yoga or pilates or weightlifting or wrestling or tennis or gardening or recreation$1 or dancing or dance).ti,ab. | 208070 |
| 161 | or/154-160 | 433658 |
| 162 | Cardiovascular Diseases/ | 102245 |
| 163 | exp coronary disease/ | 184497 |
| 164 | Hypertension/ | 194494 |
| 165 | exp arteriosclerosis/ | 132943 |
| 166 | exp hyperlipidemia/ | 56672 |
| 167 | (cardiovascular adj3 disease$).ab,ti. | 98826 |
| 168 | (cardiovascular adj3 (fit or fitness)).ab,ti. | 1085 |
| 169 | (coronary adj3 disease$).ab,ti. | 106743 |
| 170 | heart disease$.ab,ti. | 119860 |
| 171 | hypertension.ab,ti. | 266512 |
| 172 | hyperlipid?emia.ab,ti. | 17541 |
| 173 | cholesterol.ab,ti. | 173660 |
| 174 | atherosclerosis.ab,ti. | 78463 |
| 175 | arteriosclerosis.ab,ti. | 9837 |
| 176 | coronary risk factor$.ab,ti. | 3015 |
| 177 | multiple risk factor$.ab,ti. | 2893 |
| 178 | cardiovascular risk factor$.ab,ti. | 21628 |
| 179 | or/162-178 | 932811 |
| 180 | exp Hypertension/ | 212719 |
| 181 | hypertens*.ab,ti. | 306990 |
| 182 | ((high* or raised or raise or elevate or elevated or elevating or heighten* or increas*) adj3 ((blood adj pressure) or (diastolic adj pressure) or (systolic adj pressure) or (pulse adj pressure))).ab,ti. | 45723 |
| 183 | exp Cardiovascular Diseases/pc | 155464 |
| 184 | ((borderline or pre-disease* or pre-clinical* or preclinical* or sub-clinical* or subclinical* or pre-morbid* or premorbid* or risk* or susceptib* or pre-dispos* or predispos* or predict* or probabilit* or likelihood or likeliness or prevent*) adj3 (cardiovascular or cardiometabolic* or cardio-metabolic* or coronary disease* or heart disease* or heart attack* or heart failure or myocardial infarction* or coronary artery disease* or CVD or peripheral artery disease* or PAD or CHD or CAD or arteriosclerosis or atherosclerosis or stroke)).ab,ti. | 141987 |
| 185 | ((high* or raised or raise or elevate or elevated or elevating or heighten* or increas*) adj3 (BP or DBP or SBP)).ab,ti. | 12050 |
| 186 | or/180-185 | 600848 |
| 187 | exp Diabetes Mellitus/ | 318895 |
| 188 | (diabet* or glucose or hyperglycaemi$ or hyperglycaemi$ or postprandial or post-prandial or insulin or hypoglycemi$ or hypoglycaemi$ or IGT or OGTT or impaired glucose tolerance or oral glucose tolerance test or DM or NIDDM).ab,ti. | 758048 |
| 189 | or/187-188 | 799516 |
| 190 | exp neoplasm/ | 2595300 |
| 191 | (neoplasm$ or cancer$ or carcinoma$ or tumo?r$).ti,ab. | 1980113 |
| 192 | or/190-191 | 2953011 |
| 193 | exp Respiratory Tract Diseases/ | 1070737 |
| 194 | ventilatory function.mp. | 1266 |
| 195 | pulmonary symptoms.mp. | 1350 |
| 196 | COPD.mp. | 24262 |
| 197 | exp LUNG EMPHYSEMA/ or exp EMPHYSEMA/ | 10916 |
| 198 | exp Asthma/ | 107190 |
| 199 | or/193-198 | 1076511 |
| 200 | 137 or 143 or 153 or 161 or 179 or 186 or 189 or 192 or 199 | 5975051 |
| 201 | 102 and 131 and 200 | 7303 |
| 202 | limit 201 to yr="2010 -Current" | 3447 |
|  |  |  |
|  |  |  |
