# Supplementary material for: The effectiveness of smoking cessation, physical activity/diet and alcohol reduction interventions delivered by mobile phones for the prevention of non-communicable diseases: A systematic review of randomised controlled trials
Source: PLoS One. 2018 Jan 5;13(1):e0189801. doi: 10.1371/journal.pone.0189801 (PMC5755775; doi:10.1371/journal.pone.0189801)
Supplement: S2 Text — (DOCX) [file pone.0189801.s002.docx]

The effectiveness of mobile phones for the prevention and management of non-communicable diseases: a systematic review protocol

Caroline Free, Jennifer Sutherland^§^

Clinical Trials Unit,

Department of Population Health,

London School of Hygiene and Tropical Medicine

WC1E 7HT

UK

^§^ Corresponding author:

Email addresses:

CF: [caroline.free@lshtm.ac.uk](mailto:caroline.free@lshtm.ac.uk)

JS: [Jennifer.sutherland@lshtm.ac.uk](mailto:Jennifer.sutherland@lshtm.ac.uk)

Abstract: 416 words

Manuscript: 2760 wordsAbstract

Background

The application of mobile information and communication technology (mobile ICT) is rapidly expanding in the fields of health care and public health. This systematic review will summarise the evidence for the effectiveness of mobile ICTs, specifically mobile phone interventions, for the prevention and management of non-communicable diseases (NCDs) and their risk factors around the world.

Methods and Design

To be included in the review interventions must employ mobile phone technologies and aim to prevent or manage NCDs (hypertension, cardiovascular disease/s, cancer, respiratory disease/s, or diabetes) and/or their risk factors (tobacco use, harmful alcohol use, physical inactivity, unhealthy diet). The review will include: (1) interventions delivered to health care professionals designed to improve diagnosis, investigation, treatment, monitoring and management of these diseases and their risk factors; (2) interventions delivered to health care consumers designed to enhance the self-management of NCDs, alter NCD risk behaviours or improve treatment compliance.

We will include trials identified in our previous comprehensive systematic review that targeted NCDs or their risk factors which included trials published between 1990 and September 2010. A comprehensive, electronic search strategy will be used to identify randomised controlled studies, published since September 2010 and indexed in MEDLINE, EMBASE, Global Health and CINAHL. The search strategy was initially devised to include terms for the following mobile information and communication technologies and a range of compatible media: mobile phone; personal digital assistant (PDA); handheld computer (e.g. tablet PC); PDA phone (e.g. BlackBerry, Palm Pilot); Smartphone; enterprise digital assistant; portable media player (i.e. MP3 or MP4 player); handheld video game console. These terms will then be combined with search terms and synonyms related to the specified NCDs and their risk factors. Although the search strategy includes all mobile ICTs, due to the large number of trials identified and limited funding and time to conduct the review the protocol was amended to include only interventions delivered by mobile phone and Smartphone. Bibliographies of primary studies and review articles meeting the inclusion criteria will be searched manually to identify further eligible studies. Data on objective and self-reported outcomes and study quality will be independently extracted by two review authors. Where there are sufficient numbers of similar interventions, we will calculate and report pooled risk ratios or standardised mean differences using meta-analysis.

Discussion

This systematic review will provide recommendations on the use of mobile computing and communication technology delivered via mobile phone for the prevention and management of NCDs and will guide future work on intervention development and primary research in this field.

Background

Non-communicable diseases (NCDs), including cardiovascular diseases, diabetes, cancers and chronic respiratory diseases, are the leading cause of death and disability worldwide, responsible for two thirds of global deaths each year and 54 percent of healthy life-years lost, as measured by Disability-Adjusted Life Years (126, 127). Morbidity and premature deaths from NCDs pose substantial economic costs through increased health care spending and reduced economic productivity; estimates suggest that NCDs will result in economic losses of US$7 trillion over the next 15 years(126). NCDs are largely preventable through the reduction of a set of common behavioural risk factors, including tobacco use, harmful alcohol consumption, sedentary lifestyles, and unhealthy diets high in fat, sugar and salt, and low in fibre, fruits and vegetables. Interventions to eliminate these behavioural risk factors could have a significant impact on the burden of NCDs through a reduction in intermediate risk factors such as overweight and obesity, hypertension, raised blood lipids and raised blood glucose levels (128, 129). Alternatively, diagnosis, treatment and chronic care for NCDs and their intermediate risk factors can also significantly reduce morbidity and mortality.

During the 2011 United Nations High Level Meeting on Non-Communicable Diseases, the use of mobile computing and communication technologies in health care and public health (M-health) was highlighted as a key strategy to combat NCDs(130). In 2012, the UN Information and Communication Technologies agency (ITU) and the World Health Organization together launched the “mHealth” initiative to use mobile technology to help combat NCDs (131).

M-health programmes and interventions use mobile information and communication technologies (mobile ICTs) such as mobile phones and Smartphones, for a range of functions from clinical decision support systems and data collection tools for healthcare professionals(132, 133), to supporting health behaviour change and chronic disease management by patients in the community(134). M-health is a rapidly expanding area of research and practice. Current documented M-health interventions and programmes for the prevention and management of NCDs include mobile phone text messaging to support management of diabetes, hypertension, and asthma, and mobile phone text messaging and PDAs as aids to smoking cessation, body weight loss and reducing alcohol consumption (134-137). Whilst the majority of M-health interventions are reported from high income countries, there is an emerging literature on the application of mobile ICTs in low-income countries (132, 138-141).

Mobile communication technology is the fastest growing sector of the communications industry in low-income countries(142, 143). In 2013, there were 6.8 billion mobile ICT (phone) subscriptions, which is almost as many subscriptions as there are people in the world (7.1 billion) (144). Mobile ICT networks provide geographical coverage that has the potential to reach up to 90% of the world’s population (145). In the last two decades, the global digital divide has narrowed most for mobile phone use, with many low income countries “leap-frogging” over fixed-line communications technologies, straight to expansion of wireless cellular communication networks (143, 146, 147). Whilst wireless communication network coverage and mobile phone ownership are not universal, or equally distributed in low income countries, (146, 148), there is still huge potential for M-health interventions and programmes to have positive effects on health outcomes in resource-poor settings(140).

Mobile technologies have a number of key features that give them an advantage over other information and communication technologies in particular activities within health care and public health. Firstly, many mobile ICTs have wireless cellular communication capability, providing the potential for continuous, interactive communication from any location e.g. telephone calls, text and multimedia messaging and also internet access via Wireless Application Protocol (WAP) or mobile broadband internet. Secondly, the devices are portable because of their small size, low weight and rechargeable, long-life battery power. Finally, many mobile ICTs have sufficient computing power to support multimedia software applications. The combination of these features varies between specific devices and their relative importance will change with the health activity in which they are used. However, with advances in technology development, single devices increasingly possess many or all of these functions.

A comprehensive systematic review in 2013 included randomised controlled trials for all mobile technology interventions and all health outcomes. The review found mixed evidence regarding the benefit of interventions delivered to healthcare consumers, and nearly all included trials were conducted in high-income countries (149). The review also found that some interventions delivered to health care professionals via mobile ICT to improve health-care service delivery processes were modestly effective. The review highlighted the need for more trials of these interventions (117). However, the field is rapidly expanding and an updated review focussing on NCDs and their risk factors is needed.

In this systematic review, we propose to identify trials of interventions delivered by mobile phone technologies for the prevention and management of NCDs and their risk factors.

The objectives of this review are to: (1) Describe current interventions evaluated by randomised controlled trial for the prevention and management of NCDs and their risk factors. 2) Assess the effectiveness of interventions delivered by mobile phones for the prevention and management of NCDs and their risk factors. This will provide a broad overview of the potential role of mobile phones in reducing the burden of NCDs. WHO will use the review findings to (1) share experiences and good practices of using M-health for effective public health for NCDs; and (2) help shape country programmes for the implementation of M-health for NCDs.

Methods and Design

Review Inclusion Criteria

*Types of technology*

For the purposes of this review, mobile ICTs will be defined as devices which either have interactive wireless cellular communication capability and/or those which run software applications and are highly portable. We will include interventions using mobile phones, Smartphones (e.g. the iphone) and PDA phones. A summary of the functions available with each of these devices is provided in Table 1. For the purposes of this review desktop personal computers, notebook (laptop) computers, subnotebook computers, netbooks, pagers, handheld calculators and pedometers are not considered to be mobile ICTs and interventions delivered exclusively on these devices will be excluded.

*Types of intervention*

Interventions must aim to prevent or manage NCDs and their risk factors, employing any mobile phone or Smartphone technology. The review will consider the prevention and management of hypertension, cardiovascular disease/s, cancer/s, respiratory disease/s, diabetes, tobacco use, harmful alcohol use, physical inactivity, and unhealthy diets. The review will include: (1) interventions delivered to health care professionals designed to improve diagnosis, investigation, treatment, monitoring and management of these diseases and their risk factors; (2) interventions delivered to health care consumers designed to enhance the self-management of NCD, alter NCD risk behaviours or improve treatment compliance.

We will therefore include:

- Any intervention delivered using mobile phones owned or directly used by a patient or lay person;
- Any clinical or practice aid delivered using mobile phones owned or directly used by a healthcare professional;
- Any data collection or storage for the purposes of NCD care or research using mobile phones.

We anticipate that interventions will aim to address one of the health domains shown in Table 1, although it is likely that further mobile phone applications will be identified in the review and this framework will be updated.

*Types of studies*

Studies must have used a randomised controlled design to evaluate a mobile phone intervention. Previous reviews have highlighted the difficulty in assessing the impact of mobile ICTs on health outcomes when the mobile ICT is used as an adjunct to other interventions and services e.g. text messaging in addition to clinician appointments for managing hypertension(135). We will therefore only include studies where the mobile phone is the primary intervention component under evaluation. We will include studies evaluating:

- Interventions delivered via a single mobile ICT to the treatment group (N.B. this could therefore include an intervention where a number of behaviour change techniques are used, e.g. prompts, reminders and patient-initiated support, but all delivered through a single mobile ICT, e.g. SMS to and from a mobile phone), where the control group receives no mobile ICT intervention;
- Multi-mobile ICT interventions where the treatment group receives one or more interventions delivered through multiple mobile ICTs, but no interventions through other (non-mobile ICT) modes and the control group receives no mobile ICT interventions;
- Mixed mobile ICT and non-mobile ICT interventions where the treatment and control group both receive all non-mobile ICT components of the intervention and the mobile ICT intervention is delivered only to the treatment group, e.g. SMS plus group counselling for smoking cessation in the treatment group and the control group receives group counselling only;
- Interventions delivered by different mobile ICTs e.g. iphone vs. regular mobile phone;
- The features and components of interventions delivered on a particular mobile ICT such as the intensity, personalisation, content, duration and timing of an intervention, and the degree of software or other customisation.

We will exclude studies evaluating:

- Mixed mobile ICT and non-mobile ICT interventions where the treatment and control group both receive the mobile ICT component;
- Interventions where there are other treatment differences between the treatment and control groups besides the delivery of the mobile ICT component(s).

*Types of participants*

There will be no limits on study participants in terms of age, gender, ethnicity, morbidities (for patients and the general population) or staff role or occupation (for healthcare professionals e.g. nurse, surgeon, or physiotherapist). There will be no limits on study setting and we will include studies at all levels of healthcare and those conducted in the community.

*Types of outcome measures*

All outcome measures reported in studies meeting the inclusion criteria will be extracted, both objective and self-reported measures. User-acceptability will be assessed as a self-reported outcome for all intervention types. We will also seek data on unintended adverse consequences of the interventions and process outcomes (e.g. involvement in road traffic accidents).

Primary outcome measures will include any objective measure of outcomes related to the selected NCDs or their risk factors. These may include biologically confirmed smoking cessation or mean change in body mass index, blood pressure, blood lipids or blood glucose levels. Secondary outcome measures will include: cognitive outcomes relating to knowledge, motivation, self-efficacy and intention; self-reported outcomes related to NCD-related behaviours (e.g. number of cigarettes smoked). Examples of outcome measures for each health domain are provided in Table 2.

Literature search

We will include trials identified in our previous comprehensive systematic review that targeted NCDs or their risk factors using mobile phones and included studies published between 1990 and September 2010. We will use a three-part search strategy to identify studies meeting the inclusion criteria above that have been published since September 2010: (1) we will search electronic bibliographic databases for published work using a comprehensive search strategy for mobile phone technology interventions and NCDs; (2) we will search trial registers for ongoing and recently completed trials; (3) we will search reference lists of primary studies included in the review and the reference lists of relevant, previously published reviews.

*Electronic bibliographic databases*

The following electronic bibliographic databases will be searched: MEDLINE, EMBASE, Global Health and CINAHL, The search strategy will include terms relating to or describing mobile technologies combined with the NCDs and risk factors of interest. The search was initially designed to include all mobile ICTs but due to the larger number of trials identified and limited funding and time to conduct the review the protocol was amended and only interventions delivered by mobile phone were included. The search strategy for MEDLINE is shown in Appendix 2. The search terms will be adapted for use with other bibliographic databases in combination with database-specific filters for randomised controlled trials, where these are available. There will be no language restrictions.

*Trial registers*

Ongoing, recently completed and unpublished clinical trials meeting the inclusion criteria described above will be identified from the following research registers: National Institutes of Health clinical trials registry (US); and Current Controlled Trials (includes the International Standard Randomised Controlled Trial Number Register).

Study screening and selection

Titles and abstracts of studies retrieved using the search strategy and those from additional sources will be screened independently by two review authors to identify studies that potentially meet the inclusion criteria outlined above. The full text of these potentially eligible studies will be retrieved and independently assessed for eligibility by two review authors. Any disagreement between the two review authors over the eligibility of particular studies will be resolved through discussion with a third review author.

Data extraction

A standardised, pre-piloted form will be used to extract data from the included studies for assessment of study quality and evidence synthesis. Extracted information will include: study setting (including country); study population and participant demographics and baseline characteristics; mobile ICT used; details of the intervention and control condition; study methodology; recruitment and study completion rates; outcomes and times of assessment; indicators of acceptability to users; suggested mechanisms of intervention action; information for assessment of the risk of bias (see below).Two review authors will independently extract data, discrepancies will be identified and resolved through discussion (with a third author where necessary).

Assessing risk of bias

Two review authors will independently assess the risk of bias in included studies by considering the following characteristics, as recommended by the International Cochrane Collaboration(150):

- Randomisation sequence generation: was the allocation sequence (used to assign participants to the treatment and control groups) adequately generated? (This criterion only applies to randomised controlled trials.)
- Treatment allocation concealment: was the allocated treatment adequately concealed from study participants and clinicians and other healthcare or research staff at the enrolment stage?
- Blinding: were outcome assessors sufficiently blinded to the intervention allocation throughout the trial?
- Completeness of outcome data: were participant exclusions, attrition and incomplete outcome data adequately addressed in the published report?
- Other sources of bias: was the trial apparently free of any other problems that could produce a high risk of bias?

Disagreements between the review authors over the risk of bias in particular studies will be resolved by discussion, with a third review author where necessary. The level of risk of bias in each of these domains will be presented separately for each study in tables in the final review publication.

Analysis

*Descriptive analysis*

We will provide a narrative synthesis of the findings from the included studies. We will structure the narrative synthesis by describing the studies according to the following characteristics:

- The type of intervention e.g. individual behaviour change, chronic disease self-management, clinic appointment reminders or clinical diagnostic aid;
- The type of mobile phone used i.e. Smartphone;
- The target population characteristics e.g. age, gender, ethnicity, socioeconomic status and/or education level, low/middle/high income country setting (classified according to the World Bank List of Economies(151));
- The type of outcome e.g. smoking cessation or weight loss;
- Intervention content - features of the mobile phone employed (e.g. SMS, video), intervention components such as reminders, feedback or peer support, intensity, duration, personalisation and theoretical basis (if stated).

We will provide summaries of intervention effects for each study by calculating risk ratios (for dichotomous outcomes) or standardised mean differences (for continuous outcomes) from the data presented in the published studies or obtained from study authors.

*Statistical analysis*

Where studies have used the same type of intervention and mobile phone with the same outcome measure we will use Stata v11.0 (152) to pool the results of randomised controlled trials using a random-effects meta-analysis, with standardised mean differences for continuous outcomes and risk ratios for binary outcomes, and calculate 95% confidence intervals and two sided P values for each outcome. In studies where the effects of clustering have not been taken into account, we will adjust the standard deviations for the design effect, using intra-class coefficients, if they are provided in the study reports, or alternatively using external estimates obtained from similar studies(153). Heterogeneity between the studies in effect measures will be assessed using both the χ^2^ test and the *I*^2^ statistic. We will consider an *I*^2^ value greater than 50% indicative of substantial heterogeneity. We will conduct sensitivity analyses based on study quality (risk of bias; level of participant drop-out) in order to investigate possible sources of heterogeneity). We will assess evidence of publication bias using Egger's weighted regression method for continuous outcomes and Begg’s rank correlation test for dichotomous outcomes.

Conclusion

This systematic review will provide a detailed summary of the evidence for the effectiveness of mobile phone technologies for the prevention and management of NCDs and their risk factors.

Competing Interests

The authors declare that they have no competing interests.

Authors Contributions

CF and JS designed the study drawing on the design of our comprehensive systematic review.

Acknowledgements

This work is funded by the World Health Organization.

References

1. World Health Organization, *Global action plan for the prevention and control of noncommunicable diseases 2013-2020*. 2013: Geneva, Switzerland.

2. Murray, C.J.L. and A.D. Lopez, *Measuring the Global Burden of Disease.* New England Journal of Medicine, 2013. **369**(5): p. 448-457.

3. World Health Organization, *2008-2013 action plan for the global strategy for the prevention and control of noncommunicable diseases: prevent and control cardiovascular diseases, cancers, chronic respiratory diseases and diabetes*. 2008: Geneva.

4. World Health Organization, *Global status report on noncommunicable diseases 2010: Description of the global burden of NCDs, their risk factors and determinants*. 2011, World Health Organization.

5. World Health Organization, *Zero Draft: Global Action Plan for the Prevention and Control of Noncommunicable diseases 2013-2020. Geneva: WHO; 2012*. 2012, WHO: Geneva.

6. World Health Organization. *ITU and WHO launch mHealth initiative to combat noncommunicable diseases*. 212 [cited 2014 04 August]; Available from: <http://www.who.int/mediacentre/news/releases/2012/mHealth_20121017/en/>.

7. Blaya, J.A., H.S. Fraser, and B. Holt, *E-health technologies show promise in developing countries.* Health Aff (Millwood), 2010. **29**(2): p. 244-51.

8. Lindquist, A.M., et al., *The use of the Personal Digital Assistant (PDA) among personnel and students in health care: a review.* Journal of Medical Internet Research, 2008. **10**(4): p. e31.

9. Cole-Lewis, H. and T. Kershaw, *Text Messaging as a Tool for Behavior Change in Disease Prevention and Management.* Epidemiol Rev, 2010.

10. Fjeldsoe, B.S., A.L. Marshall, and Y.D. Miller *Behavior change interventions delivered by mobile telephone short-message service (Structured abstract)*. American Journal of Preventive Medicine, 2009. 165-173.

11. Heron, K.E. and J.M. Smyth, *Ecological momentary interventions: Incorporating mobile technology into psychosocial and health behaviour treatments.* Br J Health Psychol, 2010. **15**(Pt 1): p. 1-39.

12. Krishna, S., S.A. Boren, and E.A. Balas, *Healthcare via cell phones: a systematic review.* Telemedicine Journal & E-Health, 2009. **15**(3): p. 231-40.

13. Curioso, W.H. and P.N. Mechael, *Enhancing 'M-health' with south-to-south collaborations.* Health Aff (Millwood), 2010. **29**(2): p. 264-7.

14. Leach-Lemens, C., *Using mobile phones in HIV care and prevention.* HIV & AIDS Treatment in Practice, 2009. **137**: p. 7.

15. Consulting, V.W., *mHealth for Development: The Opportunity of Mobile Technology for Healthcare in the Developing World.* . 2009: Washington D.C. and Berkshire, UK.

16. Mechael, P.N., et al., *Barriers and Gaps Affecting mHealth in Low and Middle Income Countries: Policy White Paper*. 2010, Center for Global Health and Economic Development, Earth Institute, Columbia University.

17. ITU, *Mobile overtakes fixed: Implications for policy and regulation*. 2003, ITU International Telecommunications Union.

18. Orbicom-ITU, *From the digital divide to digital opportunities: measuring infostates for development*, G. Sciadas, Editor. 2005: Canada.

19. International Telcommunication Union, *Measuring the Information Society*. 2012, International Telecommunications Union: Geneva.

20. International Telcommunication Union. *The world in 2013: ICT facts anf Figures*. 2013; Available from: [www.itu.int](http://www.itu.int).

21. Banks, K. and R. Burge, *Mobile Phones: An Appropriate Tool for Conservation and Development?* 2004: Cambridge, UK.

22. Hamilton, J., *Are main lines and mobile phones substitutes or complements? Evidence from Africa.* Telecommunications Policy, 2003. **27**(1-2): p. 109-133.

23. Donner, J., *Research Approaches to Mobile Use in the Developing World: A Review of the Literature.* Information Society, 2008. **24**(3): p. 140-159.

24. Free, C., et al., *The effectiveness of mobile-health technology-based health behaviour change or disease management interventions for health care consumers: a systematic review.* PLoS Medicine / Public Library of Science, 2013. **10**(1): p. e1001362.

25. Free, C., et al., *The effectiveness of mobile-health technologies to improve health care service delivery processes: a systematic review and meta-analysis.* PLoS Medicine / Public Library of Science, 2013. **10**(1): p. e1001363.

26. Higgins, J.P.T. and S. Green, *Cochrane Handbook for Systematic Reviews of Interventions Version 5.0.0*. 2008, The Cochrane Collaboration.

27. Gomez, R.G., *The pertinence of revolution.* Review of International Studies, 2001. **27**(4): p. 675-681.

28. StataCorp, *Stata Statistical Software: Release 11*. 2009, StataCorp LP.: College Station, TX.

29. Ukoumunne, O.C., et al., *Methods for evaluating area-wide and organisation-based interventions in health and health care: a systematic review.* Health Technol Assess, 1999. **3**(5): p. iii-92.

**Table 1**. Functions of mobile information and communication technologies included in the review. Brackets indicate where functions are available on only some higher specification models.

|  | Mode of communication | | | | | | | | |
| --- | --- | --- | --- | --- | --- | --- | --- | --- | --- |
|  | Voice | SMS | MMS | Email | WAP internet | Wireless cellular broadband | Audio | Video | Custom/additional software support |
| Mobile phone |  |  |  |  |  |  |  |  |  |
| Basic model  (e.g. Nokia 1280)^1^ | ✓ | ✓ |  |  |  |  |  |  |  |
| High-end model  (e.g. Nokia 6303i)^1^ | ✓ | ✓ | ✓ | ✓ | ✓ |  | ✓ | ✓ |  |
| Smartphone | ✓ | ✓ | ✓ | ✓ |  | ✓ | ✓ | ✓ |  |
| PDA phone | ✓ | ✓ |  | ✓ | ✓ |  |  |  |  |
|  |  |  |  |  |  |  |  |  |  |

Abbreviations: MMS, multimedia messaging service; PDA, personal digital assistant; SMS, short messaging service; WAP, wireless application protocol.

^1^ Source GSM Arena (<http://www.gsmarena.com/>, accessed 09.06.10)

## Table 2. Example outcome measures for anticipated mobile phone-based interventions.

| **Mobile phone-based intervention** | **Example outcome measures** | |
| --- | --- | --- |
|  | **Objective outcomes** | **Self-reported outcomes** |
|  |  |  |
| Clinical decision support systems – diagnosis | - Adherence to clinical protocol - Diagnosis of disease or disease risk (primary prevention) - Treatment e.g. medication prescribed | - Use of clinical protocol - Ease of clinical protocol use/ comprehensibility |
| Clinical decision support systems - disease management | - Successful disease management e.g. diabetes control measured by HbA1C, peak flow - Medication prescribed - Investigations arranged - Health outcomes e.g, cardiac events | - Use of clinical protocol - Ease of clinical protocol use/ comprehensibility |
| Test result notification | - Time to result notification - Time to treatment initiation | - Patient satisfaction with clinic service - Clinician preference for mode of notification |
| Appointment reminders | - Percent of appointments missed - Percentage of appointments cancelled in advance | - Patient intention to attend appointment |
| Treatment programmes | - Treatment outcomes | - Patient satisfaction with treatment - Perceived level of support - Perceived changes in health status/disease condition |
| NCD management  Medication adherence | - Disease management e.g. diabetes control measured by HbA1C or asthma management expiration peak flow rate - Percent of medication doses taken on time | - Self-efficacy to manage condition/medication |
| Health behaviour change | - Health outcome e.g. body mass index (weight loss) or blood cotinine levels (smoking cessation) | - Self-efficacy to increase exercise/control dietary intake - Self reported behaviour |
