# Supplementary material for: The effectiveness of smoking cessation, physical activity/diet and alcohol reduction interventions delivered by mobile phones for the prevention of non-communicable diseases: A systematic review of randomised controlled trials
Source: PLoS One. 2018 Jan 5;13(1):e0189801. doi: 10.1371/journal.pone.0189801 (PMC5755775; doi:10.1371/journal.pone.0189801)
Supplement: S1 Table — (DOCX) [file pone.0189801.s004.docx]

**Table 1: Risk of Bias - Smoking cessation trials**

| **Author** | **Randomisation/ allocation sequence generation** | **Treatment allocation concealment** | **Blinding** | **Incomplete outcome data** | **Selective outcome reporting** | **Other bias** |
| --- | --- | --- | --- | --- | --- | --- |
|  |  |  |  |  |  |  |
| **Smoking** | | | | | | |
| **ABROMS (2014)** | L | L | L | L | U | L |
| **BORLAND (2013)** | L | H | H | H | L | L |
| **BULLER (2014)** | L | L | U | H | H | L |
| **CHAN (2015)** | H | H | U | H | L | L |
| **CHOW (2015)** | L | L | L | L | L | L |
| **FREE (2009)** | L | L | L | L | L | L |
| **FREE (2011)** | L | L | L | L | L | L |
| **GOLSHAHI (2015)** | U | U | H | U | U | U |
| **GRITZ (2013)** | L | U | U | H | H | L |
| **HAUG (2009)** | L | L | H | H | L | L |
| **MCDANIEL (2015)** | L | L | H | H | U | L |
| **PENG (2013)** | L | L | L | H | L | L |
| **POLLAK (2013)** | L | U | U | L | L | H |
| **RODGERS (2005)** | L | L | L | H | L | L |
| **SHI (2013)** | L | U | U | H | L | H |
| **SKOV-ETTRUP (2014)** | U | U | U | H | H | H |
| **VIDRINE (2006)** | L | U | H | L | H | L |
| **WHITTAKER (2011)** | L | U | H | H | L | H |
| **YBARRA (2012)** | H | L | H | H | H | H |
| **YBARRA (2013)** | L | H | H | H | L | H |

**Table 2: Risk of Bias - diet/physical activity trials**

| **Author** | **Randomisation/ allocation sequence generation** | **Treatment allocation concealment** | **Blinding** | | **Incomplete outcome data** | **Selective outcome reporting** | **Other bias** |
| --- | --- | --- | --- | --- | --- | --- | --- |
|  |  |  |  |  | |  |  |
| **Physical activity** | | | | | | | |
| **CADMUS-BERTRAM (2015)** | L | U | L | L | | U | U |
| **DIREITO (2015)** | L | L | H | L | | L | U |
| **GLYNN (2014)** | L | L | L | H | | L | L |
| **KIM (2013)** | L | U | H | L | | L | H |
| **LIU (2008)** | L | U | U | U | | H | L |
| **MADDISON (2014)** | L | L | L | L | | L | L |
| **MARTIN (2015)** | L | L | L | L | | L | U |
| **NEWTON (2009)** | L | L | H | L | | U | U |
| **NGUYEN (2009)** | U | L | L | L | | L | L |
| **PETRELLA (2014)** | H | H | H | H | | L | U |
| **PRESTWICH (2010)** | L | L | L | L | | U | L |
| **PRESTWICH (2009)** | L | H | H | U | | H | L |
| **SIRRIYEH (2010)** | L | L | L | L | | L | U |
| **VAN DER WEEGEN (2015)** | U | H | L | H | | L | L |
| **WANG (2015)** | U | U | L | L | | L | U |
|  |  |  |  |  | |  |  |
| **Physical activity and diet** | | | | | | | |
| **ALLEN (2013)** | U | U | U | H | | L | L |
| **BRINDAL (2013)** | L | U | U | L | | H | H |
| **CARTER (2013)** | L | U | L | H | | L | H |
| **COWDERY (2015)** | L | U | H | L | | U | U |
| **DE NIET (2012)** | L | L | U | H | | L | L |
| **VAN DROGELEN (2014)** | U | U | H | H | | L | U |
| **FASSNACHT (2015)** | U | U | H | U | | U | U |
| **FILION (2015)** | L | H | H | H | | U | H |
| **HAAPALA (2009)** | H | U | U | H | | U | L |
| **HEBDEN (2015)** | L | U | U | L | | L | L |
| **KIM (2015)** | L | H | H | L | | L | L |
| **LAING (2014)** | L | L | L | H | | U | L |
| **LIN (2015)** | U | U | L | H | | U | L |
| **MARTIN (2015B)** | L | U | L | L | | U | U |
| **NAPOLITANO (2013)** | L | U | U | L | | H | L |
| **PATRICK (2013)** | U | U | U | L | | L | H |
| **RAMACHANDRAN (2013)** | L | L | U | L | | L | L |
| **SHAHID (2015)** | U | H | H | U | | U | U |
| **SHAPIRO (2012)** | L | L | H | H | | L | U |
| **SHAPIRO (2008)** | L | L | H | H | | U | L |
| **SHAW (2013)** | L | U | U | U | | L | U |
| **STEINBERG (2013)** | L | U | U | U | | L | L |
| **SVETKEY (2015)** | U | U | U | L | | U | U |
| **TURNER-MCGRIEVY (2011)** | L | H | H | U | | U | L |
| **VARNFIELD (2014)** | L | H | H | H | | H | U |
| **WONG (2013)** | L | L | H | L | | L | H |
|  |  |  |  |  | |  |  |
| **Diet** | | | | | | | |
| **MORIKAWA (2011)** | H | U | U | U | | H | L |
| **SOURETI (2011)** | L | L | H | L | | H | H |
| **WHARTON (2014)** | H | U | U | H | | U | U |

**Table 3: Risk of Bias – physical activity/diet/smoking**

| **Author** | **Randomisation/ allocation sequence generation** | **Treatment allocation concealment** | **Blinding** | **Incomplete outcome data** | **Selective outcome reporting** | **Other bias** |
| --- | --- | --- | --- | --- | --- | --- |
| **Physical activity, diet, and smoking** | | | | | | |
| **CHOW (2015)** | L | L | L | L | L | L |
| **GOLSHAHI (2015)** | U | U | H | U | U | U |

**Table 4: Risk of Bias - Alcohol**

| **Author** | **Randomisation/ allocation sequence generation** | **Treatment allocation concealment** | **Blinding** | **Incomplete outcome data** | | **Selective outcome reporting** | **Other bias** |
| --- | --- | --- | --- | --- | --- | --- | --- |
|  |  |  |  | |  |  |  |
| **Alcohol** | | | | | | | |
| **AGYAPONG (2013)** | L | U | H | | H | L | L |
| **ANDERSSON (2015)** | L | L | H | | H | L | L |
| **GAJECKI (2014)** | L | U | H | | H | L | H |
| **GUSTAFSON (2014)** | L | L | H | | H | L | L |
| **HAUG (2009)** | L | L | H | | H | L | L |
| **MASON (2014)** | U | U | H | | L | U | H |
| **SUFFOLETTO (2012)** | L | L | U | | U | L | H |
| **SUFFOLETTO (2014)** | L | L | H | | H | L | L |
