# Supplementary material for: The effectiveness of smoking cessation, physical activity/diet and alcohol reduction interventions delivered by mobile phones for the prevention of non-communicable diseases: A systematic review of randomised controlled trials
Source: PLoS One. 2018 Jan 5;13(1):e0189801. doi: 10.1371/journal.pone.0189801 (PMC5755775; doi:10.1371/journal.pone.0189801)
Supplement: S2 Table — (DOCX) [file pone.0189801.s005.docx]

Supplementary Material – secondary outcomes

**Table 1: Secondary outcomes - smoking cessation**

| Clinical Area | Trial | Intervention | Outcome | RR/MD | LCI | UCI |
| --- | --- | --- | --- | --- | --- | --- |
| Smoking | Abroms 2014 (1) | SMS versus quitting guidebook | Self-reported abstinence in past 7 days (6 months) | 1.53 | 1.13 | 2.07 |
|  | Abroms 2014 | SMS versus quitting guidebook | Self-reported abstinence in past 30 days (6 months) | 1.57 | 1.1 | 2.26 |
|  | Abroms 2014 | SMS versus quitting guidebook | Self-reported repeated point prevalence abstinence (not smoking in the past 30 days at 3 and 6 months) | 1.99 | 1.27 | 3.13 |
|  | Borland 2013 (2) | OnQ SMS versus minimal treatment | 7 day point prevalence abstinence (self-reported) – 7 mo | 1.20 | 0.95 | 1.50 |
|  | Borland 2013 | OnQ SMS versus minimal treatment | Continuous abstinence – 6 mo | 1.46 | 0.95 | 2.26 |
|  | Borland 2013 | OnQ SMS versus minimal treatment | No. cessation attempts in past year | 1.05 | 0.93 | 1.18 |
|  | Borland 2013 | OnQ SMS versus minimal treatment | 7 day point prevalence abstinence (self-reported) – 1 mo | 1.42 | 1.09 | 1.84 |
|  | Buller 2014 (3) | REQ-mobile app versus OnQ SMS | Continuous abstinence (self-reported) – 3 mo | 0.57 | 0.26 | 1.24 |
|  | Buller 2014 | REQ-mobile app versus OnQ SMS | Smoking cessation on day of interview – 3 mo | 0.61 | 0.38 | 0.96 |
|  | Buller 2014 | REQ-mobile app versus OnQ SMS | Quit smoking > 1 mo ago -3 mo | 0.82 | 0.66 | 1.01 |
|  | Buller 2014 | REQ-mobile app versus OnQ SMS | Quit smoking < 1 mo ago - 3 mo | 2.14 | 0.95 | 4.81 |
|  | Buller 2014 | REQ-mobile app versus OnQ SMS | Attempted to quit smoking – 3 mo | 0.91 | 0.69 | 1.21 |
|  | Buller 2014 | REQ-mobile app versus OnQ SMS | Open to quitting smoking | 1.02 | -0.22 | 4.82 |
|  | Buller 2014 | REQ-mobile app versus OnQ SMS | Like to quit smoking | 0.65 | 0.11 | 3.75 |
|  | Buller 2014 | REQ-mobile app versus OnQ SMS | Planning to quit smoking | 4.08 | 0.91 | 18.29 |
|  | Buller 2014 | REQ-mobile app versus OnQ SMS | Set a quit smoking date | 4.00 | 1.20 | 13.34 |
|  | Buller 2014 | REQ-mobile app versus OnQ SMS | Successfully quit smoking | 0.65 | 0.48 | 0.87 |
|  | Buller 2014 | REQ-mobile app versus OnQ SMS | No use of NRT |  |  |  |
|  | Buller 2014 | REQ-mobile app versus OnQ SMS | < 1 wk use of NRT | 1.55 | 0.42 | 5.76 |
|  | Buller 2014 | REQ-mobile app versus OnQ SMS | 2 wks use of NRT | 0.46 | 0.14 | 1.56 |
|  | Buller 2014 | REQ-mobile app versus OnQ SMS | >2 wks use of NRT | 0.93 | 0.37 | 2.31 |
|  | Buller 2014 | REQ-mobile app versus OnQ SMS | 30 day point prevalence abstinence (Self-reported) – 3 mo | 0.56 | 0.27 | 1.15 |
|  | Chan 2015 (4) | sms versus self-help booklet | self-reported reduction in daily cigarette consumption 12 months | 1.06 | 0.84 | 1.33 |
|  | Chan 2015 | sms versus self-help booklet | quit attempts with smoking abstinence >= 24h 12 months | 1.00 | 0.83 | 1.20 |
|  | Chan 2015 | sms versus self-help booklet | self-reported 7-day point prevalence (PP) tobacco abstinence at 12-month follow-up | 0.99 | 0.71 | 1.36 |
|  | Free 2009 (5) | 5 SMS/day post quit date versus fortnightly generic SMS | 28 day continuous abstinence (self-reported) – 6 mo | 0.79 | 0.41 | 1.52 |
|  | Free 2009 | 5 SMS/day post quit date versus fortnightly generic SMS | Currently not smoking at follow up (self-reported) – 4 weeks | 2.08 | 1.11 | 3.89 |
|  | Free 2011 (6) | 5 SMS/day post quit date versus fortnightly generic SMS | 7 day point prevalence abstinence - 6 mo | 1.32 | 1.20 | 1.46 |
|  | Free 2011 | 5 SMS/day post quit date versus fortnightly generic SMS | 28 day continuous abstinence – 6 mo | 1.47 | 1.30 | 1.65 |
|  | Free 2011 | 5 SMS/day post quit date versus fortnightly generic SMS | 7 day point prevalence abstinence – 4 wk | 2.37 | 2.12 | 2.66 |
|  | Golshahi 2015 (7) | SMS versus pamphlets | Current smoker at 8 months | 5.00 | 0.25 | 101.31 |
|  | Gritz 2013 (8) | Cell phone counseling versus usual care | 30 day abstinence – 1 year* | 2.20 | 0.83 | 5.83 |
|  | Gritz 2013 | Cell phone counseling versus usual care | 24 hr abstinence – 6 mo* | 2.36 | 1.28 | 4.38 |
|  | Gritz 2013 | Cell phone counseling versus usual care | 7 day point prevalence abstinence – 1 year* | 2.41 | 1.01 | 5.76 |
|  | Haug 2009 (9) | 3 SMS/wk with tailored feedback versus weekly SMS | Any attempt to quit during follow up | 1.23 | 0.83 | 1.82 |
|  | Haug 2009 | 1 SMS/wk with tailored feedback versus weekly SMS | No. cigarettes per day | 0.70 | -1.55 | 2.95 |
|  | Haug 2009 | 3 SMS/wk with tailored feedback versus weekly SMS | No. cigarettes per day | 0.20 | -1.91 | 2.31 |
|  | Haug 2009 | 1 SMS/wk with tailored feedback versus weekly SMS | Self-efficacy | 0.00 | -0.30 | 0.30 |
|  | Haug 2009 | 3 SMS/wk with tailored feedback versus weekly SMS | Self-efficacy | 0.00 | -0.28 | 0.28 |
|  | Haug 2009 | 1 SMS/wk with tailored feedback versus weekly SMS | Any attempt to quit during follow up | 0.99 | 0.63 | 1.55 |
|  | McDaniel 2015 (10) | IVR - high intensity vs quitline | Self-reported 30 day abstinence at 12 months | 0.93 | 0.83 | 1.05 |
|  | McDaniel 2015 | IVR - low intensity versus quitline | Self-reported 7 day abstinence at 12 months | 1.02 | 0.91 | 1.14 |
|  | McDaniel 2015 | IVR - low intensity versus quitline | Self-reported 30 day abstinence at 12 months | 1.00 | 0.89 | 1.12 |
|  | McDaniel 2015 | IVR - high intensity vs quitline | Self-reported 7 day abstinence at 12 months | 0.98 | 0.87 | 1.09 |
|  | Rodgers 2005 (11) | 5 SMS/day versus 1 SMS/fortnight | 24 wk near-abstinence (self-reported) (<4 lapses of <3 cigarettes) | 1.64 | 1.12 | 2.42 |
|  | Rodgers 2005 | 5 SMS/day versus 1 SMS/fortnight | 24 wk complete abstinence | 1.50 | 0.92 | 2.44 |
|  | Rodgers 2005 | 5 SMS/day versus 1 SMS/fortnight | 1 wk point prevalence abstinence (Self-reported) - 6 wk | 1.07 | 0.91 | 1.26 |
|  | Shi 2013 (12) | SMS versus leaflet only | 30 day abstinence (self-reported) - 12 wk | 0.95 | 0.39 | 2.27 |
|  | Shi 2013 | SMS versus leaflet only | Reduction in smoking - 12 wk (no. participants) | 0.95 | 0.77 | 1.16 |
|  | Shi 2013 | SMS versus leaflet only | Cigarettes per day | -0.50 | -2.13 | 1.13 |
|  | Shi 2013 | SMS versus leaflet only | Improvement in stage of change | 0.95 | 0.72 | 1.24 |
|  | Shi 2013 | SMS versus leaflet only | Cognition on the health effects of smoking (Scale) | -0.40 | -1.67 | 0.87 |
|  | Shi 2013 | SMS versus leaflet only | Attitude towards disadvantages of smoking (Scale) | 1.50 | -5.13 | 8.13 |
|  | Shi 2013 | SMS versus leaflet only | Fagerstrom nicotine dependence score | -0.30 | -0.91 | 0.31 |
|  | Shi 2013 | SMS versus leaflet only | Cigarette dependence scale | -1.70 | -4.79 | 1.39 |
|  | Shi 2013 | SMS versus leaflet only | 1 wk abstinence (self-reported) - 12 wk | 0.95 | 0.47 | 1.93 |
|  | Skov-Ettrup 2014 (13) | Tailored SMS versus non-tailored SMS | 30 day abstinence (self-reported) – 1 yr | 1.39 | 0.95 | 2.03 |
|  | Vidrine 2006 (14) | Counseling sessions on mobile phone versus usual care | 24 hour abstinence (during 3 mo follow-up - self-reported) | 1.31 | 1.08 | 1.59 |
|  | Vidrine 2006 | Counseling sessions on mobile phone versus usual care | Self-efficacy | 0.17 | -0.80 | 1.14 |
|  | Vidrine 2006 | Counseling sessions on mobile phone versus usual care | Anxiety (score/points) | 1.74 | -4.35 | 7.82 |
|  | Vidrine 2006 | Counseling sessions on mobile phone versus usual care | Depression (score/points) | -1.44 | -6.73 | 3.85 |
|  | Vidrine 2006 | Counseling sessions on mobile phone versus usual care | Social support (score/points) | -0.84 | -12.10 | 10.42 |
|  | Vidrine 2006 | Counseling sessions on mobile phone versus usual care | Longest period of continuous abstinence (self-reported) (days) | 14.20 | 2.92 | 25.48 |
|  | Whittaker 2011 (15) | Video and SMS versus generic video | Continuous abstinence (self-reported) - 6 mo | 0.96 | 0.62 | 1.47 |
|  | Whittaker 2011 | Video and SMS versus generic video | 7 day point prevalence abstinence - 6 mo (self-reported) | 1.01 | 0.63 | 1.64 |
|  | Whittaker 2011 | Video and SMS versus generic video | Medication use - 6 mo follow up | 0.69 | 0.40 | 1.20 |
|  | Whittaker 2011 | Video and SMS versus generic video | Failure to quit (Number of participants) | 1.85 | 0.56 | 6.13 |
|  | Ybarra 2012 (16) | SMS versus no SMS | 30 day point prevalence abstinence (self-reported) - 3 mo | 1.97 | 0.62 | 6.28 |
|  | Ybarra 2012 | SMS versus no SMS | Cigarettes per day | -4.60 | -7.43 | -1.77 |
|  | Ybarra 2012 | SMS versus no SMS | 1 wk abstinence (self-reported) - 3 mo | 2.47 | 0.81 | 7.52 |
|  | Ybarra 2013 (17) | Tailored SMS versus non-tailored SMS | Continuous abstinence (self-reported) - 3 mo | 1.31 | 0.84 | 2.05 |
|  | Ybarra 2013 | Tailored SMS versus non-tailored SMS | Cigarettes per day | 0.80 | -1.16 | 2.76 |
|  | Ybarra 2013 | Tailored SMS versus non-tailored SMS | 1 wk abstinence (self-reported) - 4 wk | 1.44 | 0.81 | 2.55 |

**Table 2: Secondary outcomes diet, physical activity and diet, and physical activity**

| Clinical Area | Trial | Intervention | Outcome | RR/MD | LCI | UCI |
| --- | --- | --- | --- | --- | --- | --- |
| Diet and physical activity | | | | | | |
|  | Allen 2013 (18) | Smartphone app and intensive counseling versus counseling only | Total daily energy intake (Kcal) | -52.6 | -483.56 | 378.36 |
|  | Allen 2013 | Smartphone app and intensive counseling versus counseling only | Dietary fat consumption (% daily kcals from fat) | -4.22 | -10.28 | 1.84 |
|  | Allen 2013 | Smartphone app and intensive counseling versus counseling only | Fruit and vegetable consumption (servings per day) | -0.3 | -2.77 | 2.17 |
|  | Allen 2013 | Smartphone app and intensive counseling versus counseling only | Sodium intake (Mg per day) | -271 | -1097.9 | 555.09 |
|  | Allen 2013 | Smartphone app and intensive counseling versus counseling only | Physical activity (hours per week) | -0.6 | -5.73 | 4.53 |
|  | Chow 2015 (19) | SMS versus usual care | physical activity MET min/wk | 345 | 195 | 495 |
|  | Chow 2015 | SMS versus usual care | Inactive <600 MET min/wk | 0.55 | 0.47 | 0.64 |
|  | Fassnacht 2015 (20) | SMS versus no SMS | Physical activity (hours per day) at 12 weeks | 0.2 | -0.53 | 0.93 |
|  | Fassnacht 2015 | SMS versus no SMS | Fruit and veg (portions per day) | 0.2 | -0.53 | 0.93 |
|  | Golshahi 2015 (7) | SMS versus pamphlets | Physical activity (minutes/day) change at 8 months | 1.47 | -1.96 | 4.9 |
|  | Golshahi 2015 | SMS versus pamphlets | Vegetable intake (time/day) change at 8 months | -0.02 | -0.13 | 0.09 |
|  | Golshahi 2015 | SMS versus pamphlets | High salt intake at 8 months | 1.2 | 0.58 | 2.49 |
|  | Haapala 2009 (21) | SMS versus no SMS | Self efficacy in dieting score - SMD | -0.2 | -0.87 | 0.47 |
|  | Haapala 2009 | SMS versus no SMS | Energy-dense food consumption score - SMD | 0 | -0.28 | 0.28 |
|  | Hebden 2014 (22) | SMS, email messages, booklet, dietition session versus booklet and dietition session only | Moderate and vigorous physical activity (min per day) change at 3 months | 1.81 | -11.18 | 14.8 |
|  | Hebden 2014 | SMS, email messages, booklet, dietition session versus booklet and dietition session only | Light physical activity (min per day) change at 3 months | 20.27 | -6.71 | 47.25 |
|  | Hebden 2014 | SMS, email messages, booklet, dietition session versus booklet and dietition session only | Sedentary (min per day) change at 3 months | -22.26 | -55.6 | 11.07 |
|  | Hebden 2014 | SMS, email messages, booklet, dietition session versus booklet and dietition session only | International physical activity questionnaire (min per week) change at 3 months | 105.12 | -11.8 | 222.04 |
|  | Hebden 2014 | SMS, email messages, booklet, dietition session versus booklet and dietition session only | International physical activity questionnaire (metabolic equivalent of task - min per week) change at 3 months | 337.31 | -163.27 | 837.89 |
|  | Hebden 2014 | SMS, email messages, booklet, dietition session versus booklet and dietition session only | Sitting (min per day) change at 3 months | -9.81 | -122.51 | 102.89 |
|  | Hebden 2014 | SMS, email messages, booklet, dietition session versus booklet and dietition session only | Veg intake (servings per day) change at 3 months | -0.12 | -0.7 | 0.45 |
|  | Hebden 2014 | SMS, email messages, booklet, dietition session versus booklet and dietition session only | fruit intake (servings per day) change at 3 months | -0.01 | -0.51 | 0.49 |
|  | Hebden 2014 | SMS, email messages, booklet, dietition session versus booklet and dietition session only | sugar sweetened beverages (mL week) change at 3 months | -18.69 | -470.66 | 433.27 |
|  | Hebden 2014 | SMS, email messages, booklet, dietition session versus booklet and dietition session only | Energy dense takeaway meals (<1 time week) at 3 months | 0.99 | 0.23 | 4.17 |
|  | Kim 2015 (23) | Tailored SMS and educational sessions versus education sessions only | Physical activity level (MET-minutes/week) at 6 months change | 580.4 | -179.18 | 1339.98 |
|  | Kim 2015 | Tailored SMS and educational sessions versus education sessions only | Obesity QoL change at 6 months | -0.32 | -4.33 | 3.69 |
|  | Laing 2014 (24) | app versus encouragement to choose any weight loss activity | Self report healthy diet past 7 days - change (number of days followed)- month 6 | 0.29 | -0.51 | 1.1 |
|  | Laing 2014 | app versus encouragement to choose any weight loss activity | Self report physical activity past 7 days - change (number of days followed)- month 6 | 0.2 | -0.49 | 0.9 |
|  | Laing 2014 | app versus encouragement to choose any weight loss activity | Self report exercise past 7 days - change (number of days followed)- month 6 | 0.4 | -0.35 | 1.2 |
|  | Laing 2014 | app versus encouragement to choose any weight loss activity | Used calorie goal in past 7 days (number of days followed) - change - 6 months | 2 | 1.1 | 2.9 |
|  | Laing 2014 | app versus encouragement to choose any weight loss activity | Self-efficacy in achieving weight loss goal (scale of 0-10) - change - 6 months | -0.44 | -1.1 | 0.21 |
|  | Laing 2014 | app versus encouragement to choose any weight loss activity | Self-efficacy in making healthy food/exercise choices (scale of 0-10) - change - 6 months | -0.03 | -0.74 | 0.69 |
|  | Patrick 2013 (25) | SMS versus no SMS | Sedentary behaviour (hours/day) | 0.8 | -2.95 | 4.55 |
|  | Patrick 2013 | SMS versus no SMS | Dietary fat consumption (% daily Kcals from fat) | -1.5 | -1.53 | -1.47 |
|  | Patrick 2013 | SMS versus no SMS | Fruit and vegetable consumption (servings per 1000 kcals) | -0.3 | -0.33 | -0.27 |
|  | Patrick 2013 | SMS versus no SMS | Quality of life (general) | 0.9 | -6.69 | 8.49 |
|  | Patrick 2013 | SMS versus no SMS | Quality of life (physical) | -1 | -8.38 | 6.38 |
|  | Patrick 2013 | SMS versus no SMS | Self-esteem | 3 | -0.69 | 6.69 |
|  | Patrick 2013 | SMS versus no SMS | Depression | 0 | -2.87 | 2.87 |
|  | Patrick 2013 | SMS versus no SMS | physical activity behaviour strategy use | 0.5 | -0.07 | 1.07 |
|  | Patrick 2013 | SMS versus no SMS | fruit & vegetable behaviour strategy use | 0 | -0.59 | 0.59 |
|  | Patrick 2013 | SMS versus no SMS | dietary fat behaviour strategy use | 0.3 | -0.31 | 0.91 |
|  | Patrick 2013 | SMS versus no SMS | sedentary behaviour strategy use | 0 | -0.66 | 0.66 |
|  | Patrick 2013 | SMS versus no SMS | Physical activity (minutes per week moderate/vigorous activity) | -4.4 | -11.65 | 2.85 |
|  | Ramachandran 2013 (26) | SMS versus standard advice | Total daily energy intake (Kcal) | -43.8 | -91.63 | 4.03 |
|  | Ramachandran 2013 | SMS versus standard advice | Physical activity score | 1 | -3.4 | 5.4 |
|  | Shahid 2015 (27) | Voice call delivered feedback versus no feedback | following diet plan at 4 months | 2.94 | 2.11 | 4.11 |
|  | Shahid 2015 | Voice call delivered feedback versus no feedback | physically active at 4 months | 2.97 | 2.14 | 4.13 |
|  | Shapiro 2008 (28) | SMS versus E-newsletters | TV/computer screen usage (Minutes per day) | -31.2 | -89.01 | 26.61 |
|  | Shapiro 2008 | SMS versus E-newsletters | Physical activity (pedometer steps per day) | -2740.4 | -5217.23 | -263.57 |
|  | Shapiro 2008 | SMS versus E-newsletters | Sugar sweetened beverage servings per day | 0.3 | -0.23 | 0.83 |
|  | Shapiro 2008 | SMS versus E-newsletters | Sugar sweetened beverage servings per day | -0.3 | -0.96 | 0.36 |
|  | Shapiro 2008 | SMS versus E-newsletters | Sugar sweetened beverage servings per day | 0.3 | -0.34 | 0.94 |
|  | Shapiro 2008 | SMS versus E-newsletters | Exercise (Minutes per day) | 23.2 | -96.34 | 142.74 |
|  | Shaw 2013 (29) | Promotion-framed SMS versus attention-control SMS | Body weight (pounds) | 5.6 | -19.72 | 30.92 |
|  | Shaw 2013 | Prevention-framed SMS versus attention-control SMS | Sustained weight loss (number of participants) | 1.11 | 0.99 | 1.25 |
|  | Shaw 2013 | Promotion-framed SMS versus attention-control SMS | Sustained weight loss (number of participants) | 1.06 | 0.93 | 1.2 |
|  | Shaw 2013 | Prevention-framed SMS versus attention-control SMS | Body weight (pounds) | 5.6 | -19.54 | 30.74 |
|  | Svetkey 2015 (30) | App versus no app | Change in Healthy Eating Index (HEI) (kcal/wk) at 24 months | -1.93 | -3.75 | -0.11 |
|  | Svetkey 2015 | App versus no app | Change in Leisure Time Physical Activity (kcal/wk) at 24 months | 146.59 | -184.84 | 478.03 |
|  | Turner-McGrievey 2011 (31) | SMS and podcast versus SMS only | Total daily energy intake - change (Kcal) | -46.3 | -268.73 | 176.13 |
|  | Turner-McGrievey 2011 | SMS and podcast versus SMS only | Dietary fat consumption - change (grams) | -0.5 | -12.22 | 11.22 |
|  | Turner-McGrievey 2011 | SMS and podcast versus SMS only | Weight-loss self-efficacy score - change | -2.5 | -12.76 | 7.76 |
|  | Turner-McGrievey 2011 | SMS and podcast versus SMS only | Weight-loss knowledge score - change | -0.44 | -1.14 | 0.26 |
|  | Turner-McGrievey 2011 | SMS and podcast versus SMS only | Eating behaviours score - change | 2.6 | -1.94 | 7.14 |
|  | Turner-McGrievey 2011 | SMS and podcast versus SMS only | Energy expenditure - change (kcal) | -9.9 | -83.44 | 63.64 |
|  | Van Drongelen 2014 (32) | App and website versus website only | Number of snacks per duty - 3 months | -0.75 | -1.2 | 0.3 |
|  | Van Drongelen 2014 | App and website versus website only | Moderate physical activity (days per week) - 3 months | 0.07 | -0.17 | 0.31 |
|  | Van Drongelen 2014 | App and website versus website only | Strenuous physical activity (days per week) - 3 months | 0.17 | 0.02 | 0.32 |
|  | Van Drongelen 2014 | App and website versus website only | Health perception- health appreciation % - 3 months | 1.87 | -0.65 | 4.59 |
|  | Varnfield 2014 (33) | Mixed mobile technology versus usual care | Diet - fat (6 weeks) - grams change at 6 wks | 0.09 | -0.12 | 0.3 |
|  | Varnfield 2014 | Mixed mobile technology versus usual care | Diet - fibre (6 weeks) - grams change at 6 wks | 0.04 | -0.17 | 0.24 |
|  | Varnfield 2014 | Mixed mobile technology versus usual care | Diet - sodium (6 weeks) - milligrams change at 6 wks | -0.11 | -0.38 | 0.16 |
|  | Varnfield 2014 | Mixed mobile technology versus usual care | Diet - alcohol (6 weeks) - change at 6 wks | 0.09 | -0.2 | 0.37 |
|  | Varnfield 2014 | Mixed mobile technology versus usual care | Quality of life EQ5D-Index | -0.08 | -0.14 | -0.02 |
|  | Varnfield 2014 | Mixed mobile technology versus usual care | Mental Health: Kessler 10 score | 1.85 | -0.11 | 3.81 |
|  | Varnfield 2014 | Mixed mobile technology versus usual care | DASS-depression scale | 0.9 | -0.77 | 2.57 |
|  | Varnfield 2014 | Mixed mobile technology versus usual care | DASS-anxiety scale | 1.63 | -0.21 | 3.46 |
|  | Varnfield 2014 | Mixed mobile technology versus usual care | DASS-stress scale | -1 | -3.81 | 1.81 |
|  | Varnfield 2014 | Mixed mobile technology versus usual care | Functional capacity - 6 minute walk test | -10.19 | -35 | 14.63 |
| Physical activity | | | | | | |
|  | Direito 2015 (34) | Zombies run app versus no app | PAQ - A (Physical Activity Questionnaire for Adolescents) -follow-up 8 wks | 0.14 | -0.26 | 0.54 |
|  | Direito 2015 | Zombies run app versus no app | PACES (Physical Activity Enjoyment Scale) -follow-up 8 wks | -0.1 | -0.33 | 0.13 |
|  | Direito 2015 | Zombies run app versus no app | PNSES (Psyhological Need Satisfaction in Exercise Scale) -follow-up 8 wks | -0.08 | -0.46 | 0.31 |
|  | Direito 2015 | Zombies run app versus no app | PNSES - competence -follow-up 8 wks | -0.08 | -0.67 | 0.51 |
|  | Direito 2015 | Zombies run app versus no app | PNSES - autonomy -follow-up 8 wks | 0.12 | -0.44 | 0.69 |
|  | Direito 2015 | Zombies run app versus no app | PNSES - relatedness -follow-up 8 wks | -0.26 | -0.88 | 0.35 |
|  | Direito 2015 | Zombies run app versus no app | PASES (Physical Activity Self-Efficacy Scale) -follow-up 8 wks | -0.02 | -0.24 | 0.19 |
|  | Direito 2015 | Get running non immersive app versus no app | PAQ - A (Physical Activity Questionnaire for Adolescents) -follow-up 8 wks | 0.23 | -0.18 | 0.64 |
|  | Direito 2015 | Get running non immersive app versus no app | PACES (Physical Activity Enjoyment Scale) -follow-up 8 wks | -0.17 | -0.4 | 0.06 |
|  | Direito 2015 | Get running non immersive app versus no app | PNSES (Psychological Need Satisfaction in Exercise Scale) -follow-up 8 wks | 0.01 | -0.38 | 0.4 |
|  | Direito 2015 | Get running non immersive app versus no app | PNSES - competence -follow-up 8 wks | 0.03 | -0.57 | 0.63 |
|  | Direito 2015 | Get running non immersive app versus no app | PNSES - autonomy -follow-up 8 wks | 0.36 | -0.22 | 0.94 |
|  | Direito 2015 | Get running non immersive app versus no app | PNSES - relatedness -follow-up 8 wks | -0.34 | -0.97 | 0.29 |
|  | Direito 2015 | Get running non immersive app versus no app | PASES (Physical Activity Self-Efficacy Scale) -follow-up 8 wks | 0.04 | -0.18 | 0.26 |
|  | Filion 2015 (35) | Sleep/activity SMS versus smoking cessation SMS | Relative difference in physical activity between groups at 3-month follow-up | -1.93 | -5.68 | 1.83 |
|  | Glynn 2014 (36) | Smartphone app versus no app | Quality of life (EQ-VAS) | -2.2 | -8.96 | 4.56 |
|  | Glynn 2014 | Smartphone app versus no app | Quality of life (EQ5D) | 0 | -0.03 | 0.03 |
|  | Glynn 2014 | Smartphone app versus no app | Hospital Anxiety and Depression Scale | -0.3 | -1.22 | 0.62 |
|  | Kim 2013 (37) | Motivational SMS versus no SMS | Activity levels (MET) | 8.86 | 5.44 | 12.28 |
|  | Liu 2008 (38) | Endurance exercises accompanied by music on mobile phone versus booklet and DVD | Quality of life (physical) score - SMD | 17 | 15.94 | 18.07 |
|  | Maddison 2014 (39) | SMS plus usual care versus usual care | Total physical activity (minutes per wk)* | 233.9 | -146.5 | 614.2 |
|  | Maddison 2014 | SMS plus usual care versus usual care | Leisure time physical activity (minutes per wk)* | 110.2 | -0.8 | 221.3 |
|  | Maddison 2014 | SMS plus usual care versus usual care | Walking (minutes per wk)* | 151.4 | 27.6 | 275.2 |
|  | Maddison 2014 | SMS plus usual care versus usual care | Quality of life score (physical functioning)* | 1 | -0.6 | 2.7 |
|  | Maddison 2014 | SMS plus usual care versus usual care | Quality of life score (role physical)* | 1.8 | -0.3 | 3.9 |
|  | Maddison 2014 | SMS plus usual care versus usual care | Quality of life score (bodily pain)* | 0.5 | -2.1 | 3.1 |
|  | Maddison 2014 | SMS plus usual care versus usual care | Quality of life score (general health)* | 2.1 | 0.1 | 4.1 |
|  | Maddison 2014 | SMS plus usual care versus usual care | Quality of life score (vitality)* | -0.3 | -2.2 | 1.7 |
|  | Maddison 2014 | SMS plus usual care versus usual care | Quality of life score (social functioning)* | 0.9 | -1.3 | 3.1 |
|  | Maddison 2014 | SMS plus usual care versus usual care | Quality of life score (role emotional)* | -0.2 | -2.5 | 1.9 |
|  | Maddison 2014 | SMS plus usual care versus usual care | Quality of life score (mental health)* | 0.5 | -1.5 | 2.6 |
|  | Newton 2009 (40) | Motivational SMS versus standard care | Exercise - change (Minutes per wk) | 9.9 | -181.09 | 200.89 |
|  | Newton 2009 | Motivational SMS versus standard care | Quality of life (general) score - SMD | -0.92 | -1.89 | 0.05 |
|  | Prestwich 2009 (41) | Implementation messages versus protection-motivation theory-based statement only | Exercise behaviour | 0.11 | -0.4 | 0.62 |
|  | Prestwich 2009 | Implementation messages versus no treatment | Exercise behaviour | 0.07 | -0.45 | 0.59 |
|  | Prestwich 2009 | Implementation messages plus SMS versus no treatment | Exercise behaviour | 0.54 | -0.05 | 1.13 |
|  | Prestwich 2009 | Implementation messages plus SMS versus protection-motivation theory-based statement only | Exercise behaviour | 0.58 | 0.01 | 1.15 |
|  | Prestwich 2009 | SMS reminders only versus no treatment | Exercise behaviour | 0.16 | -0.34 | 0.66 |
|  | Prestwich 2009 | SMS reminders only versus protection-motivation theory-based statement only | Exercise behaviour | 0.2 | -0.28 | 0.68 |
|  | Prestwich 2010 (42) | SMS goal reminders versus no SMS reminders | Exercise - days per week ≥30 mins | 0.53 | -0.27 | 1.33 |
|  | Prestwich 2010 | SMS plan reminders versus no SMS reminders | Exercise - days per week ≥30 mins | 0.85 | -47.81 | 49.51 |
|  | Prestwich 2010 | SMS goal reminders versus no SMS reminders | Physical activity - walking - days per week ≥30 mins | 0.81 | -0.01 | 1.63 |
|  | Prestwich 2010 | SMS plan reminders versus no SMS reminders | Physical activity - walking - days per week ≥30 mins | 0.81 | -0.03 | 1.6 |
|  | Van der Weegen 2015 (43) | Activity monitor, app, self-management support program versus self-management support program only | General self-efficacy scale at 9 months | 0.01 | -0.13 | 0.14 |
|  | Van der Weegen 2015 | Activity monitor, app, self-management support program versus self-management support program only | Exercise self-efficacy scale at 9 months | -3.63 | -11.69 | 4.43 |
|  | Van der Weegen 2015 | Activity monitor, app, self-management support program versus self-management support program only | QoL RAND physical component at 9 months | -2.65 | -4.99 | -0.32 |
| Diet | | | | | | |
|  | Soureti 2011 (44) | Planning tool and text reminders versus planning tool only | Saturated fat intake - change (score) | 0.30 | -0.25 | 0.85 |
|  | Soureti 2011 | Planning tool and text reminders versus planning tool only | Saturated fat intake - change (% daily calories from fat) | 0.30 | 0.02 | 0.58 |

**Table 3:** **Secondary outcomes - alcohol**

| Clinical Area | Trial | Intervention | Outcome | RR/MD | LCI | UCI |
| --- | --- | --- | --- | --- | --- | --- |
| Alcohol use |  |  |  |  |  |  |
|  | Agyapong 2013 (45) | Supportive SMS versus fortnightly thank you SMS | Longest period of abstinence since cessation of supportive SMS to endpoint | 9.41 | -2.80 | 21.62 |
|  | Agyapong 2013 | Supportive SMS versus fortnightly thank you SMS | Longest period of abstinence (days to first drink following inpatient discharge) | 57.50 | 30.68 | 84.32 |
|  | Agyapong 2013 | Supportive SMS versus fortnightly thank you SMS | Continuous abstinence (no. of participants) | 0.81 | 0.42 | 1.55 |
|  | Agyapong 2013 | Supportive SMS versus fortnightly thank you SMS | Alcohol consumed per day of drinking - 6 mo (units) | -0.99 | -5.14 | 3.16 |
|  | Agyapong 2013 | Supportive SMS versus fortnightly thank you SMS | Depression (Becks depression inventory) | -1.80 | -7.62 | 4.02 |
|  | Agyapong 2013 | Supportive SMS versus fortnightly thank you SMS | Self-efficacy of alcohol abstinence (scale) | 4.50 | -2.75 | 11.75 |
|  | Agyapong 2013 | Supportive SMS versus fortnightly thank you SMS | Ability to function (scale) | 9.71 | -0.42 | 19.84 |
|  | Agyapong 2013 | Supportive SMS versus fortnightly thank you SMS | Obsessive compulsive drinking (scale) | -3.00 | -6.70 | 0.70 |
|  | Andersson 2015 (46) | IVR personalised versus no intervention | Daily Drinking Questionnaire (DDQ) quantity scores (change) | -0.30 | -0.9 | 0.3 |
|  | Andersson 2015 | IVR personalised versus no intervention | DDQ frequency scores (change) | 0 | -0.1 | 0.2 |
|  | Andersson 2015 | IVR personalised versus no intervention | DDQ mean BAC scores (change) | -0.002 | -0.002 | 0.003 |
|  | Andersson 2015 | IVR personalised versus no intervention | Alcohol Use Disorders Identification Test (AUDIT) scores (change) | -0.60 | -1.00 | -0.30 |
|  | Gajecki 2014 (47) | Promillekoll app versus no app | quantity (stand glasses/week) 7 weeks | 1.13 | 0.29 | 1.97 |
|  | Gajecki 2014 | Promillekoll app versus no app | Frequency (drinking occasions/week) 7 weeks | 0.21 | 0.06 | 0.36 |
|  | Gajecki 2014 | Promillekoll app versus no app | Binge occasions no/week 7 weeks | 0.12 | 0.02 | 0.23 |
|  | Gajecki 2014 | Promillekoll app versus no app | eBAC/week 7 weeks | 0.00 | 0.00 | 0.00 |
|  | Gajecki 2014 | Promillekoll app versus no app | Peak eBAC/month 7 weeks | 0.00 | -0.01 | 0.01 |
|  | Gajecki 2014 | PartyPlanner app versus no app | quantity (stand glasses/week) 7 weeks | -0.30 | -1.18 | 0.58 |
|  | Gajecki 2014 | PartyPlanner app versus no app | Frequency (drinking occasions/week) 7 weeks | 0.02 | -0.15 | 0.19 |
|  | Gajecki 2014 | PartyPlanner app versus no app | Binge occasions no/week 7 weeks | -0.11 | -0.22 | 0.01 |
|  | Gajecki 2014 | PartyPlanner app versus no app | eBAC/week 7 weeks | -0.001 | -0.003 | 0.001 |
|  | Gajecki 2014 | PartyPlanner app versus no app | Peak eBAC/month 7 weeks | 0.00 | -0.01 | 0.02 |
|  | Gustafson 2014 (48) | Smartphone App versus usual care | Continuous abstinence (no. of participants) | 1.14 | 0.97 | 1.35 |
|  | Gustafson 2014 | Smartphone App versus usual care | No. of risky drinking days | -1.47 | -2.84 | -0.10 |
|  | Haug 2015 (49) | SMS versus no SMS | mean number of days without drinking alcohol over the 30 days prior | 2.2 | -3.53 | 7.93 |
|  | Haug 2015 | SMS versus no SMS | Alcohol use (at risk AUDIT-c >=4) at 6 months | 0.69 | 0.30 | 1.57 |
|  | Mason, 2014 (50) | SMS versus no SMS | number of drinks past week | 4.70 | -2.88 | 12.28 |
|  | Mason, 2014 | SMS versus no SMS | number of drinks last occasion | 3.2 | -0.62 | 7.02 |
|  | Mason, 2014 | SMS versus no SMS | Maximum number of drinks past month | 3.3 | -2.10 | 8.70 |
|  | Mason, 2014 | SMS versus no SMS | Average number of drinks per occasion last month | 0.60 | -1.57 | 2.77 |
|  | Suffoletto 2012 (51) | SMS feedback plus goal setting versus usual care | No. days of heavy drinking | 3.50 | -0.25 | 7.25 |
|  | Suffoletto 2012 | SMS drinking assessment with no feedback versus usual care | No. days of heavy drinking | -0.80 | -3.54 | 1.94 |
|  | Suffoletto 2012 | SMS feedback plus goal setting versus usual care | No. drinks per drinking day | 1.40 | -0.14 | 2.94 |
|  | Suffoletto 2012 | SMS drinking assessment with no feedback versus usual care | No. drinks per drinking day | -0.70 | -2.03 | 0.63 |
|  | Suffoletto 2012 | SMS feedback plus goal setting versus usual care | No. subjects with no heavy drinking days | 0.93 | 0.35 | 2.48 |
|  | Suffoletto 2012 | SMS drinking assessment with no feedback versus usual care | No. subjects with no heavy drinking days | 0.20 | 0.03 | 1.49 |
|  | Suffoletto 2014 (52) | SMS drinking assessment with real time feedback (SA+F) | Number of binge drinking days in last 30 days (change at 12 weeks) | -0.92 | -1.66 | -0.18 |
|  | Suffoletto 2014 | SMS drinking assessment with real time feedback (SA+F) versus no sms | Number of drinks per drinking day (change at 12 weeks) | -0.70 | -1.11 | -0.29 |
|  | Suffoletto 2014 | SMS drinking assessment with no feedback (SA) versus no sms | Number of binge drinking days in last 30 days (change at 12 weeks) | 0.49 | -0.42 | 1.40 |
|  | Suffoletto 2014 | SMS drinking assessment with no feedback (SA) versus no sms | Number of drinks per drinking day (change at 12 weeks) | -0.29 | -0.79 | 0.21 |

1. Abroms LC, Boal AL, Simmens SJ, Mendel JA, Windsor RA. A randomized trial of Text2Quit: a text messaging program for smoking cessation. American journal of preventive medicine. 2014;47(3):242-50.

2. Borland R, Balmford J, Benda P. Population-level effects of automated smoking cessation help programs: a randomized controlled trial. Addiction. 2013;108(3):618-28.

3. Buller DB, Borland R, Bettinghaus EP, Shane JH, Zimmerman DE. Randomized trial of a smartphone mobile application compared to text messaging to support smoking cessation. Telemedicine and e health. 2014;20(3):206-14.

4. Chan SS, Wong DC, Cheung YTD, Leung DY, Lau L, Lai V, et al. A block randomized controlled trial of a brief smoking cessation counselling and advice through short message service on participants who joined the Quit to Win Contest in Hong Kong. Health education research. 2015:cyv023.

5. Free C, Whittaker R, Knight R, Abramsky T, Rodgers A, Roberts IG. Txt2stop: a pilot randomised controlled trial of mobile phone-based smoking cessation support. Tobacco Control. 2009;18(2):88-91.

6. Free C, Knight R, Robertson S, Whittaker R, Edwards P, Zhou W, et al. Smoking cessation support delivered via mobile phone text messaging (txt2stop): a single-blind, randomised trial. Lancet. 2011;378(9785):49-55.

7. Golshahi J, Ahmadzadeh H, Sadeghi M, Mohammadifard N, Pourmoghaddas A. Effect of self-care education on lifestyle modification, medication adherence and blood pressure in hypertensive adults: Randomized controlled clinical trial. Advanced biomedical research. 2015;4.

8. Gritz ER, Danysh HE, Fletcher FE, Tami-Maury I, Fingeret MC, King RM, et al. Long-term outcomes of a cell phone-delivered intervention for smokers living with HIV/AIDS. Clinical Infectious Diseases. 2013;57(4):608-15.

9. Haug S, Meyer C, Schorr G, Bauer S, John U. Continuous individual support of smoking cessation using text messaging: a pilot experimental study. Nicotine & Tobacco Research. 2009;11(8):915-23.

10. McDaniel AM, Vickerman KA, Stump TE, Monahan PO, Fellows JL, Weaver MT, et al. A randomised controlled trial to prevent smoking relapse among recently quit smokers enrolled in employer and health plan sponsored quitlines. BMJ open. 2015;5(6):e007260.

11. Rodgers A, Corbett T, Bramley D, Riddell T, Wills M, Lin RB, et al. Do u smoke after txt? Results of a randomised trial of smoking cessation using mobile phone text messaging. Tobacco Control. 2005;14(4):255-61.

12. Shi HJ, Jiang XX, Yu CY, Zhang Y. Use of mobile phone text messaging to deliver an individualized smoking behaviour intervention in Chinese adolescents. Journal of Telemedicine & Telecare. 2013;19(5):282-7.

13. Skov-Ettrup LS, Ringgaard LW, Dalum P, Flensborg-Madsen T, Thygesen LC, Tolstrup JS. Comparing tailored and untailored text messages for smoking cessation: a randomized controlled trial among adolescent and young adult smokers. Health Education Research. 2014;29(2):195-205.

14. Vidrine DJ, Arduino RC, Gritz ER. Impact of a cell phone intervention on mediating mechanisms of smoking cessation in individuals living with HIV/AIDS. Nicotine & Tobacco Research. 2006;8 Suppl 1:S103-8.

15. Whittaker R, Dorey E, Bramley D, Bullen C, Denny S, Elley CR, et al. A theory-based video messaging mobile phone intervention for smoking cessation: randomized controlled trial. Journal of Medical Internet Research. 2011;13(1):e10.

16. Ybarra M, Bagci Bosi AT, Korchmaros J, Emri S. A text messaging-based smoking cessation program for adult smokers: randomized controlled trial. Journal of Medical Internet Research. 2012;14(6):e172.

17. Ybarra ML, Holtrop JS, Prescott TL, Rahbar MH, Strong D. Pilot RCT results of stop my smoking USA: a text messaging-based smoking cessation program for young adults. Nicotine & Tobacco Research. 2013;15(8):1388-99.

18. Allen JK, Stephens J, Dennison Himmelfarb CR, Stewart KJ, Hauck S. Randomized controlled pilot study testing use of smartphone technology for obesity treatment. Journal of Obesity. 2013;2013(151597).

19. Chow CK, Redfern J, Hillis GS, Thakkar J, Santo K, Hackett ML, et al. Effect of lifestyle-focused text messaging on risk factor modification in patients with coronary heart disease: a randomized clinical trial. Jama. 2015;314(12):1255-63.

20. Fassnacht DB, Ali K, Silva C, Goncalves S, Machado PP. Use of text messaging services to promote health behaviors in children. Journal of nutrition education and behavior. 2015;47(1):75-80.

21. Haapala I, Barengo NC, Biggs S, Surakka L, Manninen P. Weight loss by mobile phone: a 1-year effectiveness study. Public Health Nutrition. 2009;12(12):2382-91.

22. Hebden L, Cook A, Ploeg H, King L, Bauman A, Allman‐Farinelli M. A mobile health intervention for weight management among young adults: a pilot randomised controlled trial. Journal of Human Nutrition and Dietetics. 2014;27(4):322-32.

23. Kim J-Y, Oh S, Steinhubl S, Kim S, Bae WK, Han JS, et al. Effectiveness of 6 months of tailored text message reminders for obese male participants in a worksite weight loss program: randomized controlled trial. JMIR mHealth and uHealth. 2015;3(1).

24. Laing BY, Mangione CM, Tseng C-H, Leng M, Vaisberg E, Mahida M, et al. Effectiveness of a smartphone application for weight loss compared with usual care in overweight primary care patients: a randomized, controlled trial. Annals of internal medicine. 2014;161(10_Supplement):S5-S12.

25. Patrick K, Norman GJ, Davila EP, Calfas KJ, Raab F, Gottschalk M, et al. Outcomes of a 12-month technology-based intervention to promote weight loss in adolescents at risk for type 2 diabetes. Journal of Diabetes Science & Technology. 2013;7(3):759-70.

26. Ramachandran A, Snehalatha C, Ram J, Selvam S, Simon M, Nanditha A, et al. Effectiveness of mobile phone messaging in prevention of type 2 diabetes by lifestyle modification in men in India: A prospective, parallel-group, randomised controlled trial. The Lancet Diabetes and Endocrinology. 2013;1(3):191-8.

27. Shahid M, Mahar SA, Shaikh S, Shaikh Z. Mobile Phone Intervention to Improve Diabetes Care in Rural Areas of Pakistan: A Randomized Controlled Trial. Journal of the College of Physicians and Surgeons--Pakistan: JCPSP. 2015;25(3):166-71.

28. Shapiro JR, Bauer S, Hamer RM, Kordy H, Ward D, Bulik CM. Use of text messaging for monitoring sugar-sweetened beverages, physical activity, and screen time in children: a pilot study. Journal of Nutrition Education & Behavior. 2008;40(6):385-91.

29. Shaw RJ, Bosworth HB, Silva SS, Lipkus IM, Davis LL, Sha RS, et al. Mobile health messages help sustain recent weight loss. American Journal of Medicine. 2013;126(11):1002-9.

30. Svetkey LP, Batch BC, Lin P-H, Intille SS, Corsino L, Tyson CC, et al. Cell phone intervention for you (CITY): A randomized, controlled trial of behavioral weight loss intervention for young adults using mobile technology. Obesity. 2015;23(11):2133-41.

31. Turner-McGrievy G, Tate D. Tweets, Apps, and Pods: Results of the 6-month Mobile Pounds Off Digitally (Mobile POD) randomized weight-loss intervention among adults. Journal of Medical Internet Research. 2011;13(4):e120.

32. van Drongelen A, van der Beek AJ. Evaluation of an mHealth intervention aiming to improve health-related behavior and sleep and reduce fatigue among airline pilots. Scandinavian journal of work, environment & health. 2014;40(6):557.

33. Varnfield M, Karunanithi M, Lee C-K, Honeyman E, Arnold D, Ding H, et al. Smartphone-based home care model improved use of cardiac rehabilitation in postmyocardial infarction patients: results from a randomised controlled trial. Heart. 2014:heartjnl-2014-305783.

34. Direito A, Jiang Y, Whittaker R, Maddison R. Apps for IMproving FITness and increasing physical activity among young people: The AIMFIT pragmatic randomized controlled trial. Journal of medical Internet research. 2015;17(8).

35. Filion AJ, Darlington G, Chaput J-P, Ybarra M, Haines J. Examining the influence of a text message-based sleep and physical activity intervention among young adult smokers in the United States. BMC public health. 2015;15(1):1.

36. Glynn LG, Hayes PS, Casey M, Glynn F, Alvarez-Iglesias A, Newell J, et al. Effectiveness of a smartphone application to promote physical activity in primary care: The SMART MOVE randomised controlled trial. British Journal of General Practice. 2014;64(624):e384-e91.

37. Kim BH, Glanz K. Text messaging to motivate walking in older African Americans: a randomized controlled trial. American Journal of Preventive Medicine. 2013;44(1):71-5.

38. Liu WT, Wang CH, Lin HC, Lin SM, Lee KY, Lo YL, et al. Efficacy of a cell phone-based exercise programme for COPD. European Respiratory Journal. 2008;32(3):651-9.

39. Maddison R, Pfaeffli L, Whittaker R, Stewart R, Kerr A, Jiang Y, et al. A mobile phone intervention increases physical activity in people with cardiovascular disease: Results from the HEART randomized controlled trial. Eur J Prev Cardiol. 2014.

40. Newton KH, Wiltshire EJ, Elley CR. Pedometers and text messaging to increase physical activity: randomized controlled trial of adolescents with type 1 diabetes. Diabetes Care. 2009;32(5):813-5.

41. Prestwich A, Perugini M, Hurling R. Can the effects of implementation intentions on exercise be enhanced using text messages? Psychology & Health. 2009;24(6):677-87.

42. Prestwich A, Perugini M, Hurling R. Can implementation intentions and text messages promote brisk walking? A randomized trial. Health Psychology. 2010;29(1):40-9.

43. van der Weegen S, Verwey R, Spreeuwenberg M, Tange H, van der Weijden T, de Witte L. It's LiFe! Mobile and Web-Based Monitoring and Feedback Tool Embedded in Primary Care Increases Physical Activity: A Cluster Randomized Controlled Trial. Journal of medical Internet research. 2015;17(7).

44. Soureti A, Murray P, Cobain M, Chinapaw M, van Mechelen W, Hurling R. Exploratory study of web-based planning and mobile text reminders in an overweight population. Journal of Medical Internet Research. 2011;13(4):e118.

45. Agyapong VIO, McLoughlin DM, Farren CK. Six-months outcomes of a randomised trial of supportive text messaging for depression and comorbid alcohol use disorder. Journal of Affective Disorders. 2013;151(1):100-4.

46. Andersson C. Comparison of WEB and Interactive Voice Response (IVR) methods for delivering brief alcohol interventions to hazardous-drinking university students: a randomized controlled trial. European addiction research. 2015;21(5):240-52.

47. Gajecki M, Berman AH, Sinadinovic K, Rosendahl I, Andersson C. Mobile phone brief intervention applications for risky alcohol use among university students: a randomized controlled study. Addiction science & clinical practice. 2014;9(1):1.

48. Gustafson DH, McTavish FM, Chih MY, Atwood AK, Johnson RA, Boyle MG, et al. A smartphone application to support recovery from alcoholism a randomized clinical trial. JAMA Psychiatry. 2014;71(5):566-72.

49. Haug S, Lucht MJ, John U, Meyer C, Schaub MP. A Pilot Study on the Feasibility and Acceptability of a Text Message-Based Aftercare Treatment Programme Among Alcohol Outpatients. Alcohol and Alcoholism. 2015;50(2):188-94.

50. Mason M, Benotsch EG, Way T, Kim H, Snipes D. Text messaging to increase readiness to change alcohol use in college students. The journal of primary prevention. 2014;35(1):47-52.

51. Suffoletto B, Callaway C, Kristan J, Kraemer K, Clark DB. Text-message-based drinking assessments and brief interventions for young adults discharged from the emergency department. Alcoholism: Clinical & Experimental Research. 2012;36(3):552-60.

52. Suffoletto B, Kristan J, Callaway C, Kim KH, Chung T, Monti PM, et al. A text message alcohol intervention for young adult emergency department patients: a randomized clinical trial. Annals of emergency medicine. 2014;64(6):664-72. e4.
